# Marine Biodiversity in the Atlantic and Pacific Coasts of South America: Knowledge and Gaps

**DOI:** 10.1371/journal.pone.0014631

**Published:** 2011-01-31

**Authors:** Patricia Miloslavich, Eduardo Klein, Juan M. Díaz, Cristián E. Hernández, Gregorio Bigatti, Lucia Campos, Felipe Artigas, Julio Castillo, Pablo E. Penchaszadeh, Paula E. Neill, Alvar Carranza, María V. Retana, Juan M. Díaz de Astarloa, Mirtha Lewis, Pablo Yorio, María L. Piriz, Diego Rodríguez, Yocie Yoneshigue-Valentin, Luiz Gamboa, Alberto Martín

**Affiliations:** 1 Universidad Simón Bolívar, Departamento de Estudios Ambientales and Centro de Biodiversidad Marina, Caracas, Venezuela; 2 Universidad Nacional de Colombia, Bogotá, Colombia; 3 Laboratorio de Diversidad Molecular y Filoinformática, Departamento de Zoología, Facultad de Ciencias Naturales y Oceanográficas, Universidad de Concepción, Concepción, Chile; 4 Centro Nacional Patagónico, Patagonian National Center – National Research Council, Puerto Madryn, Chubut, Argentina; 5 Universidade Federal do Rio de Janeiro, Departamento de Zoologia - Instituto de Biologia, Rio de Janeiro, Brazil; 6 ULCO - Laboratoire d'Océanologie et Géosciences, Université Lille Nord de France, CNRS UMR 8187 LOG, MREN, Wimereux, France; 7 Museo Argentino de Ciencias Naturales, Buenos Aires, Argentina; 8 Universidad Católica de la Santísima Concepción, Facultad de Ciencias, Departamento de Ecología Costera, Concepción, Chile; 9 Museo Nacional de Historia Natural, Montevideo, Uruguay; 10 Facultad de Ciencias Exactas y Naturales, Instituto de Investigaciones Marinas y Costeras, National Research Council, Universidad Nacional de Mar del Plata, Mar del Plata, Argentina; 11 Wildlife Conservation Society, Argentina; 12 Universidade Federal Fluminense, Rio de Janeiro, Brazil; National Institute of Water & Atmospheric Research (NIWA), New Zealand

The marine areas of South America (SA) include almost 30,000 km of coastline and encompass three different oceanic domains—the Caribbean, the Pacific, and the Atlantic—ranging in latitude from 12°N to 55°S. The 10 countries that border these coasts have different research capabilities and taxonomic traditions that affect taxonomic knowledge. This paper analyzes the status of knowledge of marine biodiversity in five subregions along the Atlantic and Pacific coasts of South America (SA): the Tropical East Pacific, the Humboldt Current, the Patagonian Shelf, the Brazilian Shelves, and the Tropical West Atlantic, and it provides a review of ecosystem threats and regional marine conservation strategies. South American marine biodiversity is least well known in the tropical subregions (with the exception of Costa Rica and Panama). Differences in total biodiversity were observed between the Atlantic and Pacific oceans at the same latitude. In the north of the continent, the Tropical East Pacific is richer in species than the Tropical West Atlantic, however, when standardized by coastal length, there is very little difference among them. In the south, the Humboldt Current system is much richer than the Patagonian Shelf. An analysis of endemism shows that 75% of the species are reported within only one of the SA regions, while about 22% of the species of SA are not reported elsewhere in the world. National and regional initiatives focusing on new exploration, especially to unknown areas and ecosystems, as well as collaboration among countries are fundamental to achieving the goal of completing inventories of species diversity and distribution. These inventories will allow accurate interpretation of the biogeography of its two oceanic coasts and latitudinal trends, and will also provide relevant information for science based policies.

## Introduction

### The South American region

The marine areas of the South American continent extend for almost 30,000 km of coastline and encompass three different oceanic domains—the Caribbean, the Pacific, and the Atlantic. The latitudinal and longitudinal ranges within this region are similarly wide, from 12°N to 55°S, and from 34° to 81°W. Ten countries border on these coasts, each with different research capabilities and taxonomic traditions; therefore, taxonomic knowledge differs among countries. Coastal biodiversity is strongly influenced by the physical and geological history of these coasts. The eastern tropical Pacific region, which encompasses the continental coasts of southern Central America (Costa Rica and Panama) and of northwestern South America (Colombia and Ecuador) is characterized by cliffs alternating with pocket beaches, alluvial and deltaic plains with extensive sandy beaches, well-developed mangrove forests, estuaries, lagoons, and, reefs. It also includes important offshore island systems such as the Pearl and Galapagos islands [1], [2]. The Peruvian coast also is diverse with bays, cliffs, kelp and macroalgal beds, rocky shores and sandy beaches, islands, and peninsulas, as well as wetlands, which include the southernmost limit to the tropical Pacific mangrove ecosystem [3], [4]. The Chilean coast is 4,500 km of mainly rocky shores, but does include some sandy-beach bays with channels and archipelagos toward the south (Patagonian region) [5], [6]. Some of the most diverse ecosystems in Chile are the beds of kelp (*Lessonia* and *Macrosystis*) and macroalgae (*Gracillaria* and *Ulva*). The combination of the unique oceanographic conditions and coastal heterogeneity in the Chilean coast has resulted in high levels of endemism (near 40%) in many invertebrate groups [5], and several marine invertebrate taxa show latitudinal biodiversity patterns, some of them explained by the presence of Antarctic fauna [7]–[9]. Ecuador, Peru, and Chile are under the influence of the Humboldt upwelling system and subject to high environmental variability caused by the ENSO (El Niño Southern Oscillation) and LNSO (La Niña Southern Oscillation), which cause important changes in community composition and abundance, particularly of the plankton [1], [10].

The Atlantic coast of the South American continent is distinctly different from the Pacific coast. It includes three major rivers (Orinoco, Amazon, and La Plata), which discharge enormous amounts of freshwater and sediment to the ocean, and the coast has an extensive continental platform. Argentina's coast has mostly sandy beaches [6], [11] and some rocky formations located mainly at Mar del Plata and at Peninsula Valdes. At Mar del Plata, these rocky shores are dominated by two mussel species and by a diverse macroalgal community with a clear tidal zonation [12], [13]. The Uruguayan coast is dominated by sandy beaches with a narrow portion of rocky habitats known to sustain a rich biological diversity [14]. Observed variations in community composition and distribution may be related to the salinity gradient caused by La Plata River discharge [15].

The coast of Brazil, extending almost 7,500 km, is under the influence of the warm Brazil Current, the cold Malvinas/Falklands Current, and many rivers and upwelling regions [16]. The warm northern coast, where the Amazon discharges into the ocean, is characterized by a combination of freshwater, estuarine, and marine ecosystems, with diverse but poorly known habitats [17]. The colder southern coast is characterized by a variety of ecosystems, including mangrove forests, seagrass beds, coral reefs, sandy beaches, rocky shores, lagoons, and estuaries. Because of its vastness, extensive areas of Brazil's coast remain unexplored. North of Brazil are Suriname, French Guiana, Guyana, and the Venezuelan Atlantic Front. This area, including about 1,900 km of coastline, is under the strong influence of the Amazon River. Therefore, the typical ecosystems are estuaries, mudflats, sandy beaches, and mangrove forests, which extend along most of the coastline [18]. The Venezuelan Atlantic coast is also under the influence of the Orinoco River, with coastal mudflats and extensive mangrove forests [19].

In this paper, we analyze the status of knowledge of marine biodiversity in five subregions along the Atlantic and Pacific coasts of South America. As most of the information is based in national reports, these subregions were based in the Large Marine Ecosystem boundaries as defined for South America, with a few practical adaptations, based in country political borders. The paper also provides an updated review of ecosystem threats, such as invasive species, and the marine conservation strategies employed by South American countries with access to the coast, excluding the Caribbean coasts of Venezuela and Colombia, as these are included in another paper of this collection [20].

### History of research and species discovery in the region

The first studies of the South American coastal biota were carried out during a series of expeditions by European and North American researchers in the late 1700s and first half of the 1800s with naturalists Alejandro Malaspina, Roberto A. Philippi, Alcyde d'Orbigny, Alexander Von Humboldt, Aimé Bonpland, Charles Darwin, and Henry A. Pilsbry, among others [21], [22]. In the late 1800s, several other important oceanographic expeditions, including the HMS *Challenger*, collected samples along the coasts of Ecuador, Peru, Chile, Argentina, Uruguay, and Brazil [23]. In the 1900s, the Deutsche Sudpolar Expeditions in 1901–03 [24], the Swedish Lund University expedition to Chile in 1948–49 [24], the Royal Society Expedition to Southern Chile [25], the Soviet Antarctic Expedition in 1955–58 [26], and the Calypso campaigns in 1961–62 [27], [28] were among the most significant European expeditions to South America. Other important campaignsduring the second half of the twentieth century which increased the knowledge of marine biodiversity and strengthened the local research capacities were carried out by the R/V *Academik Knipovich* (1967), the R/V *Almirante Saldanha* (1966), the R/V *Atlantis II*, (1971), the R/V *El Austral* (1966–67), the R/V *Vema* (1962), and the R/V *Walther Herwig* (1966–71). At present, the oceanographic vessel *Polarstern* from the Alfred Wegener Institute (Germany) has been carrying out exploration voyages for more than 20 years to the southern regions of the continent as well as Antarctica.

In the northern latitudes of the continent, the Tropical Eastern Pacific (TEP) Biogeographic Region has a rich history of oceanographic and biological explorations dating back to the voyage of Charles Darwin to the Galapagos aboard the HMS *Beagle* in 1835 and other scientific expeditions. However, none of them visited the Pacific mainland shores and shelves of Colombia and Ecuador. It was the Eastern Pacific Expedition of the U.S National Museum of Natural History in 1904 aboard the U.S. Fish Commission steamer *Albatross* that marked the beginning of systematic oceanographic and biological studies in this region. The *Albatross* sampled zooplankton and other biological material in four shallow-water stations along the Colombian shore and nine deep-water settings off the Panamanian, Colombian, and Ecuadorian coasts. Fish, mollusks, and jellyfishes, among others, were collected and later described from these localities [29], [30], [31]. A series of research cruises and expeditions organized by North American institutions in the first half of the twentieth century contributed greatly to the knowledge of the marine fauna and flora existing in the rich area between the low tide mark and 200 m of depth in the Panama Bight, including Panama, Colombia, and Ecuador. The “Saint George” expedition visited Gorgona Island in 1927 and collected relevant material of marine organisms, particularly crustaceans [32]; the Allan Hancock cruises aboard the *Velero* III and IV vessels, dating from 1931 to 1941 (see [33]), and the Askoy Expedition of the American Museum of Natural History in 1941 also visited and collected material in Panamenian, Colombian, and Ecuadorian waters. Many new species of fishes, mollusks, polychaetes, crustaceans, and other taxa were described from material obtained from these cruises [34], [35]. A considerable number of taxonomic and ecological studies have been carried out in the last three decades in Costa Rica, Panama, Colombia, and Ecuador. However, most of this work has been geographically concentrated in a few localities such as the Gulf of Nicoya, the Bay of Panama, the Pearl Islands, the Bay of Buenaventura, Gorgona Island, and the Gulf of Guayaquil. Important collections or libraries of regional marine fauna are maintained by the Los Angeles County Museum, the Scripps Institution of Oceanography at La Jolla, California, the California Academy of Sciences in San Francisco, and the Smithsonian Tropical Research Institute (STRI) in Panama City. In the Tropical Western Atlantic (TWA), the natural history of Guyana (formerly British Guiana) was described by early explorers Sir Walter Raleigh (circa 1600) and Charles Waterton (early 1800s), who reported his discoveries in the book *Waterton's Wanderings in South America*, which served as inspiration to British schoolboys like Charles Darwin and Alfred Russell Wallace. In French Guiana, the first studies were carried out after World War II, with fish inventories and later on, in the 1950s, with the benthic (mostly shrimps) and demersal continental shelf fauna, from 15 to 100 m depth [18]. The Venezuelan Atlantic Front was until recently almost completely unexplored, and the little information available concerned commercially valuable species of fish and shrimp [19].

The local and regional academic community also had significant historic representatives. Two pioneering figures were the Uruguayan-born (1788) Dámaso Larrañaga in Uruguay and Argentina, who introduced the Linnean binomial nomenclature in the continent, and the Argentinean-born (1896) Irene Bernasconi, who studied the echinoderms. In the 1900s, research in coastal biodiversity received a strong stimulus due to the immigration of many European scientists before, during, and after World War II who contributed to knowledge and capacity building mainly through their involvement in local universities and natural science museums. Although a few research institutions were established in the region early in the twentieth century, such as STRI in Panama (1923), the most important stimulus to regional, autochthonous marine science was given by the establishment of several marine research institutions, mostly in the 1950s and 1960s. These institutions include the Instituto Oceanográfico de la Universidad de Sao Paulo in Brazil (1946), the Montemar Institute of Marine Biology (1941) founded by the Universidad de Chile and today part of the Universidad de Valparaíso Faculty of Ocean Sciences, the Instituto de Biología Marina de Mar del Plata in Argentina (1960, transformed to the INIDEP in 1977), the Instituto Oceanográfico from the Universidad de Oriente in Venezuela (∼1960), the Instituto del Mar del Perú (∼1958), the Colombian Oceanographic Commission (1968), the Colombian Science Foundation, Colciencias (1968), the departments of marine biology at universities in Bogotá (1969) and Cali (1973), the Instituto de Tecnología y Ciencias Marinas in Venezuela (1970), and the Oceanographic Institute of the Ecuadorian Navy, Inocar (1972), and the Center for Marine Science and Limnology of the University of Costa Rica (1979). These institutions changed the way that marine science was done by incorporating into the traditional taxonomic studies, time series of the environmental variables and their effect on biodiversity. In the 1960s, the Food and Agriculture Organization of the United Nations began to develop projects giving an impulse to fisheries, especially in the southwest Pacific, an upwelling zone of extraordinary productivity responsible for 20% of the world's fisheries by the end of that decade. In the 1980s and 1990s, centers for marine biodiversity research were created along the coasts of several countries, especially Brazil, Argentina, and Chile. Argentina, developed several institutions that depend on the national science council CONICET in the Patagonian region (Puerto Madryn, Ushuaia, and Bahía Blanca), while in Chile and Brazil, similar institutions are mostly dependent on universities (e.g., Valdivia and Coquimbo in Chile and FURG, the Federal University of Rio Grande, in Brazil).

Access to oceanographic vessels, isolation between researchers, and the lack of coordination between scientific programs have been an important limitation for marine research in South America [36]. The countries with the best shipping capacities are Brazil and Chile. The ships are mostly from a national navy or for fisheries research, and in some instances, access to researchers from other institutions is restricted. On the other hand, South America has benefited from regional cooperation. One example is the establishment of a common fishing zone between Uruguay and Argentina under the academic leadership of the Universidad de la República in Montevideo and the DINARA (National Direction for Aquatic Resources) in Uruguay, as well as the network of marine reserves (Red Iberoamericana de Reservas Marinas). The natural history museums in South America have been fundamental to preserving the regional marine biodiversity patrimony both in collections and in literature and are considered to be taxonomically indispensable. Some of the most relevant museums are the Museo de La Plata and the Museo Argentino de Ciencias Naturales (Argentina), the Museo de Historia Natural (Quinta Normal) in Chile, the Museo Dámaso Larrañaga and the Museo de Historia Natural in Uruguay, and the Museo de Boa Vista (Brazil). Other collections are held either at research institutions such as the STRI in Panama, the IMARPE in Peru, the INVEMAR in Colombia, or at universities, such as the Universidad de San Marcos in Peru and the Universidad Simón Bolívar in Venezuela.

### Role of the Census of Marine Life in South America

The activities of the Census of Marine Life (Census) program on the South American continent began in October 2002 with the First South American Workshop on Marine Biodiversity held at the University of Concepción in Chile. In this workshop, most of the South American countries with access to the sea reviewed the status of knowledge of their marine biodiversity (Venezuela, French Guyana, Brazil, Uruguay, Argentina, Chile, Peru, Ecuador, and Colombia). These reviews were compiled as a special issue of the journal *Gayana* in 2003. During this workshop, a regional South American Steering Committee (SASC) was established with representatives from each of the above-mentioned countries as well as representatives from OBIS, the Ocean Biogeographic Information System established by the Census. The main goal of this committee was to promote in a coordinated and well-organized way the implementation of marine biodiversity research in the South American region under the umbrella of the Census program, with particular emphasis on unexplored areas, and to integrate the regional biodiversity databases into OBIS through the creation of regional OBIS nodes located in Argentina, Brazil and Chile (http://www.iobis.org/obis/regional-nodes). Since 2002, the SASC has held several workshops, and researchers in the South American region have engaged in some of the Census projects: the Natural Geography in Shore Areas (NaGISA), the Census of Antarctic Life (CAML), the Continental Margins (COMARGE), the International Census of Marine Microbes (ICoMM), and the Mid-Atlantic Ridge Ecosystem (MAR-ECO) projects.

All of these projects have contributed significantly to increase the knowledge of marine biodiversity in the region. In the nearshore, for example, the NaGISA project has focused on the benthic diversity associated with rocky shores and on seagrass communities by using a common protocol worldwide. In the Atlantic and Pacific coasts of South America, four NaGISA sites were established at different latitudes in Argentina (Puerto Madryn and Mar del Plata), Brazil (Paranagua Bay), and Ecuador (Santa Elena). From these sites, preliminary data show that macroalgae and bivalves are the most abundant groups in the intertidal rocky shores of Argentina, while macroalgae, gastropods, and echinoderms are the most abundant groups in the intertidal rocky shores of Ecuador. In the seagrasses of Paranagua Bay in Brazil, polychaetes are the most abundant and diverse group [37], [38]. In the deep sea, on the other hand, the COMARGE project has studied the biodiversity patterns along and across the Chilean margin through a complexity of ecosystems such as methane seeps and oxygen minimum zones reporting that such habitat heterogeneity may influence the biodiversity patterns of the local fauna [39]–[41]. Furthermore, in these soft reduced sediments below the oxygen minimum zone off the Chilean margin, a diverse microbial community composed by a variety of large prokaryotes (mainly large multi-cellular filamentous “mega bacteria” of the genera *Thioploca* and *Beggiatoa*, and of “macrobacteria” including a diversity of phenotypes), protists (ciliates, flagellates, and foraminifers), as well as small metazoans (mostly nematodes and polychaetes) has been found [42]. These authors argue that the likely chemolithotrophic metabolism of most of these mega- and macrobacteria offer an alternative explanation to fossil findings, in particular to those from obvious non-littoral origins, suggesting that traditional hypotheses on the cyanobacterial origin of some fossils may have to be revised.

One of the major questions studied by the Census South American working groups on continental margins and the Antarctic was how Antarctic isolation from other continents by the Southern Ocean is relevant for understanding circulation patterns in the world oceans and atmosphere, and how biological communities have responded to past and present environmental changes. To answer this question, about 50 researchers from South America and several countries in Europe as well as the USA centralized their data in SCAR-MarBIN (Scientific Committee on Antarctic Research Marine Biodiversity Information Network) within the framework of the Antarctic-South America Interactions (ASAI) Workshop held in November 2009. This workshop provided an opportunity to exchange data and to compile an integrated document on the potential Antarctic South American biodiversity connections, taking into account all the marine realms. Results are to be published in a special issue of the journal *Oecologia Australis*.

Another regional joint effort in the region is the Latin American and Caribbean International Census of Marine Microbes (LACar-ICoMM) network launched in 2006 to evaluate the research capabilities and to identify complementary strengths and possibilities for enhanced collaboration. Artigas et al. [43] summarized some current studies on microbial diversity in both the Caribbean and South American regions. LACar has also submitted a set of samples to the ICoMM “454-tag sequencing” program in 2007, a metagenomics project especially targeting Eubacteria and Archaea in a latitudinal gradient from the southwest Atlantic (Patagonian littoral and shelf sediments and waters) to the Caribbean (Puerto Rico sediment and bays), including large estuarine systems (Río de la Plata and Amazon), and coastal brackish waters of Laguna de Rocha and Guanabara Bay. Three other projects are under way dealing with the giant bacteria of the oxygen minimum zone (OMZ) of the upwelling system in the southeastern Pacific (Chile), the bacterial diversity at different depths of the Cariaco Basin (Venezuela), and in French Guiana the bacterial diversity in the fluid muds originating in the Amazon River. Although microbial metabolism and productivity are at present being described in a variety of ecosystems in South America and the Caribbean, only scarce information on microbial dynamics and community composition is available for the planktonic and benthic realms of many coastal and oceanic regions of the area. Such information is important to fully understand topics such as biogeochemical processes and gradients in these systems that are submitted to increasing pressure from human activities and climate-change issues. The use of a wide range of available methods, techniques, and protocols in molecular biology, electron microscopy, and in situ and remote sensing facilities allow us to study all groups in a better and more systematic way. All the data collected from the Census field projects in the South American region as well as from museums, academic institutions, scientific literature, and species databases, are being integrated in the South American regional nodes of OBIS, which have contributed with nearly 300,000 records to OBIS from almost 7,000 species.

### Marine biodiversity of the South American Atlantic and Pacific regions

**Figure 1:**
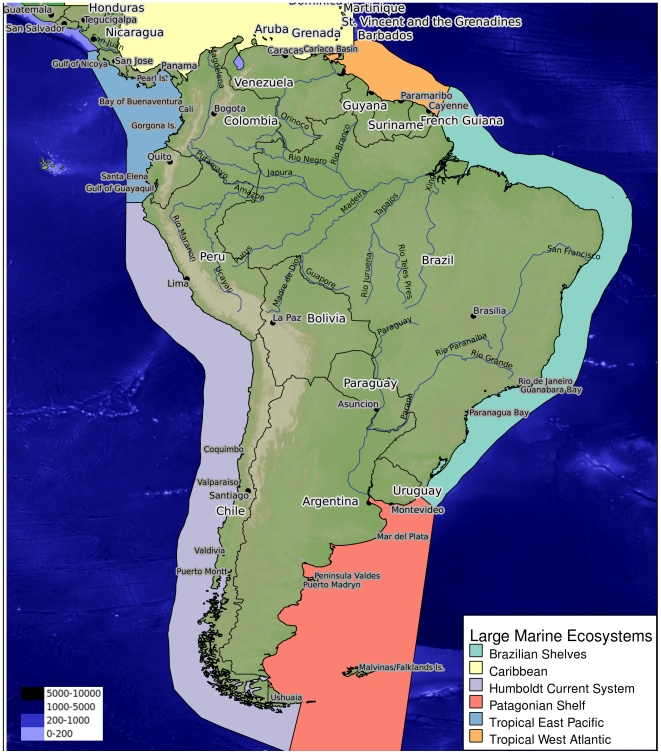
Map of South America defining the five subregions as analyzed in this paper: Tropical East Pacific (blue), Humboldt Current system (light purple), Tropical West Atlantic (orange), Brazilian shelves (light blue), and Patagonian Shelf (pink). [The Caribbean subregion (yellow) is subject of another article within this collection [20]. Bathymetry scale in meters.

This paper reviews and analyzes the marine biodiversity in five subregions of the South American Pacific and Atlantic coasts. The areas considered here are based in the Large Marine Ecosystem classification or LMEs (http://www.lme.noaa.gov/) which are defined as “areas of the ocean characterized by distinct bathymetry, hydrology, productivity and trophic interactions”, however with certain practical (political) border considerations. The subregions as reviewed in this paper are: (1) the Tropical East Pacific which includes the Pacific coasts of Colombia, Ecuador, Panama and Costa Rica, and excluding the Galapagos Islands, (2) the Humboldt Current system which includes Chile and Peru, (3) the Patagonian Shelf which includes Argentina and Uruguay, (4) the Brazilian shelves which includes the north, south, and east shelves of Brazil, and (5) the Tropical West Atlantic which includes the Venezuelan Atlantic Front, Guyana, Suriname, and French Guiana (Figure 1). The paper also assesses the research capacity in each of these five subregions as well as the threats to biodiversity and the conservation initiatives to protect it.

## Methods

The total number of species was compiled from different sources depending on the subregion, and using the OBIS database as a point of departure. Species diversity in the area corresponding to the Tropical East Pacific region (see Sherman & Hempel, 2009) was reviewed and compiled from the literature and open-access databases and sources including local, country/territory, and regional checklists and inventories, (see Table S1 for information sources). Species diversity in the area corresponding to the Humboldt Current system (Chile and Peru) was reviewed and compiled from sources including OBIS and other electronic databases such as SeaLifeBase [44] and Algaebase [45]. For Cnidaria, the database linked to SeaLifeBase provided only species names, so the taxonomy was completed using the Global Biodiversity Information Facility (GBIF) (http://data.gbif.org/welcome.htm). Other sources used were the database by Lee et al. [46], which provides information about free-living benthic marine fauna of Chile, and the species list in Castilla & Neill [47]. Species diversity in the area corresponding to the Patagonian Shelf (Argentina and Uruguay) was reviewed and compiled from OBIS through the Argentinean OBIS node AROBIS and from other electronic databases and sources. Data on vertebrate species were reviewed from publications as well as information available in OBIS (AROBIS node). These OBIS records combine published information from scientific papers and reports of pinnipeds, whales, and dolphins in the southwestern Atlantic and Magellanic region. Offshore records include reported sightings from scientific vessels and satellite tracking for seabirds, seals, and sea lions. These censuses include the distribution at or near shore waters of open coast, sheltered fjords, bays, and river mouths. Different records encompassing counting, sighting, and stranding programs, personal communications with trained individuals, photographs, unpublished abstracts from meetings, books newspaper articles, and specimen collections from academic institutions and museums (INIDEP-UNMdP) were also considered. The oldest records were accepted by the authors when the documentation and synonymy were reviewed. In addition, surveys made onboard fishing vessels provided additional biological information on targeted species and bycatch. Data on invertebrate taxa were obtained from the available literature, technical reports, databases, museum data collections, and the NaGISA project in the case of Golfo Nuevo rocky shore invertebrates. The only available, detailed and integrative compilation of reported marine invertebrate species was restricted to environments shallower than 50 m and was of limited geographical scope (Uruguayan shelf; [48]). There are no similar studies on the much larger and presumably more diverse Argentinean coast. It should be taken into account that the data presented here do not represent a revision of the identifications. Species must be evaluated through the material deposited in museum collections or by searching the species in the locality or area in which they were reported [48]. However, and although data presented must be verified by experts of each group, our results should reflect the current knowledge of marine invertebrate biodiversity in the area. Finally, data on algae, and the validity of seaweed taxa reported were checked with Algae Base [45] to update species names or higher taxonomic levels. Plankton were included in the different invertebrates groups (1,000 species were cited for Brazil and Argentina, [49]) (See Table S2 for a list of the main organizations in the Patagonian region that have contributed to knowledge of biodiversity on the regional scale and provided data sources for this revision). For the Brazilian shelf region, besides OBIS, the information was gathered with the assistance of several taxonomic specialists, and also taken from the available literature in both national and international journals, as well as many sources found in the gray literature (dissertations and theses) from major university libraries. Also, the National Council for the Development of Science and Technology (CNPq) Lattes Platform was accessed to assemble information based on Brazilian scientists' publications. Lattes Platform is a database where all Brazilian scientists are required to deposit their curriculum to gain funding for their research work. For the Tropical West Atlantic region, the data were compiled from OBIS and from a few literature sources. On the other hand, most information on threats and conservation was assembled from documents produced by the various national ministries of environment and from available scientific texts.

Information regarding microorganisms such as bacteria and phytoplankton is provided for the overall continent and is not separated by subregions.

## Results

### Subregion 1: The Tropical East Pacific – Colombia, Ecuador, and the Pacific Coasts of Panama and Costa Rica

The Tropical East Pacific (TEP) coastline is about 5,100 km long, extending from the Nicaragua-Costa Rica border (11°04′34″N, 85°41′55″W) to the Ecuador–Peru border (3°24′34″S, 80°18′25″W). According to Briggs [50], this area, including the corresponding 45,000 km^2^ of continental shelf, belongs to the TEP Biogeographic Region, which encompasses the continental shoreline and shelf that extends south of the lower end of the Gulf of California along the continental coastline down to about Cabo Blanco near the Ecuador–Peru border. It also includes several oceanic islands and archipelagos, such as Galapagos, Malpelo, Cocos, and Clipperton [50]. More specifically within the TEP, this subregion represents the southern half of the Panamanian Province, which extends from the Gulf of Tehuantepec in Mexico (22°N) to Cabo Blanco (4°S), Peru [50]. The boundaries and extent of the Panamanian Province almost coincide with those of the Pacific Central-American Coastal Large Marine Ecosystem [51]. According to the bioregionalization scheme of the world's coasts and shelf areas [52], [53], the Pacific coasts of Costa Rica and western Panama fall within the Nicoya Ecoregion, whereas the eastern half of the Pacific coast of Panama, the Colombian coast, and the northern half of the Ecuadorian mainland coast correspond to the Panama Bight Ecoregion, and the southern Ecuadorian coast and the northernmost Peruvian coast fall within the Guayaquil Ecoregion. These three ecoregions are in any case part of the TEP [52].

The morphology of the coast throughout this region is highly variable and heterogeneous, as are the features of the coastal masses. Much of the shoreline includes high cliffs with alternating pocket beaches. This pattern dominates the shorelines of northern and southern Costa Rica, central Panama, northern Colombia, and norther Ecuador. By contrast, low coasts are made of ample alluvial plains or deltas, backed by estuarine lagoons, tidal channels, and extensive mangrove swamps on mudflats [53]–[57].

The Pacific coasts of Panama, Colombia, and northern Ecuador are covered mostly by mangroves and dense rainforest vegetation. This is one of the wettest places in the world, with local rainfall of more than 10,000 mm/year on the northern Pacific coast of Colombia and very high river discharges. These conditions lead to the largest concentration of estuarine systems with high freshwater outflows of the South American Pacific, including the San Juan-Buenaventura, Patía, Mira, Cayapas, and Gulf of Guayaquil estuaries. The predominant dry climate in northern Costa Rica gradually changes toward the southeast to rainy, humid conditions in eastern Panama-Colombia and then, to the south, again to dryer climate in southern Ecuador and to arid conditions in northern Peru, where less than 100 mm/year of rainfall is recorded [55], [58], [59].

**Figure 2:**
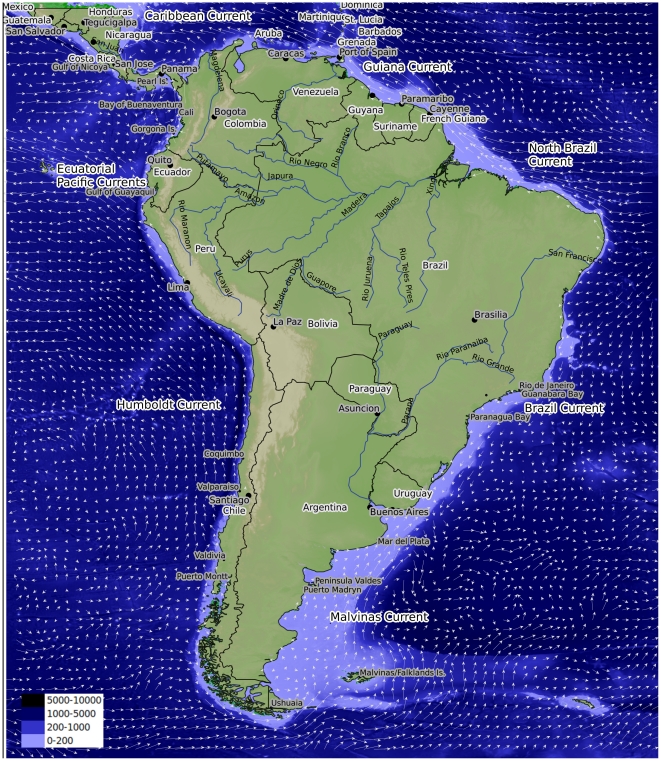
Map showing currents and bathymetry around the South American continent. Bathymetry scale in meters.

**Figure 3:**
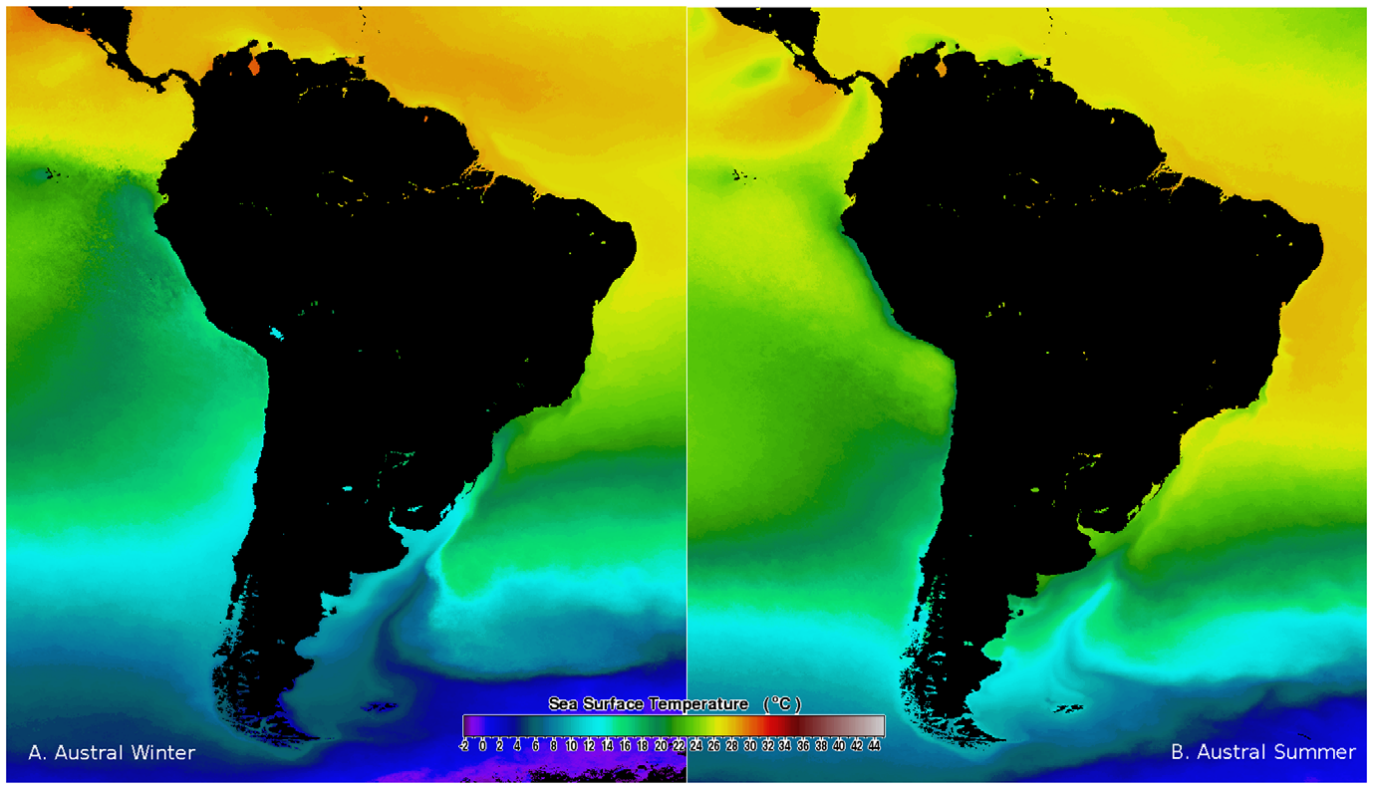
Map showing the sea surface temperature (SST) around the South American continent. A: Austral winter, B: Austral summer.

**Figure 4:**
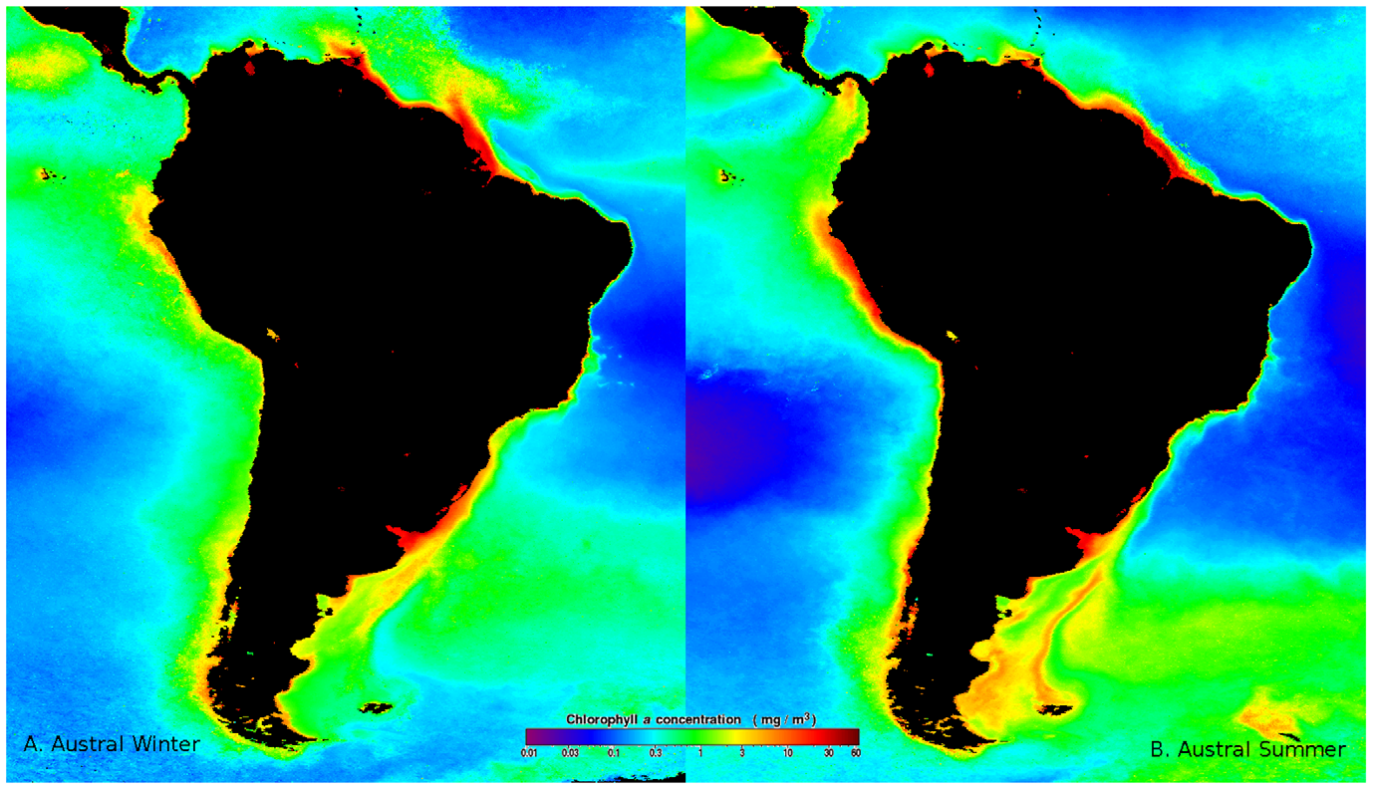
Map showing primary production measured as chlorophyll a (Chl a) around the South American continent. A: Austral winter, B: Austral summer.

Oceanic currents are rather complex in this region, with the North Equatorial Counter Current entering from the Central Pacific and a branch of the Humboldt Current, called the Colombia Current, coming in from the south. These currents create a large anticlockwise gyre in the Panama Bight and generate the Panama Current, which flows southwest toward the Galapagos (Figure 2). The northernmost coastal waters of Costa Rica are seasonally influenced by an upwelling system at the Gulf of Papagayo as well as the Gulf of Panama and adjacent areas, and the southern edge of the Ecuadorian coast is affected by the huge upwelling system along the shores of Peru [60]. The region is greatly affected by El Niño events, which occur at about four- to nine-year intervals and widely change climatic and oceanographic conditions (Figures 3 and 4). During El Niño the North Equatorial Counter Current strengthens and widens, producing a surge of relatively hot water from the central Pacific that hits the coast and substantially reduces the influence of the upwelling systems [60], [61].

The continental shelf is variably narrow in Costa Rica, western Panama and northern Colombia (less than 20 km wide). The only places where the width exceeds 100 km are off the gulfs of Panama and Guayaquil. Roughly one-third of the coastline consists of stretches of mangroves on mudflats, with major concentrations along the southern half of the Colombian and northern Ecuadorian coast and in the gulfs of Guayaquil, San Miguel, Chiriquí, and Nicoya [1], [55], [58]. There are substantial stretches of rocky shores scattered throughout the coast; the longest uninterrupted sections occur at the northwesternmost coast of Costa Rica, along the Nicoya and Osa Peninsulas, at the northernmost edge of the Colombian shoreline, and in the central coast of Ecuador. Long stretches of sandy beaches are mostly concentrated along the Costa Rican, central Panamanian, central Colombian and northern-central Ecuadorian shorelines [1], [56]–[58]. Coral reef development in this region is limited by the regular impact of El Niño events and unfavorable conditions that result from freshwater input from river runoff, siltation, nutrient enrichment, and upwelling influences [62]. The overwhelming majority of reef habitat in this region consists of rocky reefs. More suitable conditions for coral development are found around islands and rocky promontories located away from the mainland shoreline such as Isla del Caño (Costa Rica), Isla Coiba, the Pearl Islands (Panama), Isla Gorgona (southwestern Colombia), Isla La Plata, Isla Salango, and Bajo Montañita (central mainland coast of Ecuador) [63]–[66].

#### Marine biodiversity in the Tropical East Pacific: Ecuador, Colombia, Panama, and Costa Rica

At least 6,714 species-level taxa have been reported in the Pacific coastal waters of Costa Rica, Panama, Colombia, and Ecuador (Table 1, Table S3), from four Protista groups, (Foraminifera, Radiolaria, Tintinnida, Dinoflagelata), two plant phyla (algae, angiospermae), and 30 animal phyla. The quality of information was different for each of the taxa, and no information was available on bacteria, fungi, Gastrotricha, and Rotifera. This species number is constantly increasing, as new species are described every year or are recorded for the first time in the region. Knowing the taxonomic background (availability and expertise) of the region, we did not expect to be able to produce species accounts of the same quality for all the taxonomic groups. For most of the groups, the review can be considered satisfactory, but several of these counts would greatly benefit from further taxonomic review. At the phylum level, no species were reported from five phyla, and this is probably because of a lack of taxomomic attention rather than the absolute absence of these groups from the region, which is highly unlikely. Not a single species of the phyla Placozoa, Gnathostomulida, Micrognathozoa, Loricifera, and Nematomorpha has been recorded from the entire TEP region. The most diverse taxa in the region are the Polychaeta (1,894 species), fishes (1,212 species), Crustacea (863 species), and Mollusca (875 species), which together account for 47.3% of the total known biota.

**Table 1:** Summary of the diversity, state of knowledge, and expertise of the main taxonomic groups within the Tropical East Pacific subregion of South America.

Taxonomic group	No. species^1^	State of knowledge	No. introduced species	No. experts	No. ID guides^2^
**Domain Archaea**					
**Domain Bacteria (including Cyanobacteria)**	18	1	ND	0	0
**Domain Eukarya**					
**Kingdom Chromista**					
Phaeophyta	40	3	ND	4	0
**Kingdom Plantae**					
Chlorophyta	84	3	ND	4	0
Rhodophyta	183	3	ND	4	0
Angiospermae	10	4	ND	15	3
**Kingdom Protista (Protozoa)**					
Dinomastigota (Dinoflagellata)	132	2	ND	1	0
Foraminifera	164	2	ND	2	0
**Kingdom Animalia**					
Porifera	42	3	ND	2	0
Cnidaria	110	2	ND	10	2
Platyhelminthes	29	1	ND	0	0
Mollusca	875	3	2	4	3
Annelida	1894	2	1	2	0
Crustacea	863	2	ND	8	2
Bryozoa	45	1	ND	1	0
Echinodermata	223	3	1	3	1
Urochordata (Tunicata)	18	2	1	ND	0
Other invertebrates	61	1	ND	3	1
Vertebrata (Pisces)	1212	4	10	20	6
Other vertebrates	89	5		71	17
**SUBTOTAL**	6092				
**TOTAL REGIONAL DIVERSITY** 3	6714				

1 Sources of the reports: databases, scientific literature, books, field guides, technical reports.

2 Identification guides cited in Text S1.

3 Total regional diversity, including all taxonomic groups as reported in Table S3.

A few of the species recorded from this region do not have resident populations in the area or in the entire TPE, but are vagrant species that reside in the Peruvian or Galapagos provinces. These include the Humboldt penguin (*Spheniscus humboldtii*) and three species of otariid pinnipeds that have been regularly recorded in Ecuador and southern Colombia [67], [68]. In addition, under certain anomalous oceanographic conditions (e.g., strong El Niño events), the pelagic larvae of some Indo-West Pacific or Central Pacific species seem able to cross the eastern Pacific zoogeographic barrier and can succesfully settle in suitable places in the TEP. In this way the occasional records of the Indo-West Pacific crown-of-thorns starfish (*Acanthaster planci*) in Panamanian reefs [69] and the Indo-West Pacific gastropods *Mitra mitra* and *Erosaria caputserpentis* around Gorgona Island in Colombia [70], [71] can be explained.

Estimation of the number of endemic species could be accomplished with relatively high confidence for only 21 of the 68 taxa groups (31%), because information was simply not available for the remaining groups. The total number of endemic species in the region for the 21 taxa is 122, which represents only 2.18% of the species for these groups. The seemingly low number of endemics in this region is a consequence of the widespread distribution of the great majority of species beyond the Central-American Coastal region. However, at a global scale, endemism in the TEP is among the highest of any of the world's marine biogeographic regions [50]. For example, of the nearly 1,300 species of fish recorded in the TEP, about 71% are endemic [72].

With the exception of mangroves, seagrasses, mammals, birds, and reptiles, we can expect that the number of species recorded in this region will increase in the future particularly for those groups scored 1–3 (least well known) in the column “state of knowledge” in Table 1 and Table S3. However, even for relatively well known groups such as mollusks, echinoderms, and fishes, the inventories have by no means been completed, and further discoveries ought to be expected. The marine biota of the coastal waters in this region is far from being well known. Indeed, the Colombian and Ecuadorian coastal waters have been recognized as the least explored in the TEP region [1], [2], [66], [72]. The 6,700 species of marine taxa recorded at present are clearly an underestimate. The lack of comprehensive regional identification guides for most taxa is a major handicap to carrying out more accurate species inventories, and most of those that are available need thorough revisions. The OBIS database for the TEP region reports a total of 3,446 species, which is about 51% of the actual number of species reported in this review (Table 2).

**Table 2:** Comparison of the number of species per 100 kilometers of coast in the five subregions of South America contained in the OBIS database and in the present update (OBIS has a total of 13,656 species for the five subregions combined).

Subregion	Number of species Present review	Number of species in OBIS	Species/100 km of coast Present review	Species/100 km of coast OBIS	% of species in OBIS
Tropical East Pacific	6714	3446	132	68	51
Humboldt Current	10201	3894	140	53	38
Tropical West Atlantic	2743	2095	146	112	76
Brazilian Shelves	9103	5474	122	73	60
Patagonian Shelf	3776	3171	67	56	84

A total of 19 alien species belonging to six of the 68 taxa groups were registered (Table 1). The most important introduced taxa in numbers of species are the Pisces (10 species). The absence of recorded introductions of more species from other groups is indicative of the poor level of taxonomic knowledge for these groups, rather than a lack of actual introductions. The Panama Canal has provided opportunities for partial reconnection of the shallow-water faunas of the TEP and the Caribbean since 1914, particularly by freshwater-tolerant species. However, only two of the six Caribbean fishes that have entered the TEP by this method, but only one or two species (a pipefish and the Western Atlantic tarpon) seem to have successfully become resident populations there [73]. In addition, for the majority of invertebrate groups, there is often difficulty in deciding whether newly reported marine species are introduced aliens, native species that had not been formerly recorded, or cryptogenic species.

Taxonomic expertise in the region provides limited coverage. For many groups, the only currently active taxonomists work outside the region. Current local expertise is completely absent or inadequate for many important taxa, particularly those with small body sizes and little economic significance. The taxa best covered by local expertise are Angiospermae, Aves, Reptilia, Pisces, Algae, Echinodermata, and some groups of Cnidaria, Crustacea, and Mollusca. Moreover, only a small fraction of the local experts are employed as full-time systematists or taxonomists. For several groups, the coverage of available guides and identification keys is relatively good (fishes, turtles, birds, reef corals, mollusks, decapod crustaceans), although some are outdated. For all the other groups, such guides are either inadequate or completely lacking. An outstanding, collective effort for cataloging the known marine biota of Costa Rica has recently been published [74].

Inevitably, given the limited number of active taxonomists in the region, certain taxa (e.g., fish, mollusks, corals, and some crustacean groups) have received far more attention than others, whereas many others have even been completely neglected. Sampling effort has also been strongly biased toward specific locations and habitats in coastal and shallow waters (mangroves, sand beaches, coral and rocky reefs), with scarce collecting of demersal and benthic organisms in waters deeper than 100 m.

#### Threats and conservation strategies in the Tropical East Pacific

The major threats to marine biodiversity in this region are fisheries, global climate change, habitat destruction or alteration, invasive species, pollution, and human overpopulation along the coastal zone [1], [58]. The eastern Panamanian and northern Colombian Pacific are in this sense not severely affected, considering that human settlements in this area are small. However, the marine ecosystems are moderately influenced by terrestrial runoff, which has significantly increased in the last 20 years. Reefs in this area also share some common threats such as bleaching, and the live coral cover has decreased because of temperature increases of at least 1°C–2°C associated with the ENSO effect [75]. Other threats identified in this region are fisheries and occasional oil spills from ships [58], [76]. Fisheries not only pose a threat to fish and benthic invertebrate species such as shrimp, but have also proved to have detrimental effects on sea turtles, particularly on the species *Lepidochelys olivacea* and *Chelonia agassizii*, which are incidentally captured by shrimp trawling nets [77]. There are 33 Marine Protected Areas, or MPAs, in this region, including nature reserves, narional parks, and coastal wetlands of international importance, 6 in Costa Rica, 19 in Panama, 5 in Colombia, and 9 in Ecuador.

### Subregion 2: The Humboldt Current - Chile and Peru

The Humboldt Current Large region (HC) extends about 7,280 km along the west coast of South America from northern Peru (3°24′34″S, 80°18′25″W) to the southern tip of Chile (54°55′39″S, 64°52′12″W) [78], [79]. It has a surface area of 2.5 million square kilometers, containing 0.42% of the world's seamounts and 24 major estuaries [79]. The HC is one of the major upwelling systems of the world, with moderate to extremely high primary productivity (150–300 gC/m^2^/yr, Figure 4) and highly productive fisheries (e.g., in 1994, fish captures of Peru and Chile amounted to 12 million tons) accounting for 16%–20% of global fish captures [79]–[81]. This current system is characterized by cold waters that flow toward the equator, with offshore Ekman transport and coastal upwelling of cold, nutrient-rich subsurface water (Figures 2 and 3). The current system is complex and marked by coastal currents that can export waters up to 1,000 km offshore [79], [82] with subsequent effects on biological populations of species with planktonic dispersal [80]. While the northern part of the HC is affected by ENSO events, characterized by influx of warm (e.g., temperature anomaly in northern Chile 2.5°C to 5.5°C; Sielfeld et al. 2002), nutrient-depleted equatorial waters and consequent shifts in species composition [80], these events are of short duration. In fact, over the last 25 years the overall tendency of the HC has been slight cooling (−0.10°C SST; [83]).

The HC has traditionally been divided into two principal biogeographic provinces: the Peruvian Province north of 30°S, which is under subtropical influence, and the Magellanic Province south of 41°S, which is under subantarctic influence [25], [84]. Between these zones (30°–41°S) researchers distinguish a transition zone [25], [85]–[87]. In a review of 27 biogeographic classifications proposed for the southeastern Pacific coast, Camus [88] identified three consistent spatial units: a Northern Area (north of 30°S) containing a warm temperate biota (the Peruvian Province), a Southern Area (41°–43°S to 56°S) with an austral biota (the Magellanic Province), and an extensive Intermediate Area (30°S to 41°–43°S) lacking transitional elements and containing a mixed biota without a distinguishing character. In spite of the numerous efforts made to describe patterns on the Chilean coast ([89] and see reviews by Camus [88]; Fernández et al. [90]; Thiel et al. [80]), there are few studies focused on understanding the macroscale patterns of the HC, and no studies have been conducted using an explicit two-dimensional spatial analysis of biodiversity in this subregion.

Historically, the lack of studies based on georeferenced data of marine biodiversity was due to a lack of macroscale databases compiling this kind of information. However, since 2002 the Ocean Biogeographic Information System (OBIS) [91], [92] has begun to provide georeferenced data of marine biodiversity from all oceans, with access through a Web portal (www.iobis.org).

#### Marine biodiversity in the Humboldt Current: Chile and Peru

**Figure 5:**
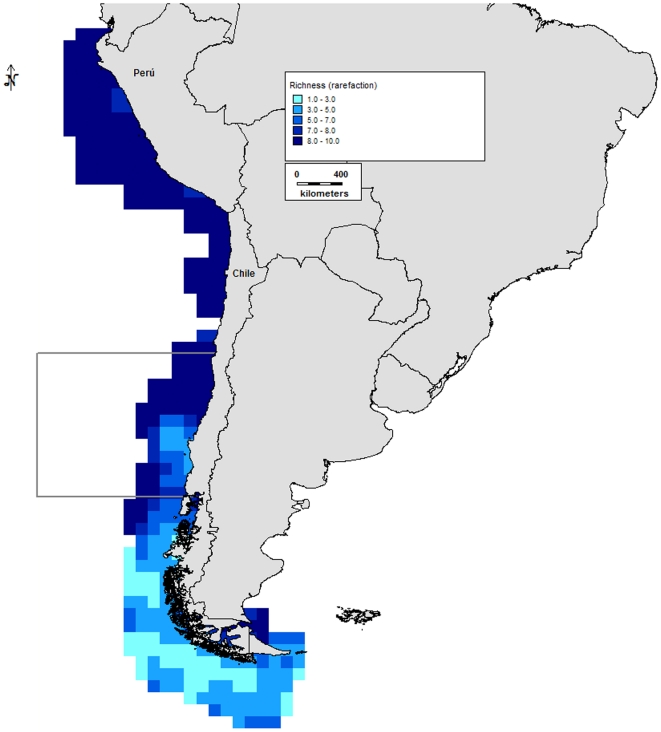
Species richness in the Humboldt Current subregion. Scale represents number of species.

Analysis of the compiled data indicates three zones of high richness for this region (Figure 5): (a) the northern Peruvian coast between 5° and 8°S, with 501 species, 270 genera, and 193 families at the point of maximum diversity; (b) the northern Chilean coast between 22° and 24°S, with 431 species, 273 genera, and 159 families at the point of maximum diversity; and (c) the southern Chilean coast between 52° and 56°S, with 522 species, 324 genera, and 188 families at the point of maximum diversity. The richness distribution was only consistent with the biogeographical limit between the previously described Peruvian Province and Intermediate Area (30°S). This limit is characterized by an area of low richness between 25° and 29°S. This pattern separates the Peruvian Province to the north, with two areas of high richness (northern Peru and northern Chile), and the Intermediate Area and Magellanic Province to the south, with one area of high richness in the southern Magellanic Province (southern Chile).

The current diversity of the HC includes 10,201 species (Table 3, Table S4). Amphipoda, Gastropoda, and Polychaeta are the taxa with the greatest number of described species, while 18 taxa do not have reliable taxonomic information (e.g., Oomycota, Loricifera). The best state of taxonomic knowledge is for Mammalia, Aves, Reptilia, Pisces, Echinodermata, and Mollusca. All of the other taxa had few, or very old, identification guides and few experts currently working in the field until very recently, when a comprehensive illustrated guide of marine benthic fauna of the Chilean Patagonian fjords was published [93]. In this book, the authors point out that the Chilean fjord region is one of the most diverse in terms of marine fauna but also the least studied. This field guide represents a 10 year unprecedented collective taxonomic effort in South America in which nearly 50 specialists from 28 institutions and 14 countries all over the world participated. The book provides identification keys for nearly 500 species from 32 taxonomic groups within 13 phyla, and reports more than 1800 species for this region.

**Table 3:** Summary of the diversity, state of knowledge, and expertise of the main taxonomic groups within the Humboldt Current subregion of South America.

Taxonomic group	No. species^1^	State of knowledge	No. introduced species	No. experts	No. ID guides^2^
**Domain Archaea**	—	—	—	—	—
**Domain Bacteria (including Cyanobacteria)**	≫15	2	ND	5	0
**Domain Eukarya**	—	—	—	—	—
**Kingdom Chromista**					
Phaeophyta	118	5	1	6	3
**Kingdom Plantae**	—	—	—	—	—
Chlorophyta	97	5	1	6	3
Rhodophyta	320	5	10	6	3
Angiospermae	ND	1	1	0	0
**Kingdom Protista (Protozoa)**	—	—	—	—	—
Dinomastigota (Dinoflagellata)	≫2	3	ND	12	3
Foraminifera	500	2	ND	1	0
**Kingdom Animalia**	—	—	—	—	—
Porifera	159	1 to 2	2	0	1
Cnidaria	517	4	1	1	3
Platyhelminthes	210	1 to 3	ND	8	1
Mollusca	1203	5	7	16	19
Annelida	649	2 to 5	8	8	6
Crustacea	3136	2 to 5	4	8	33
Bryozoa	401	5	2	2	2
Echinodermata	364	5	0	4	2
Urochordata (Tunicata)	109	5	5	4	9
Other invertebrates	776	1 to 5	0	12	19
Vertebrata (Pisces)	1167	5	35	9	4
Other vertebrates	209	1 to 5	0	37	11
**SUBTOTAL**	**9935**	**1 to 5**	**77**	**145**	**122**
**TOTAL REGIONAL DIVERSITY** 3	**10201**	**1 to 5**	**77**	**151**	**127**

1 Sources of the reports: databases, scientific literature, books, field guides, technical reports.

2 Identification guides cited in References.

3 Total regional diversity, including all taxonomic groups as reported in Table S4.

As for endemicity and alien species in the HC region, only Polychaeta, Aves, and Mammalia have records of endemic species, while 31 taxa report introduced species. Rhodophyta, Salmoniforme, and Polychaeta have the greatest number of reported introduced species. The greatest number of experts is concentrated in Mammalia, Aves, and Mollusca, while some highly diverse groups have few taxonomic experts (e.g., Polychaeta) and other groups lack taxonomic experts altogether (e.g., Nematoda, Rotifera). The taxa with the greatest number of identification guides are Decapoda and Amphipoda, while 49 taxa have only one (n = 23) or no (n = 26) published identification guides. Of these total number of described species for the HC, only 1.5% are used as fishery resources, nine of them being commercial fish species which constitute the greatest part of annual captures in the study area (i.e., *Engraulis ringens*, *Sardinops sagax*, *Trachurus murphyi*, *Strangomera bentincki*, *Scomber japonicus*, *Merluccius gayi gayi*, *Macruronus magellanicus*, *Sarda chiliensis*, and *Merluccius australis* [94]). The OBIS database for the HC region reports of 3,894 species, which is about 38% of the actual number reported in this review (Table 2). Despite the fact that the OBIS database for the HC needs to be completed considering the existing knowledge of biodiversity in this region (Table 3 and S3), it shows patterns consistent with previously described biogeographic limits and with the potential processes (e.g., ENSO, OMZ, historical glacial events) that could explain the observed differences in biodiversity between the Peruvian and Magellanic provinces. An improvement of the OBIS database will only be possible with an increase in the number of taxonomic experts to cover underrepresented taxa, together with the widespread incorporation of molecular approaches for species recognition. Nevertheless, OBIS has an advantage over other available electronic datasets given that data are georeferenced, which increases potential for the analysis of patterns and underlying processes. The incorporation of revised taxonomic data, and the investment in new coastal and oceanic expeditions will help to improve OBIS with better georeferenced data which will allow us to reevaluate the HC regional biodiversity patterns.

#### Threats and conservation strategies in the Humboldt Current

Currently, the governments of Peru and Chile have made efforts to protect the biodiversity contained in the HC through declared Coastal Marine Protected Areas [95], [96]. In Chile there are 74 areas subject to some form of marine conservation (22 officially protected areas and 52 proposals). The currently protected areas in Chile cover over 30,000 km^2^ and include five marine reserves, one marine park, six natural sanctuaries, eight coastal marine protected areas, one biosphere reserve, and one RAMSAR site. In Peru there are 14 marine and coastal protected areas comprising over 3,000 km^2^, including six natural protected marine and coastal areas, two natural sanctuaries, two national reserves, one wildlife refuge, one reserved zone, and two areas of regional conservation. These different designations translate into different degrees of protection, which vary from regulated take (e.g. regulated fishing activities) to highly restricted extraction [96]. In total, only about 1.4% of the HC is currently under some degree of protection (this value is based on the most current report of Coastal Marine Protected Areas of the Southeastern Pacific, and increases the percentage reported by Heileman et al., [79] more than twelvefold). In spite of these conservation efforts, Fernández and Castilla [95] indicate that the apparently disparate goals for conservation (i.e., exploitation of marine resources vs. preservation of marine species) pose a challenge and constraint for the formation of a network of marine protected areas.

Threats to the biodiversity of the HC include contamination and overexploitation of resources. However, while such activities can have important impacts on marine biodiversity at the local scale, the wide distibution of many species and their spatial structure as metapopulations may protect the diversity of species' populations at the regional and global scales, where these threats could cause local, but not global, extinction. Furthermore, at the global level, species invasions have been identified as an important cause of biodiversity decline [97]. Although there are few reports of highly invasive or aggressive nonindigenous species in the HC [47], we believe that the introduction of nonnative species represents a large risk to native biodiversity. The rise in the aquaculture of exotic species (mostly introduced salmonid species) and of international maritime transport in this ecosystem, coupled with deficient taxonomic and biogeographical information about native species, and the lack of explicit studies evaluating species introductions in nonpristine areas such as ports and aquaculture centers, leaves the door wide open for a potential disaster. In spite of this threat, there have been few efforts to recognize and map endemic flora and fauna of the HC and the biogeographical regions within this study area (Table 3). As mentioned above, this deficiency makes it difficult to identify nonindigenous species. A case in point is the mussel *Mytilus galloprovincialis* in Chile [47], which is a recognized invader around the world, but because of the lack of taxonomic expertise and georeferenced data, the date of introduction and current distribution in Chile is unknown. The internationally recognized problem of nonindigenous species introductions has recently been addressed in the HC where researchers and policymakers of Chile and Peru have begun to try to generate practical solutions through organizations such as Globallast and I3N-IABIN (Invasive Species Information Network – Interamerican Biodiversity Information Network).

### Subregion 3: The Patagonian Shelf - Uruguay and Argentina

The Patagonian Shelf (PS) extends for about 5,649 km along the Atlantic coast of South America from northern Uruguay (33°51′21″S, 53°11′43″W) to the southern tip of Argentina, bordering with Chile (54°55′39″S, 64°52′12″W). The area of the Patagonian Shelf extends more than 3 million square kilometers in Uruguayan and Argentinean territories and comprises coastal environments, the continental shelf and slope, and ocean basins. Its continental shelf is generally up to 100 m in depth, and is the largest and one of the most productive ecosystems in the Southern Hemisphere [98]. In the PS, two major marine currents coexists: the cold Malvinas and the warm Brazil currents (Figure 2). The former originates in the Antarctic circumpolar current and carries a high nutrient load north along the Argentine coast. The nutrient-poor waters of the Brazil current meet the Malvinas current as it moves southward along the edge of the slope [99], [100]. In the confluence or transition zone (from 30° to 46°S), a series of oceanographic phenomena (eddies, marine fronts, etc.) allow for high biological production [101] (Figures 3 and 4). Together, the coastline extension of Uruguay and Argentina measures about 5,649 km of coastline [102]–[104] and span approximately 24° in latitude; consequently, the region exhibits large topographical changes and climatic heterogeneity. Tidal regime is semidiurnal and the mean tidal amplitude varies from 0.5 m in Uruguay to over 8.2 m in the southern Argentinean Patagonia [105]. Air temperature changes seasonally in response to variations in solar radiation, cloud cover, winds, and marine currents [100]. The minimum and maximum air temperatures are −10.5°C and 39.4°C, respectively, while maximum and minimum average ranges from 3.9°C to 20.9°C. Mean wind speed varies from 14.5 to 30.0 km/h [106].

The Río de la Plata estuary represents the greatest freshwater inflow to the region, discharging on average 2.4×10^4^ m^3^/s [104], and is one of the few geographical features (i.e., Valdés Península, the Northpatagonic Gulfs, and the Magallanes Strait) that influence water circulation at a regional scale [107]. Thus, the confluence of the Malvinas and Brazil currents, together with the abundant terrestrial runoff of Río de la Plata, and the relatively shallow waters of the area, combine to produce a singular hydrographic system [53].

Biogeographically, the PS is divided into two zoogeographical provinces, the Argentinian and the Magellanic, that join around Valdés Península. The Argentine Biogeographic Province extends from 36° to 43°S, encompassing coastal or relatively shallow shelf areas off Uruguay, and the provinces of Buenos Aires, Río Negro, and Chubut in Argentina. The Magellanic Biogeographic Province, extending from 43°to 56°S, includes the coasts of southern Patagonia and the Malvinas/ Falkland Islands [108], as well as deep waters in the outer Uruguayan shelf and slope [109] and in outer Buenos Aires province. The coastal transition between both faunistic assemblages occurs around 43°–44°S. On the continental shelf, it follows a southwest–northeast direction around 70–100 m depth. In some benthic taxa (e.g., Amphipods) only 15.3% of marine benthic species known to Brazil have also been found in Argentina, suggesting that the Río de la Plata estuary may act as a biogeographic barrier for many warm-temperate and subtropical species. However, most Magellanic species that occur in southern Chile extend to the southwest Atlantic [108], [110].

#### Marine biodiversity in the Patagonian Shelf: Argentina and Uruguay

Total marine biodiversity of Argentina and Uruguay is 3,776 species, invertebrates accounting for nearly 75% of total records. Mollusca (22.5%), Crustacea (16.2%), and Pisces (14.3%) were the most diverse taxa, and together with the echinoderms, cnidarians, and macroalgae account for 65.3% of the total (Table 4 and S5). The number of species listed in the OBIS database is nearly 3,200 (Table 2), meaning that important efforts have been carried out in this region by incorporating data into the georeferenced format of OBIS. For most taxonomic groups, species records in this region need thorough revision, however, the estimated number of taxonomists devoted to invertebrates in this region is low, and most are focused on mollusks and crustaceans.

**Table 4:** Summary of the diversity, state of knowledge, and expertise of the main taxonomic groups within the Patagonian Shelf subregion of South America.

Taxonomic group	No. species^1^	State of knowledge	No. introduced species	No. experts	No. ID guides^2^
**Domain Archaea**					
**Domain Bacteria (including Cyanobacteria)**					
**Domain Eukarya**					
**Kingdom Chromista**					
Phaeophyta	59	3	1	<5	<10
**Kingdom Plantae**					
Chlorophyta	59	3	0	<5	<10
Rhodophyta	145	4	3		
Angiospermae	-				
**Kingdom Protista (Protozoa)**					
Dinomastigota (Dinoflagellata)	-				
Foraminifera	15	2	0		
**Kingdom Animalia**					
Porifera	252	3	0		
Cnidaria	258	3	1		
Platyhelminthes	36	2	0		
Mollusca	849	5	3		
Annelida	205	3	4	>30	>10
Crustacea	611	4	9		
Bryozoa	143	3	5		
Echinodermata	207	3	0		
Urochordata (Tunicata)	20	2	6		
Other invertebrates	181	2	0		
Vertebrata (Pisces)	539	4	1	>10	>5
Other vertebrates	197	5	0		
**SUBTOTAL**	**3776**		**33**		
**TOTAL REGIONAL DIVERSITY** 3	**3776**				

1 Sources of the reports: databases, scientific literature, books, field guides, technical reports.

2 Identification guides cited in Text S2.

3 Total regional diversity, including all taxonomic groups as reported in Table S5.

Globally, 129 species of marine mammals have been described, and 44 of those occur in the southwestern Atlantic. These include members of three families of Misticeti (seven species of whales) and five families of Odontoceti (27 species). From 36 known species of pinnipeds, 10 were reported for the Patagonian Shelf. Four breed in Uruguayan and Patagonian coasts, and six species have frequent or occasional presence while migrating beyond Antarctic waters. Sixteen percent of the marine mammals occurring in the southwest Atlantic Ocean are endemic or limited in distribution (La Plata River dolphin, Austral dolphin, and Commerson dolphin). Some are representatives of distant populations in the Southern Hemisphere, such as the Commerson dolphin observed in the mouth of rivers and bays in Patagonia. The southern right whale breeds in waters of the north Patagonian gulfs, the second most important reproductive area after South Africa in terms of number of animals. Species with relatively small populations but high aesthetic value, such as the killer whale, are also commonly observed in Patagonia, with only some dozens of individuals. The most important biodiversity of marine mammals has been recorded around Cabo Polonio in Uruguay and from Río Negro Province to Beagle channel in Argentina. In Río Negro the sea lions breed under the cliffs at Islote Lobos and San Matías Gulf.

Marine and coastal birds are relatively well known in the Patagonian Shelf region, where there are 147 recorded species belonging to nine orders and 24 families. Seabirds comprise over 60 species, of which penguins represent the largest biomass. This group includes 18 species that breed and feed in the shelf waters, and the rest breed in other regions, such as Antarctica or New Zealand, and use the area as feeding grounds [111]. The breeding distribution of seabirds along the Patagonian coast of Argentina and the Uruguayan coast is relatively well known, totaling close to 300 colonies of between one and eight species each [112], [113]. Highest species diversity and abundance of breeding seabirds is found in central and southern Patagonia (Chubut and Santa Cruz Provinces) and the Malvinas/Falkland Islands [113], [114]. Less is known about their distribution at sea, although surveys have been conducted in waters of the Malvinas/Falkland Islands [115] and several studies have tracked seabirds during their feeding and migration trips [116], [117]. The coasts of this region are also important feeding and resting sites for close to 20 nearctic and Patagonian migratory shorebirds, and the migratory patterns of some of them are well known. Little is known, however, about the distribution and abundance patterns of the rest of the coastal bird species. Twenty-five of the birds recorded in this PS are listed as threatened by Birdlife International.

Marine invertebrate groups from Argentina and Uruguay present great diversity and have not been studied in their totality. For example, the molluscan fauna (0–50 m) from Uruguay is composed of more than 380 marine and estuarine species [21], [118]. In front of Río de la Plata (Banco Inglés), 25 macroinvertebrate taxa were registered, including 1 ophiurid, 1 bryozoan, 4 crustaceans, and 4 polychaetes, of which the mollusks are the dominant group: 15 species, 1 Polyplacophora, 8 Bivalvia, 6 Gastropoda (1 invasive), represented by 11 families and 11 genera [119]. Exposed sites on the rocky shores of the Cabo Dos Bahias protected area (Chubut Province, Argentina), harbor a great diversity of species [120]. In San Sebastián Bay (Tierra del Fuego) 113 macroinvertebrate benthic taxa were recorded, representing 12 phyla typical of the Magellanic Biogeographic Province, [121]. In a study of the macrozoobenthos of the Beagle Channel, 32,500 organisms from 34 taxa were recorded; of which Bivalvia and Polychaeta were the most abundant, while Asteroidea and Decapoda dominated in biomass [122]. A survey on the amphipod biodiversity showed a total of 43 families, 118 genera, and 212 species registered in the Argentina and Magellanic biogeographic provinces (including Malvinas Islands) from 36° to 56°S [108]. Some 15 species of Volutid snails are endemic to the Atlantic Patagonian shelf and adjacent areas [123]. The Burwood Bank (east of Isla de los Estados) has great abundance and diversity of endemic species, including 22 species of isopods and 12 species of bivalves [21], [118], [123], [124].

Concerning regional flora, about 45% of the species occurring in the Uruguayan coast represent a southern extension of the subtropical distribution, and about 38% are a northern extension of the warm-temperate flora with several cosmopolitan species. Therefore, typical representatives of a tropical or temperate flora are equally absent in the region [125]. More information is required to gain a better understanding of seaweed diversity along the coast of the southwestern Atlantic. At present there are few taxonomists in Argentina and in Uruguay. To have good, reliable taxonomic information, it is necessary that young researchers incorporating new techniques (including environmental genetics) advance the exploration of poorly studied areas.

#### Threats and conservation strategies in the Patagonian Shelf

Within the the Patagonian Shelf region, Sullivan and Bustamante [53] ranked the Uruguay–Buenos Aires Shelf ecoregion high in biological importance and need for conservation actions, because the area presents high biological productivity, abundant populations of finfish, and numerous marine mammals and seabirds that feed upon those fish. Intensive fisheries in the Patagonian region are limited to a few species of fishes and invertebrates, and 10 species (seven fish, one squid, one shrimp, and one bivalve) represent 85% of the catch [98], [104], [126]. At least 15 species that inhabit this region, mainly birds and mammals, provide some of the greatest examples of marine fauna on the planet [117]. As top predators, these species play key and varied roles in the marine ecosystem. Albatrosses, petrels, penguins, sea lions, and elephant seals require large areas and abundant food supplies for their survival. The International Union for Conservation of Nature (IUCN) has evaluated 223 species from the Patagonian region, and of these, 65 species are actually endangered, 39 of them fishes, 5 mammals, 16 birds, and 5 turtles [98].

In general, major threats to marine biodiversity include fisheries overexploitation, habitat deterioration, and invasion of exotic species. The most serious threats to vertebrates are overfishing, bycatch of seabirds, marine mammals, and turtles, as well as degradation of coastal and marine environments, urban pollution, and pollution from industrial activities such as fishing and oil exploration, exploitation, and transport. Threats to marine invertebrates biodiversity include degradation and disturbance of environments, urban development in coastal areas, dredging, resuspension of sediment, establishment and operation of ports, presence of exotic species, tourist use, global and local aquatic contamination, fisheries targeting for invertebrate species or bycatch resulting from dredging [123]. Activities carried out with bottom nets are also responsible for modifications in the communities, which are generally slow to recover, even after the activities stop. Bottom trawling dominates coastal and deep-sea fishing and produces large amounts of discards of benthic invertebrates, equivalent to 80% of the catch [127]. Bycatch affects at least four species of marine turtles, some 20 species of birds, and seven species of mammals (sea lions, elephant seals, and dolphins) as well as fish and marine invertebrates. For example, an estimated 7,000 albatrosses and petrels belonging to 12 species were killed as a result of interaction with longline fishing vessels between 1999 and 2001. In the hake fishery, 37 species of fish, crustaceans, and mollusks (including the Argentine squid, *Illex argentinus*) are caught and discarded. Between 35,900 and 42,000 tons of hake were caught in 2002 as bycatch in the trawl fishery targeting the Argentine red shrimp, *Pleoticus muelleri* [126]. In Uruguay, 55 species of macroinvertebrates were recorded in the fisheries of the volutid *Zidona dufresnei*. The fishery targeting for the scallops *Psichrochlamys patagonica* and *Aequipecten tehuelchus* is the largest scallop fishery in the world, with catches of more than 11,000 tons in 2006, exploiting banks with a total area of 11,250 km^2^ [127].

In recent years, a series of biological invasions including algae, mollusks, hydroids, bryozoans, ascidiaceans, and crustaceans occurred in marine environments because of involuntary transport or voluntary introduction, always with severe consequences not only for the local biodiversity but also from an economical perspective [123], [128]–[130]. This problem constitutes a serious threat to biological diversity in the area. At least 41 non-native species have been recorded, especially invertebrates and algae [128]. *Undaria pinnatifida* is a successful invasive seaweed widespread along a large area of the coast of Patagonia. Its presence is associated with a dramatic decrease in species richness and diversity of native seaweeds. This impact should be considered not only from a biodiversity point of view but also from an economic perspective [131]. *Undaria* has been found widespread in populations of the agar-producing red alga *Gracilaria* and recently was reported settled on shellfish commercial beds (M.L. Piriz, personal communication). Even when native sea urchins feed on *Undaria*, they are unlikely to play a role in the control of this kelp [132].

In Argentina, there are currently 45 coastal and marine protected areas aimed at protecting marine or coastal resources [133], [134]. The strong interest in coastal resources has resulted in the designation of protected areas in which the extension of marine environments is in general relatively small or simply lacking [134]. Thus, only 16 of these protected areas include adjacent waters, while the rest protect exclusively terrestrial environments on the coast. However, these coastal protected areas include marine organisms, such as seabirds and marine mammals, among their main conservation targets. Recent initiatives, led mainly by the National Parks Administration of Argentina, are focusing on the designation of new marine parks that include larger areas of marine waters. In the Malvinas Islands, there are 17 natural reserves with significant coastal habitat [98].

In Uruguay, there is an incipient process to implement the first Marine Protected Areas. The newly developed National System of Protected Areas is responsible for this process, and there are currently three coastal areas considered (Santa Lucía, Cabo Polonio, and Cerro Verde). In addition, there are proposals for a network of marine protected areas [104]. The banning of hunting in the 1960s was the first national strategy for the conservation of marine mammals in Argentina. Then, emblematic species such as the southern right whale prompted specific protective initiatives such as National Natural Monuments (Law 23.094/84). Uruguay (1998) also adopted the protection and conservation of cetaceans and pinnipeds. Relevant actions for conservation are aimed at the creation of more protected areas, development management, and mitigation plans, including education and scientific research. For benthic species, the most important feature requiring urgent conservation is the habitat, which can be done by avoiding or minimizing the effects of the dredging nets. Recently, ecosystem-based fishery management and Marine Protected Areas are emerging as promising tools to conserve marine environments, in view of declining fisheries indicators in the region [104], [135], [136]. In this sense, the Secretary of Environment and Sustainable Development and the Federal Fishery Council of Argentina recently (2009) banned “totally and permanently” fisheries activities in the Burwood Bank (www.ambiente.gov.ar). This zone presents high biodiversity and endemism, and the policy is in agreement with the conservation of marine bottom environments in relation to Argentine commitments with UN Food and Agriculture Organization. An international, ecoregional conservation program will contribute to the continuity of the ecological processes supporting the rich biodiversity of this subregion. This will be critical to ensure ecosystem resilience and adaptation to a changing environment, maintaining ecosystem processes and sustainable use of marine resources.

### Subregion 4: The Brazilian Shelves - North, South, and East

Brazil has the longest coastline in South America, extending 7,491 km on the Atlantic coast of South America from Brazil's border with French Guiana in the north at Cape Orange (4°20′20″S, 51°22′12″W) to its southern border with Uruguay at Chuí (33°51′21″S, 53°11′43″W). Its territorial sea includes the 12 nautical miles from the coastline, the maritime zone that begins in the coastal region, including the marine continental shelf and the exclusive economic zone that extends 200 nautical miles from the coast. Besides this area, Brazil has successfully pleaded to the United Nations for an addition of 900 km^2^ where the continental shelf extends beyond the 200 nautical miles based on the UN Convention on the Law of the Sea. This means that the Brazilian jurisdictional waters now comprise 4.5 million km^2^ and have been designated by the Interministerial Committee on the Sea Resources (CIRM, acronym in Portuguese) as the “Blue Amazon.”

The Brazilian continental shelf and margin are very heterogeneous. The shelf is narrowest in the Northeast Region (8 km off Recife) and widest both off the Amazon River in the north (∼300 km), and in the south off Rio Grande do Sul (246 km). Apart from the Amazon, there are other important river outflows such as the São Francisco in the Northeast Region, the Pardo, Doce, and Jequitinhonha in the central part of the country, Paraíba do Sul, and the combination of the La Plata and Patos Lagoon outflows in the South Region [137]. Also, the continental shelf breaks at different depths depending on the region: 80–100 m in the North Region; 60–70 m in the Northeast and northern Southeast regions from the Vitória-Trindade ridge to the north; 160–200 m in the southern part of the Southeast and South regions. Around 70% of the Brazilian exclusive economic zone defined between 12 and 200 miles off the coast is within the slope and abyssal zones. The slope is much steeper in the Northeast and Southeast regions than in the North and South regions and also comprises a variety of deep-sea canyons, cold corals, and cold seeps.

The western South Atlantic including its seamounts and topographic ridges has been formed since the opening of the Atlantic Ocean around 110 million years ago. The northern Brazilian margin has several major topographic highs that form the North Brazilian Ridge and several scattered seamounts rising from the ocean floor. These constrain the North Atlantic Deep Water flow, causing turbulence and upwelling due to the seamounts topography [138]. Large erosional and accretionary forces in the Amazon River mouth, caused by water boils, crosscurrents, eddies, and tides, result in unstable channels and banks with few stable points [139]–[141]. Fluid muds occur on the inner shelf north of the river mouth. However, south of the Amazon mouth, the lack of sediment influx has resulted in a complexly embayed erosional coastline [142]. The Amazon Fan area is stable tectonically, with subsidance rates of 5–20 cm in a thousand years, but it is not quiescent. Numerous earthquakes within the last 20 years have recorded magnitudes of 3.0 to 4.8 [163]. Besides earthquake activity, near-surface faults and large methane gas deposits also create unstable seabed conditions [143]. High-resolution seismic profiles near the shelf edge show evidence of near-surface slumps and faulting 20–50 m in the subsurface and concentrations (about 500 m^2^) of methane gas [143]. Several studies (e.g., Amazon Shelf Study—AMASEDS, LEPLAC, REMAC, GLORIA, Ocean Drilling Program—ODP) indicate that there is evidence for gas seepage on the slope off the Amazon fan based on the incidence of bottom-simulating reflections (BSRs), mud volcanoes, pock marks, gas in sediments, and deeper hydrocarbon occurrences. The existence of methane at relatively shallow depths and extensive areas of gas hydrates have been mapped in this region. Also, gas chimneys have been reported, and exploratory wells have discovered subcommercial gas accumulations and pock marks along fault planes. A sound geological and geophysical understanding of the Foz do Amazonas Basin is already available and used by the energy companies.

A major oceanic plateau occurs off the eastern boundary of the Amazon cone: the Ceara Rise. The Fernando de Noronha Ridge formed by a seamount ridge and basement highs occurs at the western extremity of the Romanche Trench off the Northeast Region of Brazil. Along this ridge, the Atol das Rocas is on the western side of the flat top of a seamount, and oceanic basalt outcrops form the Fernando de Noronha Island at the eastern extreme of this ridge. Basaltic rocks are close to the surface at the Atol das Rocas, but only shallow-water carbonates outcrop [144]. This is one of the first marine protected areas created in Brazil because of the intense bird and turtle activities and also rich marine life [144]. Many other seamounts, such as the Pernambuco and Bahia seamounts, occur along fracture zone lines farther south.

The Victoria-Trindade Ridge comprises seamounts arising from the Brazilian continental margin toward the Mid-Atlantic Ridge, with volcanic rock outcroppings at Trindade and Martin Vaz oceanic islands at the eastern extremity of this chain, about 1,050 km from the continent. Between the continental margin and Trindade, the other seamounts on this ridge rise from around 5,000 m in the southwest Atlantic abyssal plain, but have fairly shallow summits at depths of 34–76 m. Along the eastern Brazilian continental margin, several plateaus can be found, but the major ones are the Abrolhos Bank and Pernambuco Plateau, and smaller ones such as João Pessoa and Rio Grande do Norte Plateaus.

The large Sao Paulo Plateau is in the southern region off Brazil, and its southern edge is formed by a sharp volcanic ridge with more than 2,000 m relief and with several seamounts at its eastern boundary [145]. According to these authors, a broad aseismic ridge occurs to the southeast of the São Paulo plateau. These topographic features also form a major barrier to the Antarctic Atlantic Bottom Water (AABW), which flows northward through the Vema channel [146], [147]. According to Campos et al. [138], major upwelling and turbulent submarine flows are likely to occur on the flanks of these topographic highs, and the occurrence of cobalt crusts and manganese nodules can be expected in the abyssal areas.

The climate of the Brazilian coast generally depends on the South Atlantic tropical and polar anticyclones, the latter with its cold air mass originating in southern Argentina [148], or in the Weddell Sea in the Antarctic region (Aquino personal communication). Over the last few centuries, the wind regime oscillation has been the major factor causing water temperature variability [149]. This also greatly influences the displacement of water masses and the occurrence of eddies and upwellings of seawater in the subantarctic (South Atlantic Central Water) especially in the Southeast and South regions of Brazil [148].

Meridional temperature gradients characterize the South Atlantic, where the sea surface temperature increases with latitude and decreases toward the southern region [150]. Warmer temperatures from the South Equatorial Current dominate the margin north of the Vitória-Trindade Ridge at the north-northeastern border where they meet cooler waters from the North Equatorial Current. South of the Vitória-Trindade Ridge, water masses are more stratified as the southward flow of the Brazil Current encounters the subtropical gyre south of Rio de Janeiro [151]. Each year, during the first semester, five water masses are dominant at 20°S: (1) the Tropical Water (TW) from surface to 200 m (22°C–27°C and salinity 36.5–37); (2) the SACW from 200 to 660 m (6°C–18.5°C and salinity 34.5–36.4); (3) the Antarctic Intermediate Waters (AIW) from 700 to 1,200 (4°C–10°C and salinity 34.2–34.8); (4) the North Atlantic Deep Water (NADW) from 1,200 to 2,000 m (3°C–4°C and salinity 34.6–35); and (5) the Atlantic Antarctic Bottom Water (AABW) at abyssal depths (0.5°C and salinity 34.60) [151]–[154] (Figure 3).

The Brazilian continental margin is strongly influenced by the western contour currents. There are two major contour currents detected at the surface: the Brazil Current (BC) flowing southward and the Brazilian Northern Current (BNC) flowing northward [137]. The BC, which is shallowest between 15° and 20°S, transports saline, oligotrophic tropical waters, and as it reaches the Vitória-Trindade Ridge, it receives additional contribution from the South Atlantic Central Waters (SACW), reaching a vertical extension of about 500 m, and continues to flow southward toward the Subtropical Convergence (33°–38°S) where it merges with the Malvinas Current and then flows away from the coast to the east [155] (Figure 2).

The BC changes direction near Cabo Frio in the state of Rio de Janeiro as a wind-driven process following the continental margin to the southwest and causing eddies throughout the year [156]. This process promotes the upwelling of the SACW, which is rich in nutrients [157], [158], enhancing fisheries biodiversity and biomass in the region [159]. The BC increases in volume as it reaches the south of Cape Santa Marta Grande because of the intermediate portion of the subtropical gyre circulation (500–1,200 m). The AIW is transported at this depth range, and the BC becomes more than 1,000 m thick as it flows through the South American Atlantic southern continental margin [160]. The AIW receives the Intermediate Contour Current (ICC) at intermediate levels around 28°S. The ICC flows northward, contours the Vitória-Trindade Ridge, and receives a contribution at the level of the Southern Equatorial Current branch at 19°S, forming the Brazilian Northern Subcurrent (BNS). This transports the SACW and AIW toward the equator, and it strengthens toward the northern part of Cape Branco in Paraíba as a result of its fusion with the BNC and equatorial branches of the South Equatorial Current [161]. This allows the BNC to cross the equator moving away from South America at 10°N. According to Vink et al. [161], the Brazilian North and Northeast regions are strongly influenced by the BNC.

The BNC reaches speeds of 1–2 m/s, forcing the Amazon River water and sediments to the northwest. The Amazon shelf in itself is a dynamic region, and dominated by the effluent of the Amazon River, which has a mean annual transport of approximately 1.8×10^5^ m^3^/s of freshwater flowing into the Atlantic Ocean [162] and depositing a daily average of 3 million tons of sediment near its mouth [142], [163]. The annual outflow from the river accounts for 20% of all the freshwater that drains into the oceans of the world [164]. Waters from the Amazon River can migrate as far north as Barbados and as far as 320 km offshore.

The South Atlantic is possibly a major corridor to the deep Atlantic oceanic circulation with the northward flow of the AABW, which originates especially in the Weddell Sea, and the southward flow of the NADW above it [152]. The latter greatly contributes to the circulation toward the east and upwells at the Antarctic Divergence at 60°S. The circulation of water masses, especially the deep-water circulation, is greatly influenced by all topographic features along the Brazilian continental margin and the presence of adjacent seamounts. The southwest Atlantic thermocline is well marked with its upper limit between 50 and 100 m, but its depth varies depending on latitude and season, being deeper in the winter at highest latitudes. Near the seamounts with shallow summits (e.g., those at the Vitória-Trindade Ridge or at the North Brazilian Ridge), local turbulence because of the upwelling effects disturbs the thermocline [150, and authors therein].

Considering the heterogeneity of the Brazilian continental shelf, margin, adjacent seamounts, and abyssal plain, the very large Brazilian marine ecosystem [165]–[168] is hydrologically and topographically complex. In fact, it has contrasting dominant ecosystems of unique features, including mangroves, coral reefs, dunes, sand banks, sandy beaches, rocky shores, lagoons, estuaries, and salt marshes, all of which host an uncountable number of flora and fauna species with high levels of endemism. Some species are in danger of extinction, while others are detected as being invasive. Despite its low productivity (less than 150 gC/m^2^/y, based on SeaWiFS global primary productivity estimates) (Figure 4), this whole “Blue Amazon” has a high marine biodiversity [167], and its deep seas include a variety of ecosystems such as canyons, gregarious kelp, coralline and sponge systems, pock marks, seamounts, and abyssal plains with manganese nodules and other mineral resources [138], [169]–[174].

#### Marine biodiversity in the Brazilian Shelf

A total of 9,103 species have been reported in Brazilian waters (Tables 5 and S6). The most diverse taxa in the region's marine coastal waters are the crustaceans (1,966 species), followed by the mollusks (1,833 species), the fishes (1,294), and the polychaetes (987 species), which together account for 66.79% of the total known biota. While most of the available information on marine biodiversity is about the continental shelf, Brazil also has a number of significant publications on the slope, the seamounts and oceanic islands, and the abyssal plains (Table S7). These publications derive from many cruises along the Brazilian coast, deeper stations mainly at the southeast offshore, but also deep-sea fishing in the North and Northeast regions (Table S8). Most of the deep-sea research has been relatively recent (since 1986) and focused on fish, macrobenthic invertebrates, and zooplankton, while the best-studied areas have been the Campos Basin, the North Brazilian Ridge, Fernando de Noronha, and Vitória-Trindade Ridge. As for the continental shelf, most of the knowledge on marine biodiversity has been gathered from the north of Brazil, part of the northeastern coast, and those from the southern regions derive from the continental shelf shallow waters. The Brazilian continental shelf, like most shelves around the world, is subject to growing pressure from human activities and holds the majority of fisheries resources [175]. There are several articles on the taxonomy, phylogeny, biogeography, biology, and ecology of many marine organisms, and also community data available from major national programs such as the REVIZEE (Assessment of the Sustainable Potential of Living Resources of the Brazilian Exclusive Economic Zone), which encompassed the whole of the Brazilian coast. Some examples are provided in Table S8. Also, many studies are regional and include several topics from taxonomy to marine communities, oceanography studies, and conservation. An example of a comprehensive study is the OPISS (Oceanografia da Plataforma Interna de Sao Sebastiao), which was carried out at the Sao Sebastiao Continental Shelf on the northern coast of Sao Paulo State [175]. This region is subject to a complex hydrological regime with physiographic features determined by its proximity to the Serra do Mar (mountains dominated by Atlantic Forest), the presence of Sao Sebastiao Island, and the development of one of the most important oil and gas terminals in Brazil [175]. Other fairly well studied areas are the Guanabara Bay in Rio de Janeiro State [176]–[188]; Ubatuba [189]–[192], Cananéia in São Paulo State [193], [194]; and Paranagua Bay in Parana State [195]–[201].

**Table 5:** Summary of the diversity, state of knowledge, and expertise of the main taxonomic groups within the Brazilian Shelves subregion of South America.

Taxonomic group	No. species^1^	State of knowledge	No. introduced species	No. experts	No. ID guides^2^
**Domain Archaea**					
**Domain Bacteria (including Cyanobacteria)**	2				
**Domain Eukarya**					
**Kingdom Chromista**					
Phaeophyta	106	4		8	
**Kingdom Plantae**					
Chlorophyta	201	4		8	
Rhodophyta	488	4		8	
Angiospermae	14	5			
**Kingdom Protista (Protozoa)**					
Dinomastigota (Dinoflagellata)	49				
Foraminifera	15				
**Kingdom Animalia**					
Porifera	400	3		15	2
Cnidaria	535	4		35	10
Platyhelminthes	45	2			
Mollusca	1833	2 to 4	2	36	7
Annelida	987	4	8	23	5+1 in prep.
Crustacea	1966	3		6	
Bryozoa	133	2			
Echinodermata	254	3 to 4		13	
Urochordata (Tunicata)	70	2			
Other invertebrates	308				
Vertebrata (Pisces)	1294	4		4+	3
Other vertebrates	178	4 to 5		40	2
**SUBTOTAL**	**8878**		**10**	**196**	**29**
**TOTAL REGIONAL DIVERSITY** 3	**9103**				

1 Sources of the reports: databases, scientific literature, books, field guides, technical reports.

2 Identification guides cited in References and in Table S7.

3 Total regional diversity, including all taxonomic groups as reported in Table S6.

Collections of marine organisms exist at several important institutions throughout Brazil, such as Museu Emilio Goeldi (North Region); LABOMar (a marine laboratory at the Universidade Federal do Ceará), Universidade Federal de Pernambuco and Universidade Federal Rural de Pernambuco, Universidade de Mossoró (Paraíba), all in the Northeast Region; Museu Nacional and Instituto de Biologia at the Universidade Federal do Rio de Janeiro; Museu de Zoologia, Departamento de Ecologia Geral (Instituto de Biociências), Instituto Oceanográfico at the Universidade de São Paulo, SP, and Museu de Zoologia da Universidade Estadual de Campinas “Adão Jose Cardoso” (Southeast Region); Departamento de Zoologia at the Universidade Federal do Paraná, and the Museu Oceanográfico (Fundação Universidade do Rio Grande, Rio Grande do Sul) in the South Region. Also, several species lists and illustrated guides and manuals have been produced recently including reviews on the biodiversity of the ecosystems in the continental shelf [202]–[221].

According to the REVIZEE program, the Brazilian continental shelf and slope (down to 2,076 m depth) have been divided into four sectors called “scores”: North, Northeast, Central, and South. In each of these scores, extensive surveys have been carried out to estimate the diversity and abundance of planktonic, nectonic and benthic organisms and their sustainable exploitation potential [212], [215], [222], [223].

In the Brazilian North score, the freshwater from the Amazon River, rich in nutrients, is responsible for the highest primary production in the country (more than 300 gC/m^2^/yr, based on SeaWiFS global primary productivity estimates) [168], [167]. Most of what is known about marine biodiversity in the north is related to fishing, mangrove habitats, and data obtained through the REVIZEE program. About 30% of Brazilian fishing takes place in the North Region, where Pará is the country's second-largest landing port [224]–[226]. Harvested species include catfish, corvina, sawfish, red porgy, lobsters, and prawns. The region includes one of the main shrimp banks in the world, extending from Tutóia in Maranhão to Orinoco in the Guiana, mainly because of its extensive mangrove areas [227], [228]. The mangroves sustain high biodiversity of estuarine and marine organisms and represent important nurseries for many species of fish, feeding grounds for some marine mammals such as the manitees, and a nesting place for many species of seabirds [229], [203].

The Northeast score accounts for about 12% of the national fishing (about 70,000 tons per year) and this fishing can be divided into two groups: coastal fishing mainly on the continental shelf, and fishing near islands and oceanic banks [230]–[235]. The oceanic fishing is dedicated to tunas [169], [236]–[243]. Dog snaper, dentex, sawfish, red porgy, flying fish, mackerel, and dorado are among the most important fish landed by artisanal fisherman in the region [230]. Shrimps, prawns, and lobsters are captured in trawling nets and are exploited to the sustainable limit [178], [244]–[246]. *Panulirus argus*, *P. laevicauda*, *P. echinatus*, *Syllarides brasiliensis*, and *S. delfosi* are economically important, but only the first two have fishing restrictions. Crustaceans and mollusks are considered important resources in the Northeast Region. According to Alves and Nishida [247], the crab *Ucides cordatus* (Linnaeus, 1763) or “caranguejo-uçá,” as it is known in Brazil, is one of the most conspicuous and abundant components of the Brazilian mangrove ecosystems epibenthic macrofauna, and the most exploited resource by artisanal fisheries, especially in the Northeast Region. The scientific interest in other marine organisms, which inhabit different ecosystems in the region, is supported by local federal universities and research centers.

The Central score is characterized by the presence of coral reefs and calcareous algae. The Abrolhos Bank on the southern coast of Bahia State is the largest coral bank in the South Atlantic (70,000 km^2^) with more than 16 stony corals recorded [248]. Edged by Atlantic forest, the bank comprises a mosaic of coastal marine environments, including coral reefs, algae bottoms, mangroves, beaches, and sand banks [170], [249], [250]. The highest biodiversity in the South Atlantic is found in this area; Abrolhos shelters not only many endemic species such as the brain coral, but also crustaceans, mollusks, sea turtles, and marine mammals (especially cetaceans) [251]–[253]. Nonarticulated calcareous algae found in this region attach to various substrates. As this region is generally oligotrophic and has different water masses including that of the Atlantic Central Waters, which are coldest and rich in nutrients, a rich diversity of macroalgae benefit from these hydrological conditions. These macroalgae include mainly the tropical orders Cladophorales, Bryopsidales, Dyctiotales, Fucales, and Ceramiales, among others [254], which are also usually found in the Caribbean Sea [255]. Conversely, many species with temperate affinities and found only in areas under the influence of the subantarctic-originated Atlantic Central Waters, such as the kelp *Laminaria abyssalis* [256], the geographic distribution of which extends from the northern part of Cabo Frio in Rio de Janeiro State to the mouth of Rio Doce River in Espírito Santo State [257], [Yoneshigue-Valentin personal observation]. The region is also characterized by endemic species of the kelp *Laminaria abyssalis* and the agariferous *Gracilaria abyssalis* and is abundant in economically important rhodolites formed by calcareous algae. About 774 infrageneric taxa of marine macroalgae (482 Rodophyta, 191 Chlorophyta, 101 Heterokontophyta) are so far known for the whole Brazilian coast. Regarding fisheries, Serraniids, groupers, and other species of fish that live in reefs and rock bottoms, and also pelagic fish are often caught in the shores of southern Bahia and also Espírito Santo State. Cabo Frio, Niterói, and Angra dos Reis in Rio de Janeiro State are other important landing ports in the Central score. The artisanal fishing is significant for prawns, corvine, mullet, and cutlass in certain areas such as the Guanabara Bay, Sepetiba Bay, Ilha Grande, and Parati in Rio de Janeiro State.

About 185 species of fish have been identified from the Southern score. There are many landing ports (Rio Grande, Itajaí and Navegantes, Santos and Guarujá) in the South Region, and fishing control is harder in this region. In contrast to the Northeast Region, artisanal fishing in the South represents only about 15% of the regional production [258], [259]. But artisanal fishing with bottom trawling is common in São Paulo, Paraná, and Santa Catarina states, where the main fishing targets are prawns, corvinas, hakes, soles, engrauliids, and mullet [260], [261]. Prawns and crabs are heavily fished in Patos Lagoon in Rio Grande do Sul State, and at its coastline the fishing industry aims at corvinas, hake, anchovies, sardines, shark, skate, and dogfish, among others [258]. There are several important field guides and manuals related not only to pelagic organisms but also to benthic ones (e.g., sponges [262], [263], [264], polychaetes [265], [266]).

#### Threats and conservation strategies in the Brazilian Shelf

Over the years, the vast extent of the coastline and the variety of coastal marine ecosystems in Brazil gave rise to the public perception of inexhaustible sea resources. This perception led to policies that encouraged unsustainable use of resources. As a result, although marine fisheries contribute 63% of the total fish production in Brazil, over 80% of the resources are currently overexploited [267], [268]. On the other hand, the fishing industry in Brazil is responsible for generating approximately 800,000 jobs, apart from providing animal protein for human consumption. This means the fishing industry has enormous social and economic importance affecting some 4 million people who depend directly or indirectly on this sector [269]. Brazilian legislation defines the coastal zone as a national patrimony that includes also the 12 nautical miles of territorial sea. Coastal management is conducted by a national plan legally enforced, complemented by state and county plans, and by coastal ecologic-economic zoning limited to small portions of the coastal zone [270]. However, only a small portion of the enormous Brazilian coastline is under some form of protection or management, and there are large areas under anthropogenic pressures [271]. Considering the high levels of endemism of Brazilian marine organisms, and the likelihood that the growing population will exert even higher anthropogenic pressures such as fishing, large-scale conservation and management plans are urgently needed. Some efforts have been undertaken with management from different societal sectors and with background information provided by the scientific community [272]–[274].

Considering all the factors mentioned above, Brazil faces the difficult tasks of identifying, inventorying, and scientifically studying all its biological diversity (terrestrial and marine), as well as developing and implementing management and sustainable use mechanisms [267], [268]. The government's primary formal mechanism for guaranteeing the conservation of Brazilian biodiversity is the Convention on Biological Diversity. This convention was adopted and approved during the United Nations Conference on Environment and Development, held in Rio de Janeiro in June 1992. As a prime mover in these negotiations, Brazil was the first signatory of the convention, and on December 29, 1994, the Brazilian Federal Government established the National Programme of Biological Diversity (PRONABIO) [267], [268]. This program has been modified since that time to coordinate implementation of Brazil's commitments to the convention, and the Brazilian Ministry of Environment has played a key role in this process, which includes the formulation of the National Biodiversity Policy (Política Nacional de Biodiversidade, PNB). The PNB was prepared in consultation with the federal and states' governmental officials, nongovernmental organizations, scientific, indigenous and local communities, and entrepeneurs. As part of this process, the ministry has coordinated a series of baseline studies, such as an evaluation of the adequacy of the Brazilian legislation in relation to the Convention on Biological Diversity, a state-of-the-art synthesis of the knowledge of the Brazilian biodiversity, a comparative analysis of national biodiversity strategies from 46 countries, and a synthesis of records of traditional knowledge associated with biodiversity [275]. Also, parallel to the national consultancy, the ministry has promoted a general evaluation of seven major biomes in Brazil, including that on the coastal zone and marine environment [267], [268]. Currently, despite existing policies, there is an intensification of conflict between small-scale and industrial fishermen, shrimp farming and mangrove crab harvesting, resorts installation and native communities, NGOs and activities of oil and gas companies, and between federal and state governmental agencies in Brazil over environmental permits [270]. The major challenge for PRONABIO has been to demonstrate the direct benefits of conserving biodiversity and to promote the public action required to increase and guarantee the sustainable use of biodiversity.

**Figure 6:**
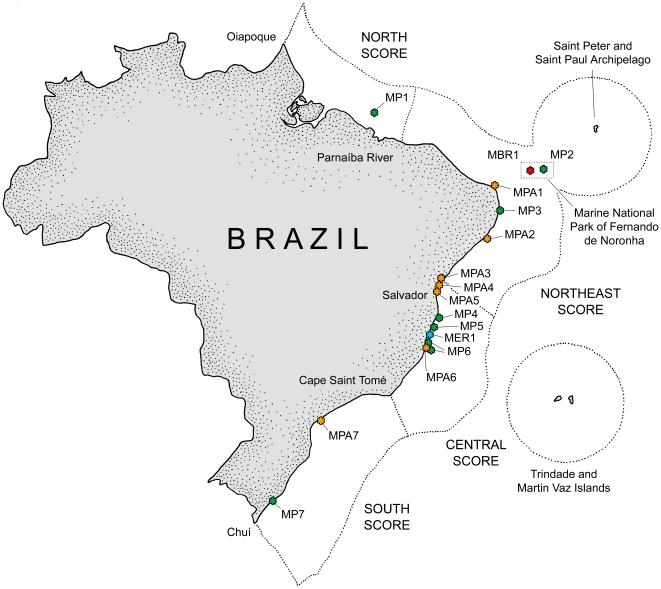
Map of the Marine Protected Areas (MPAs) of Brazil.

Even though Brazil has implemented conservation practices in coastal and maritime zones (Marine Protected Areas, Marine Reserves, and Marine National Parks), these efforts represent less than 0.4% of the total area within the territorial sea and EEZ (Figure 6) [269]. Several initiatives have been put in place to change the way people think. These initiatives include teaching the concept of conservation units through the demonstration of case studies, implementation of participative shared management of resources, capacity building aimed at technicians and managers, and outreach to decision makers [276]. Some of these coastal and marine conservation units have been set in the northern coast of Paraná and south of São Paulo, as well as in the south of Bahía, Rio de Janeiro, and Santa Catarina [276]. Today Brazil has 16 Marine Protected Areas mostly over coral reef ecosystems, including three recognized by international acts (RAMSAR and Natural World Heritage sites) [276].

Shallow-water reefs (those occurring on the continental shelf), are an important physiographic feature of the coast of Brazil and occur along at least one-third of the coastline (about 3,000 km, from Maranhão to south of Bahia). Coral reefs prevail northward (0°52′N to 19°S) and rocky reefs southward (20° to 28°S) [170], [248], [277], [278]. These extensive areas encompass diverse reef fish and invertebrate communities, in many places overexploited, where only recently have studies related to the impacts of fisheries on these ecosystems provided the basis for implementing management and conservation actions ([276]–[280] and authors therein). Around 18 million people depend directly or indirectly on reef ecosystems in Brazil [249]. As coral reefs are recognized as areas within the Convention, several actions with regard to these environments have been motivated in Brazil. The “Atlas dos Recifes de Coral nas Unidades de Conservação Brasileiras” (Atlas of the Coral Reefs within the Brazilian Conservation Units) published in 2003 was the first initiative to map the corals in the South Atlantic, not included in world maps before. There is a campaign for the Conscious Conduct in Reef Environments, outreach activity on conservation aimed at tourists. A monitoring program of Brazilian coral reefs (Reef Check Brazil, http://reefcheck.org) aims to establish the baselines for the conservation units national monitoring program that protect these ecosystems (this has now more than five years of sampling data). The Ministry has established partnerships with projects such as the Coral Vivo Project (Live Coral, www.coralvivo.org.br) in which several techniques for coral reproduction have been used, besides the country's enrollment in the International Coral Reef Initiative. Other projects associated with reefs are worth mentioning. The Institute Chico Mendes of Biodiversity Conservation (ICMBio – http://www.icmbio.gov.br), an organization responsible for conservation and management of threatened species in Brazil, is leading a national initiative to assess the status of conservation of species, including coral reef species, in partnership with IUCN and the Global Marine Species Assessment. The Goliath Grouper Project (http://merosdobrasil.org) benefits the goliath grouper *Epinephelus itajara*, the largest Atlantic grouper, which is considered a critically endangered species according to IUCN criteria and has been protected by the Brazilian Federal Law since 2002. The Marine Management Areas Science Program is an international program of Conservation International that is evaluating the effects of different management regimes to devise the best actions for the future. Within this context, the Abrolhos Shelf is part of a network attempting a similar experiment in parallel, which includes four intensive study areas around the globe (Brazil, Fiji, Belize, and Panama). Also in Abrolhos, the mesophotic reefs, holding unique “twilight zone” assemblages, have been revealed through a multidisciplinary and multi-institutional project in which remotely operated vehicles have been used unveiling the potential of the area for a variety of ecosystem services.

The established Brazilian Marine Protected Areas, Marine Reserves, and Marine Parks are fairly recent, the majority implemented with the intention to conserve biodiversity and sustain the natural habitats of marine organisms from all realms [167], [168], [276]. The Marine State Park Parcel Manoel Luis, for instance, includes three coral banks off the northern coast of Maranhão State, at the northern distribution limit of several fish species that are endemic to the Brazilian coast [167], [168]. Also, a complex estuarine system of islands, bays, coves, and mangrove forests make up the Reentrancias Maranhenses in the same state and is designated as a RAMSAR site (http://www.mma.gov.br) because of its great importance for numerous species of fish, shellfish, migratory birds, and manatees [167], [168]. Other examples include Atol das Rocas and Fernando de Noronha Marine National Park, both off the northeastern coast. Apart from being a Marine Reserve, Atol das Rocas is also considered a Natural World Heritage Site. It is the second largest reproductive area for the sea turtle *Chelonia mydas* and the main reproductive area for the seabird species *Sterna fuscata*, *Sula dactylatra*, *Sula leucogaster*, *Anous stolidus*, and *Anous minutus*. In the southern coast, the Arvoredo Biological Marine Reserve (Reserva Biológica Marinha do Arvoredo, RBMA) (27°17′7″S and 48°25′30″W) is an important nursery for many fish and other marine invertebrates [281]. All these and other conservation units have also been seen as a way of managing fisheries, especially where multispecific techniques are used and conventional management tools do not have any effect [276]. But several specialists have been pointing out the need for the establishment of no-fishing zones, including in the deep sea, as mechanisms for recovery and conservation of fish stocks [272]–[274].

Mangrove ecosystems cover 16 of the 17 Brazilian coastal states, representing 85% of the coastline (about 7,300 km), and are therefore crucial to local communities but also subject to huge pressures and human impacts. Mangrove ecosystems are among the most productive and have been considered essential to a variety of natural resources and environmental services, as they support economic activities and secure the environmental integrity in tropical coastal areas. In recognition of the importance of these ecosystems, the challenges of consolidatoffing and maintaining Mangrove Conservation Units, the Ministry of Environment, in partnership with the Instituto Brasileiro do Meio Ambiente e dos Recursos Naturais Renováveis – IBAMA (Brazilian Renewable Natural Resources and Environmental Institute) and the United Nations Development Program (UNDP), has submitted a proposal to the Global Environment Facility called “Project on the Conservation and Effective Sustainable Usage of Brazilian Mangroves” (known as Projeto GEF Mangue). This project is to raise funds to establish a network of protected areas that would allow the conservation and sustainable use of this country's 13,400 km^2^ of mangroves (equivalent to 9% of the total mangrove area worldwide) (http://www.mma.gov.br).

Apart from these economically important ecosystems, marine mammals, seabirds, and reptiles (mainly turtles) also receive special attention from NGOs and environmental agencies in Brazil. Projeto TAMAR-IBAMA (National Sea Turtle Conservation Program of Brazil), for instance, has a successful history of conservation with a joint governmental and nongovernmental administration, where local communities are involved [282]. Turtles have long lives and grow slowly to adulthood over 20 to 50 years. They have complex life cycles and use a variety of ecosystems, including the land where they lay their eggs as well as coastal and oceanic waters where they feed, develop, and mate [282], [283]. Five species of turtles occur in the Brazilian coast: *Caretta caretta*, *Chelonia myda*s, *Eretmochelys imbricata*, *Lepidochelys olivacea*, and *Dermochelys coriacea* [282]. Former egg poachers have been employed through the TAMAR Project to patrol the beaches and protect the nests, and this together with an education program and ecotourism have promoted the conservation of endangered sea turtles. Additionally, the project contributes to community festivals, supports local schools and health care facilities, and assists in developing alternative sources of income for residents who once had relied only on the exploitation of sea turtles [282]. The project has established 18 conservation stations covering 1,100 km of the Brazilian mainland coast. Like birds, however, turtles face other threats such as plastic debris and hook-and-line fishing bycatch [284]–[286], and there is a need for further monitoring and to develop mitigation measures [285].

Generally, Brazil is considered relatively poor in seabirds as a result of the low productivity of its tropical waters [287]. But about 130 coastal and marine species can be found throughout the coast and oceanic islands [288] . The great majority of these birds come from the Northern Hemisphere between September and May, and from the meridional extreme between May and August [288] [283], to mate and reproduce in marine protected areas such as the Atol das Rocas, are crucial for the maintenance of these populations.

Cetaceans are commonly sighted in along the Brazilian coast, and most studies have been related to their occurrence [289]–[297], abundance and distribution [252], [253], [298], diversity [204], [299], ecology [251], [300], [301], behavior and reproductive biology [246], [302], stranding [303], [304] and accidental capture [305]–[307]. Parente et al. [299] have evaluated the relationship between seismic surveys, oceanographic data, and diversity of cetaceans in Brazil since the increase in seismic survey activities. This study suggests that there is a decrease in the diversity of species over time, uncorrelated with changes in oceanographic patterns, but rather associated with the increasing number of seismic surveys. Nonetheless the authors recognize the need for further observations and improved methodologies to analyze the cetaceans' behavioral patterns. Apart from cetaceans, other mammals occur along the Brazilian coast and deserve protection, including manatees that are commonly found in mangrove areas in the North and Northeast regions and and fur seals that occur in the southern part of the country near Chuí. Manatees (*Trichechus manatus*) were hunted in the past for their meat and skin and were at risk of extinction, but they are currently protected by the Brazilian government. A dedicated center for the study and protection of manatees (Centro Nacional de Pesquisa, Conservação e Manejo de Mamíferos Aquáticos or Centro Mamíferos Aquáticos/IBAMA) was created in 1980. At that time, an extensive survey was carried out, areas of protection were established, and regional executive bases were implemented especially in the North and Northeast regions. This way, the animals have been rehabilitated; some reproduce in captivity and their young are maintained until they are ready for reintroduction to their natural environment.

There are only two refuges for pinnipids along the whole Brazilian coastline, and these are in Rio Grande do Sul state in the south. The South American sea lion (*Otaria flavescens*) is the most anthropogenically affected species, mainly because of its fishing interactions [214], [308] and other authors therein). A program for the conservation and management of pinnipids in Brazil (Programa de Conservação e Manejo dos Pinípedes – NEMA/IBAMA) was implemented from 1993 to 2004 for the protection of pinniped species that use the Rio Grande do Sul state seashore, and two conservation units exist in the south, but further efforts are necessary to promote environmental education, monitoring, and appropriate handling of these animals [214].

Ferreira et al. [309] have compiled information on the threat of invasive species for Brazil. They have considered that Brazil is undoubtedly a major receptor and donor of tropical and subtropical organisms in the world's oceans, taking into account the enormous variety of its marine ecosystems and the extent of its coastline. Currently, 66 invasive species have been recorded for the marine environment in Brazil from the following groups: phytoplankton (3); macroalgae (10), zooplankton (10), zoobenthos (38), fish (4), and pelagic bacteria (1) [310]–[312].

A trend toward increasing bioinvasion events in regional coastal ecosystems may exist, but data are still sparse and locally produced [309]. According to these authors, there might be a bias in actual invasion rates as a result of different research efforts in the recent past. As this is a relatively new topic in Brazil, the first comprehensive lists of introduced and invasive species are just beginning to be compiled, and the patterns of invasion are not well understood [309], [313].

### Subregion 5: The Tropical West Atlantic - Venezuelan Atlantic, Guyana, Suriname, and French Guiana

The Tropical West Atlantic region is bounded by the non-Caribbean section of the coast of Venezuela as well as by Guyana (formerly British Guyana), Suriname, and French Guiana, and defined by Longhurst [314] as the “Guianas Coastal Province.” It extends for about 1,877 km along the Atlantic coast of South America from the Brazilian border with French Guiana (4°20′20″S, 51°22′12″W) to the northern section of the Venezuelan Atlantic (10°39′22″N, 61°39′52″W). In the northern sector, the deltaic plains of the Orinoco and the Gulf of Paria in the north Atlantic coast of South America cover 2,763,000 ha and constitute one of the major wetlands in South America as well as one of the best preserved ecosystems in the world. The productivity of this area is significant and one of the highest among neighboring areas in the adjacent Caribbean [315] (Figure 4). These wetlands were formed by the combined action of sediment and freshwater discharges from the Orinoco, one of the longest rivers in South America (2,140 km) along with the tides on a flat alluvial plain [316]. The physical and chemical characteristics as well as the ecosystems that develop in this area are therefore defined by these factors [317]. The surface sea temperature is relatively constant throughout the year (27°C–28°C), and temperature drops to 12°C at 200 m depth (Figure 3). During the dry season, salinity at the Gulf of Paria is about 35–35‰, while during the rainy season it may drop to 10‰ with variations in the vertical gradient corresponding to an estuarine environment. Predominant winds in this area are the northeast trade winds, with a mean speed of 6.6 m/s in the Atlantic Front and 2 m/s in the Gulf of Paria. Winds show a seasonal pattern in which the highest speeds are observed in January, February, and March (monthly mean: 7.5 m/s), and the lowest in July, August, and September (monthly mean: 5.7 m/s). In most of the continental portion of Venezuela and many coastal areas, wind intensity is also associated with cumulonimbus cloud systems, which are often observed during the rainy season. The Venezuelan coast is not often affected by hurricanes or tropical storms. However, these events can occur, and hurricanes have at times reached the Venezuelan coast at a frequency of one every 36 years. In these cases, wind speeds have increased to almost 40 m/s. Wave pattern is also mostly determined by the northeast trade winds, although this pattern may be altered by changes in wind intensity and by extratropical cyclonic depressions that occur in the North Atlantic, generating waves that reach the Venezuelan coasts as swells. Waves are usually 1–6.25 m in height and frequently more than 4 m in May, November, and December. Offshore the Orinoco Delta, currents are dominated by the Guayana Current, which flows mainly toward the northwest at about 150 cm/s, significantly affecting the entire region because of the large amounts of water it transports (Figure 2). On the other hand, the Orinoco River discharges also affect the circulation pattern of the oceanic water mass seasonally throughout the year (rainy and dry seasons). The Orinoco has the world's third-largest flow (average discharge of 5.4×10^11^ m^3^/year), which, combined with that of the Amazon River, accounts for 25% of all the freshwater discharged to the world's oceans. Tides are usually semidiurnal and vary from 1.7 to 4.5 m depending on the zone [318].

In the southern sector of this region, the climate in French Guiana is typically wet equatorial, driven by the Intertropical Convergence Zone. Rainy season is mainly between May and June, but there is a secondary rainy season in January and February. Both periods greatly influence the Amazon River discharge, making the waters extremely turbid. Tides are semidiurnal with an amplitude of up to 2.5 m. The main currents are the North Brazil Current becoming the Guianas Current, which flows to the northwest and carries low-salinity waters rich in nutrients and sediment from the Amazon (Figure 2). Upwelling is also characteristic of this sector, providing more nutrients to the water but not decreasing significantly its temperature [18] (Figures 3 and 4).

From an ecological point of view, the coastal marine habitats in the northern sector of this region can be divided into several subareas: (1) the coastal fringe south of the Paria Peninsula, dominated by rocky shores, (2) the coastal fringe of the Gulf of Paria and the Atlantic Ocean, dominated by mangroves, and (3) the Atlantic coasts, dominated by soft bottoms and sandy beaches. All of these are part of the “Gulf of Paria and Atlantic Front” ecoregion as defined by Miloslavich et al. [319]. Each of these subareas has ecologically distinct features that are determined by the particular physiography, hydrodynamism, tides, sediments, physics, and chemistry of the area. These conditions allow the development of distinct ecosystems along this “variably stable” continental fringe that are characterized by a total interdependency between biotic and abiotic components [315]. In the southern sector, the coastal habitats are mainly mudflats, extensive mangrove swamps, narrow sandy beaches, and brackish water creeks [18].

#### Marine biodiversity in the Tropical West Atlantic

A total of 2,743 species have been reported in this region (Tables 6 and S9). The most diverse groups were the fish (32%), followed by the crustaceans (19%), the mollusks (16%), and the polychaetes (6%). Despite having a large coastal extension, neither the Gulf of Paria nor the Venezuelan Atlantic Front including the Orinoco Delta has been well studied. Knowledge of the marine biodiversity of the area is scarce and mostly reported in gray literature. The first studies of benthic communities in the Gulf of Paria and the Venezuelan Atlantic Front were carried out in the 1960s and 1970s, mostly focused on crustaceans [320], gastropods [321]–[328]. In the late 1990s and early 2000s, baseline studies were carried out in the area in response to the interest of oil and gas companies in establishing both offshore and coastal developments. Such studies produced some species lists, but because of the lack of taxonomic expertise, these are incomplete and do not reflect well the actual biodiversity [316], [329]. Recently, more extensive biodiversity and environmental impact studies have been developed [316], [318], [330], [331] and a complete environmental baseline is compiled in Martín et al. [329].

**Table 6:** Summary of the diversity, state of knowledge, and expertise of the main taxonomic groups within the Tropical West Atlantic subregion of South America.

Taxonomic group	No. species^1^	State of knowledge	No. introduced species	No. experts	No. ID guides^2^
**Domain Archaea**		1	0		
**Domain Bacteria (including Cyanobacteria)**		1	0		
**Domain Eukarya**					
**Kingdom Chromista**					
Phaeophyta	12	3	0	2	
**Kingdom Plantae**					
Chlorophyta	24	3	0	2	
Rhodophyta	98	3	3	2	
Angiospermae	7	4	0	2	
**Kingdom Protista (Protozoa)**					
Dinomastigota (Dinoflagellata)		1	0		
Foraminifera	48	2	0	1	
**Kingdom Animalia**					
Porifera	23	2	0	1	
Cnidaria	131	2	0	1	
Platyhelminthes		1	0		
Mollusca	431	3	3	3	
Annelida	172	3	1	2	
Crustacea	519	3	1	12	23
Bryozoa		1	0		
Echinodermata	107	3	0	2	
Urochordata (Tunicata)	16	2	0	1	
Other invertebrates	43	2	0		
Vertebrata (Pisces)	889	4	2	2	2
Other vertebrates	223	4	1	4	1
**SUBTOTAL**	2743		11		
**TOTAL REGIONAL DIVERSITY** 3	2743		11		

1 Sources of the reports: databases, scientific literature, books, field guides, technical reports.

2 Identification guides cited in References.

3 Total regional diversity, including all taxonomic groups as reported in Table S9.

The OBIS database currently lists 2,095 species in the Tropical West Atlantic, which represents 76% of the total as updated in this paper (Table 2). Even though most of these species are not new descriptions, a significant number of them were not reported in this area until recently, particularly in the Venezuelan Atlantic Front area. In this particular area, of the 1,561 species that have their collection date registered in OBIS (since 1884), 50% were collected between 2001 and 2004, and 47% between 1950 and 1980. In general, the best-known taxonomic groups are fish and crustaceans, both important as fisheries resources, which account for about 51% of the total known biodiversity. The mollusks, for example, usually the most diverse group, account for only about 15% of total biodiversity, and the other major groups such as macroalgae, sponges, cnidarians, and polychaetes account for less than 20% of the total.

The most recent review of decapod crustaceans of the lower Orinoco Delta reports 30 species (23 genera and 12 families), of which the most abundant were the shrimps *Litopenaeus schmitti*, *Macrobrachium amazonicum*, and *Xiphopenaeus kroyeri* [332]. In the Gulf of Paria, about 300 species have been reported, and of these, the gastropods are the most diverse group (200 species), followed by the crustaceans (22 species) and polychaetes (11 species) [333]. In the Atlantic Front, sampling between 2001 and 2002, collected macrofauna of 11 phyla: Protozoa, Porifera, Cnidaria, Nematoda, Nemertea, Annelida, Sipuncula, Echiura, Mollusca, Crustacea, and Echinodermata. Of these, annelids (mainly of the families Pilargidae, Spionidae, and Paraonidae) were the most abundant group, representing 60.7% of total abundance, followed by crustaceans (mainly peracarids) and bivalves with 15.4% and 9.3%, respectively, The most diverse polychaete famlies were Onuphidae and Syllidae, followed by Paraonidae. The shallow zone (less than 200 m) had higher abundances than the deeper zones for all groups [330]. Other important groups are the peracarid crustaceans, which were collected in 42% of the samples, amongst which the amphipods were the most abundant group (57.8%), followed by the isopods (20.7%), cumaceans (12.1%), and tanaidaceans (9.5%). Sampling was carried out up to 200 m in depth and higher abundances were found in the shallower zone, above 200 m (86%) [334]. Bone et al. [335] reviewed the taxonomic composition of the Orinoco Delta benthic community and reported a total biodiversity of 31 species belonging to four phyla (Nematoda, Annelida, Mollusca, and Arthropoda), one subphyllum (Crustacea), four classes (Polychaeta, Gasteropoda, Maxilopoda, and Malacostraca), two subclasses (Ostracoda and Copepoda), one suborder (Peracarida), two orders (Decapoda and Mysidacea), and 22 families.

Few studies of the planktonic community have been made. A total of 367 species of marine and estuarine phytoplankton and 182 species of zooplankton have been reported for the Orinoco Delta and its zone of influence in the Atlantic Ocean. These communities are strongly influenced by rain and tidal regimes [335]–[341]. The nektonic community is also affected by rain seasonality, both in biodiversity and in biomass. During the rainy season, fish diversity and biomass (29,318 t) are higher and dominated by estuarine species. During the dry season, both fish diversity and biomass (10,611 t) are lower and dominated by marine species. This region has a great potential for future research and species discovery. Few taxonomic groups are well known, while most of the groups are either poorly known or almost unknown.

#### Threats and conservation strategies in the Tropical West Atlantic

The Tropical West Atlantic is heavily fished by local populations, and many species, primarily fish and decapod crustaceans, have commercial value. For some of these species, there is information about their biology (reproduction, fecundity), ecology and fisheries [342]–[359]. The impact of such fisheries on biodiversity is poorly known. Fisheries focus on catching shrimp, scienid fish, and catfish, which are abundant in estuarine habitats, and snappers and groupers, abundant in deeper waters and on rocky bottoms. Historical data on industrial trawling fisheries have shown six species of catfish, scienids, carangids, and lutjanids (snappers). The most important species for longline artisanal fisheries have been the red snapper (*Lutjanus purpureus*), the grouper *Epinephelus flavolimbatus*, and the snapper *Rhomboplites aurorubens*. The most important species captured with lines are the “carite sierra” (*Scomberomorus cavalla*), the barracuda (*Sphyraena barracuda*), the “dorado” (*Coryphaena hippurus*), and the “peto” (*Acanthocybium solandri*) [360].

Major threats to biodiversity in this region are industrial (trawling) and artisanal (line and longline) fishing, urban development, agriculture development, dredging and flow navigation, water pollution (runoff from the Orinoco and Amazon basins), mangrove deforestation, activities related to oil and gas exploitation, port activities, and maritime shipping [331]. These authors assigned values to each of these threats according to their level of menace on a scale from 1 to 8 (from least to highest impact). By this measure, the most threatening activities are those related to oil and gas exploitation, industrial fisheries, dredging, and mangrove deforestation. In regard to industrial fishing, a new Fisheries and Aquaculture Law (article 23) has prohibited industrial shrimp trawl activities within Venezuela's ocean territory and exclusive maritime economic zone, starting on March 14, 2009. It is expected that the impact of this activity will cease to be a problem in the near future at least within Venezuelan waters. The impact of oil- and gas-related activities depends in great measure on whether these activities are offshore or at the coastline. The impact of offshore activities, when carried out within strict safety parameters, are usually limited to the area surrounding the platforms. This cannot be said of activities on the coast, where the impact is much greater and is spread over a much larger area. Environmental catastrophes such as the British Petroleum Deepwater Horizon in the Gulf of Mexico, despite being extremely atypical, dramatically alert on the risks of carrying out such environmentally risky activities in off shore areas without the proper security measures.

The Tropical West Atlantic region includes several MPAs within the different countries covering nearly 10,900 km^2^ overall (land and sea). In Venezuela, the Orinoco Delta and Gulf of Paria region have two protected areas under special conservation regulations. These are the Turuépano National Park in the Gulf of Paria, and the Orinoco Delta National Park. Of these, the most impressive is the Orinoco Delta National Park, which is also a Biosphere Reserve of mainly land and estuarine areas [319]. Recently, Klein et al. [331] engaged in a conservation study in this area carried out by the Universidad Simón Bolívar and the Nature Conservancy to suggest and establish, based on conservation objects, marine areas to be declared under protection. The conservation objects chosen for this area were the rocky shores, the sandy beaches, and the soft bottoms. One of the recommendations given by these authors for conservation is to expand the Orinoco Delta National Park farther into the oceanic area to protect the marine environments as well. In Guyana, there are no formally established MPAs, but the 140 km long “Shell Beach,” a nesting site for at least four species of marine turtles, is protected directly and indirectly by conservation activities involving local communities. In Suriname, there are seven MPAs, of which four are Nature Reserves and two are multiple-use management areas. In French Guiana, there is only one Nature Reserve of about 78 km^2^ of marine areas.

#### Microorganisms in South America: Bacteria and Phytoplankton

The best-known marine phytoplankton taxonomic groups are diatoms and dinoflagellates. As an example, in Mexican marine waters, the number of taxa recorded is about 1,400 [361]. Recent studies on phytoplankton dynamics complete this picture in South American estuarine systems, including those of Gómez et al. [362], Calliari et al. [363], Licursi et al. [364], and Carreto et al. [365] in the Río de la Plata and of Popovich and Marcovecchio [366] in the Bahia Blanca estuary, as well as in littoral tropical systems [367]. On the other hand, phytoplankton studies, together with food web and biogeochemical flux estimations, have intensively been carried out in the upwelling system off Chile [368]–[372] and in southern Chilean fjords [373]. Phyto- and bacterioplankton dynamics are also studied in French Guiana coastal and shelf systems under direct Amazon influence [43], as well as in subtropical lagoons in southern Brazil, focusing in phytoplankton dynamics and trophic fate [374], [375], and in South Atlantic oceanographic frontal systems [376]–[378]. The diversity of picoeukarya and cyanobacteria was investigated at intermediate shelf stations in the Patagonian system [43] [40]. Microbial dynamics (Eukarya and Eubacteria) are intensively explored in central Chile [379]–[383] and in the Peruvian upwelling system [384], related to the oxygen minimum zone and big upwelling productivity and remineralization patterns. Biogeographical issues are also considered in a recent survey on bacterial assemblages (phylum level) in surface waters from the Gulf of Mexico to the southeastern tropical Pacific [385]. Bacterial dynamics and diversity are studied in coastal lagoons in Uruguay [386], in sediments of fluid mud in French Guiana [387], in waters and sediments of the oxygen minimum zone off the South American Pacific coast [388], and in anoxic waters of the Cariaco Basin ([389], Chistoserdov et al., upublished), where novel Eukarya are also studied [390].

In polluted coastal systems, bacteria with ability to degrade pesticides and hydrocarbons are currently monitored. In coastal areas of the Colombian Caribbean, 64 native marine bacterial strains were isolated from sediment samples [391]. The oil-degrading bacteria are also studied in the Orinoco Delta, which has been subject to intensive oil exploitation. Furthermore, the Microbial Observatory of Rio de Janeiro (MoRio) [392], [393] established in Guanabara Bay (Brazil), by exploring microbial biodiversity in different coastal systems (including unpolluted sites) constitutes a model for the study of threatened tropical coastal systems. The activity and diversity of hydrocarbon- and oil-degrading bacteria are assessed also in temperate waters and sediments of coastal systems of Argentina [394], [395]; Dionisi et al., unpublished). Finally, symbionts and pathogenic microbes are currently assessed in coral reefs of the Caribbean and South America [396], as well as in mangroves [397] and extreme environments [398].

## Discussion

### Analysis of latitudinal trends in biodiversity and species richness

The regional analysis of South American marine biodiversity showed tremendous heterogeneity not only in physical environments, including size and conditions, but also in research capacity, history of exploration, and conservation actions. Threats to biodiversity seem to be more or less common to all the subregions, varying probably in the level of intensity from one subregion to another. South American marine biodiversity is least well known in the Tropical East Pacific (with the exception of Costa Rica and Panama) and the Tropical West Atlantic, although the latter subregion has a slightly higher diversity when the total number of species is standardized by coastal length—nearly 150 species in 100 km of coast (Table 7). In the Tropical West Atlantic, particularly in the Venezuelan Atlantic Front, sampling of marine biodiversity has intensified in recent years [316], [332], [333], [335], [339], [340], significantly increasing our knowledge, but there are still many gaps and unknowns. One of the major limits to the knowledge of marine biodiversity in this region is the shortage of taxonomic expertise. As reported in Table 6, there are 2,743 species known to this region, of which 2,475 (90.2%) are from only five major groups: fish and other vertebrates (birds being highly diverse), crustaceans, mollusks, polychaetes, echinoderms, and macroalgae. This means that overall diversity is probably highly underestimated, especially in less-known taxonomic groups.

**Table 7:** Number of species of cnidarians, mollusks, crustaceans, echinoderms, and fish per kilometer of coast and per South American subregion.

Subregion	Taxonomic group	Number of species by taxonomic group	% of total species	Species/100 km of coast
**Tropical East Pacific**	Fish	1212	18.1	23.8
Coastal length: 5100 km	Crustaceans	863	12.9	16.9
Total species: 6714	Mollusks	875	13.0	17.2
	Echinoderms	223	3.3	4.4
	Cnidarians	110	1.6	2.2
	**TOTAL**	**3283**	**48.9**	
**Humboldt Current system**	Fish	1167	11.4	16.0
Coastal length: 7280 km	Crustaceans	3136	30.7	43.1
Total species: 10201	Mollusks	1203	11.8	16.5
	Echinoderms	364	3.6	5.0
	Cnidarians	517	5.1	7.1
	**TOTAL**	**6387**	**62.6**	
**Patagonian Shelf**	Fish	539	14.3	9.5
Coastal length: 5649 km	Crustaceans	611	16.2	10.8
Total species: 3776	Mollusks	849	22.5	15.0
	Echinoderms	207	5.5	3.7
	Cnidarians	258	6.8	4.6
	**TOTAL**	**2464**	**65.3**	
**Brazilian Shelf**	Fish	1294	14.2	17.3
Coastal length: 7491 km	Crustaceans	1966	21.6	26.2
Total species: 9103	Mollusks	1833	20.1	24.5
	Echinoderms	254	2.8	3.4
	Cnidarians	535	5.9	7.1
	**TOTAL**	**5882**	**64.6**	
**Tropical West Atlantic**	Fish	889	32.4	47.4
Coastal length: 1877 km	Crustaceans	519	18.9	27.7
Total species: 2743	Mollusks	431	15.7	23.0
	Echinoderms	107	3.9	5.7
	Cnidarians	131	4.8	7.0
	**TOTAL**	**2077**	**75.7**	

From a biodiversity perspective, globally, coastal and shelf waters not only present the greatest species richness (but see Gray, [399]) and highest productivity [400] of the world's oceans, but they also are biogeographically distinct from the adjacent high seas and deep benthic environments [50], [401]. In the South American continent, deep-sea exploration is relatively recent, and most efforts have been concentrated in the southern countries, mainly Brazil (Table S7).

In general, the best-known taxonomic groups in the marine environments worldwide are the cnidarians, mollusks, crustaceans, and echinoderms among the invertebrates, and the fishes [402]. These groups together usually account for 50%–60% of the known marine biodiversity. In the global analysis carried out by the National and Regional Committees of the Census of Marine Life (see PLoS ONE collection “Marine Biodiversity and Biogeography – Regional Comparisons of Global Issues”: http://dx.doi.org/10.1371/issue.pcol.v02.i09), the crustaceans, molluscs, and fishes comprised approximately 50% of all known species across the 25 regions studied [403]. In the OBIS database, for instance, which is the largest marine biodiversity database in the world with nearly 25 million species distribution records, from over 100,000 different species and 750 datasets (by April 2010), these groups combined account for 69.7% of all species (9.0% cnidarians, 11.4% mollusks, 23.0% crustaceans, 5.3% echinoderms, and 21.1% fishes). In the South American subregions, these taxonomic groups account for 54.2% in the Tropical East Pacific, 62.6% in the Humboldt Current, 65.3% in the Patagonian Shelf, 64.6% in the Brazilian Shelves, and 75.7% in the Tropical West Atlantic (Table 7). The fact that their proportion in the Tropical East Pacific is much lower than expected indicates that even for these well-known groups, there is still much to discover.

Data show important differences in total biodiversity between the Atlantic and Pacific oceans at the same latitude. In this sense, as mentioned earlier, in the north of the continent, the Tropical East Pacific is richer in total number of species than the Tropical West Atlantic (a difference which is not so evident when standardized by kilometers of coast), and in the south, the Humboldt Current system is much richer than the Patagonian Shelf.

It has been proposed that in marine environments, biodiversity is greatest in tropical regions, decreasing gradually toward higher latitudes [404]–[407]. This trend has been observed at the regional level in mollusks and isopods [405]–[408], but not in the local patterns of intertidal macrobenthic fauna [409]. On the other hand, intertidal assemblages of echinoderms at the global level have been reported to peak in high northern latitudes and clearly decline with latitude, while subtidal assemblages of echinoderms show no latitudinal trends but rather seem to have regional diversity hotspots [410]. Empirical studies [411] and meta-analysis [412] have shown that this relationship between latitude and species richness is based on the decline of regional biodiversity (gamma biodiversity) toward the poles, and not on the variation of the local community richness (alpha biodiversity). Boltovskoy et al. [413] suggested that the trend toward decreasing biodiversity with increasing latitude seemed to be balanced by a higher biomass and endemism at higher latitudes. However, there has been little systematic effort to document these patterns in the southwestern Atlantic, and most existing efforts are almost exclusively focused on invertebrates [108], [414]–[416]. On the other hand, Gray [399], [417] reported that species richness in the Antarctic is high, questioning the validity of the proposed latitudinal pattern. To test whether this pattern is valid or not, it is necessary to review as much information as possible regarding local and regional species richness [9]. In this sense, the above mentioned global analysis [403], showed that the most diverse coastal areas in the world are within Japanese and Australian waters (about 33,000 species each) followed by Chinese waters (about 22,000 species). A recent analysis carried out with about 11,500 species across 13 separate taxonomic groups of coastal and oceanic environments, showed that there are different diversity patterns for coastal and oceanic species, with coastal species being more diverse in the equatorial West Pacific, and the oceanic species being more diverse in mid latitudes. For all groups studied, sea surface temperature was identified as a significant driver for these patterns, while habitat availability was significant for most, however not all, of the groups [418].

**Figure 7:**
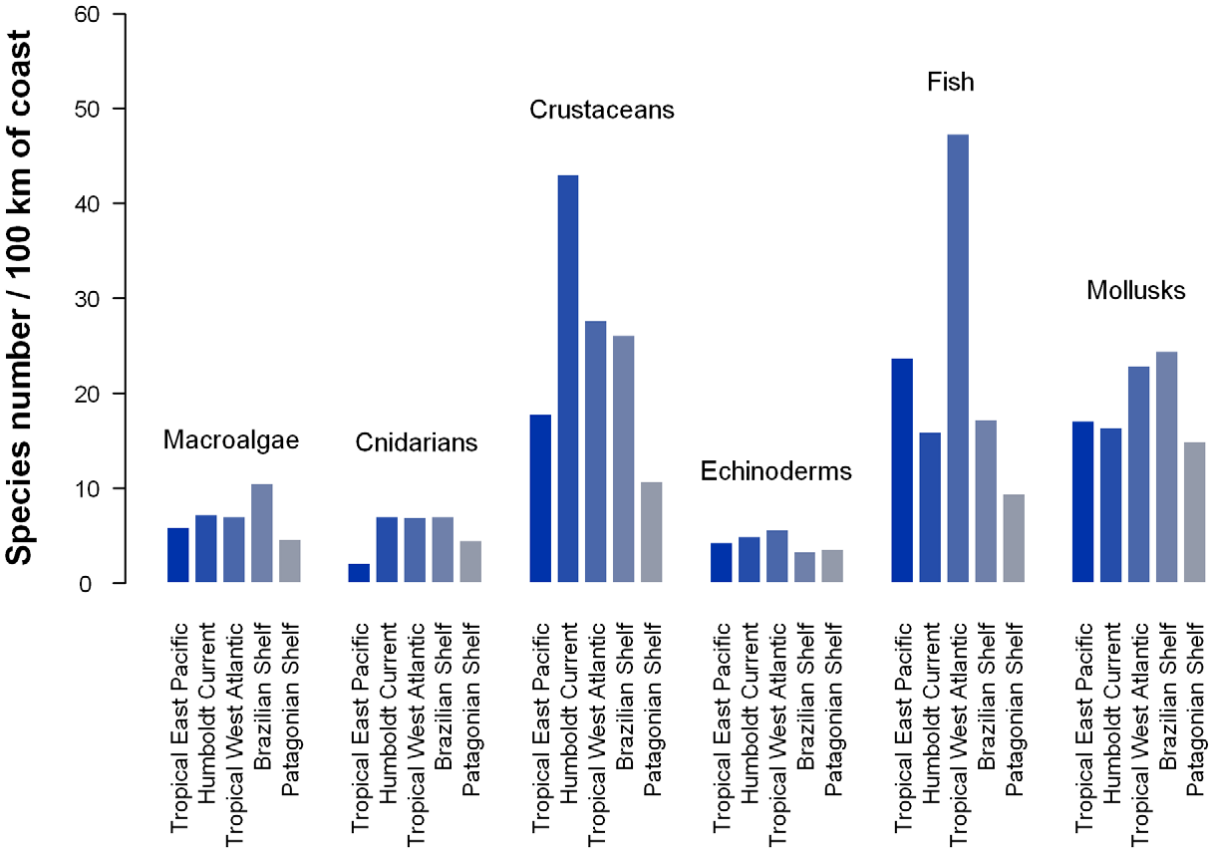
Number of species per 100 km of coast for the major taxonomic groups (macroalgae, cnidarians, mollusks, crustaceans, echinoderms, and fishes) for the five South American subregions studied.

In the north of the South American continent, the tropical Caribbean region, has about 12,000 marine species, a number which is certainly higher than for any of the subregions in this paper [20].The data reviewed here shows that for the Atlantic Ocean, the tropical region has higher biodiversity than the temperate region, varying from 146 species per 100 km of coast in the Tropical West Atlantic to 122 species per 100 km of coast in Brazil, and to 67 species per 100 km of coast in the Patagonian Shelf (Table 2). On the other hand, this trend is not evident in the Pacific Ocean, as the diversity in the Tropical East Pacific is 132 species per 100 km of coast and a little higher in the Humboldt Current system (140 species per 100 km of coast). When these comparisons are made within particular taxonomic groups, the latitudinal trends mentioned earlier for total biodiversity in the Atlantic Ocean can only be observed for fish and crustaceans (Figure 7). Regional “hot spots” of biodiversity for the best-known taxonomic groups seem to be in the Tropical West Atlantic for fishes, in the Humboldt Current for crustaceans, in Brazil and the Tropical West Atlantic for mollusks, and in Brazil for macroalgae.

There is not a clear relationship between increasing latitudes and increasing species richness for macroalgae, and it has been stated that temperate regions can achieve species numbers at least as high as those in the tropics [419]. In the northern hemisphere, latitudinal macroalgal trends in species density and biomass have been reported for some strata within the intertidal and shallow subtidal zones, with more taxa and biomass at higher latitudes [420]. In the southern hemisphere, the floras of the Patagonian coast, Tierra del Fuego, and Malvinas are recorded among the most species diverse in the Southern Ocean [421]. The data presented in this paper show that macroalgae are an important group for the species richness of all regions, varying from 4.9% to 8.7% of total species biodiversity. In regional trends, the highest biodiversity of macroalgal species was found in the Brazilian region (10.6 species per 100 km of coast), followed by the Humboldt Current system (7.3 species per 100 km of coast), the Tropical West Atlantic (7.1 species per 100 km of coast), and the Tropical East Pacific (6.0 species per 100 km of coast). The lowest diversity was found for the Patagonian Shelf (4.7 species per 100 km of coast), which could seem contradictory to the previous statement by John et al. [421], but this could be because the relatively small hot spots of macroalgal diversity found in the scarse rocky shores of the Patagonian Shelf are being “diluted” among hundreds of kilometers of sandy coasts with no macroalgae.

**Figure 8:**
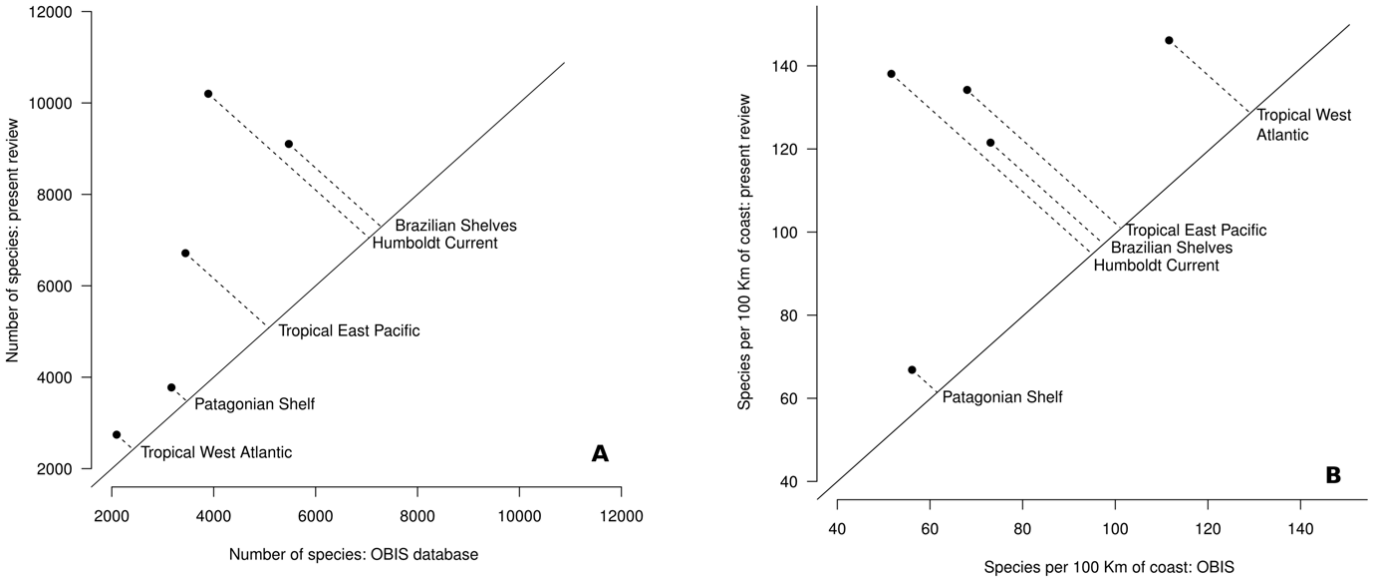
Number of species in the OBIS database versus the number of species in the present review. A: Total number of species. B: Species per 100 km of coast. The largest the length of the dashed line (deviation from the diagonal), the largest the difference between the two datasets (OBIS and the present review).

**Figure 9:**
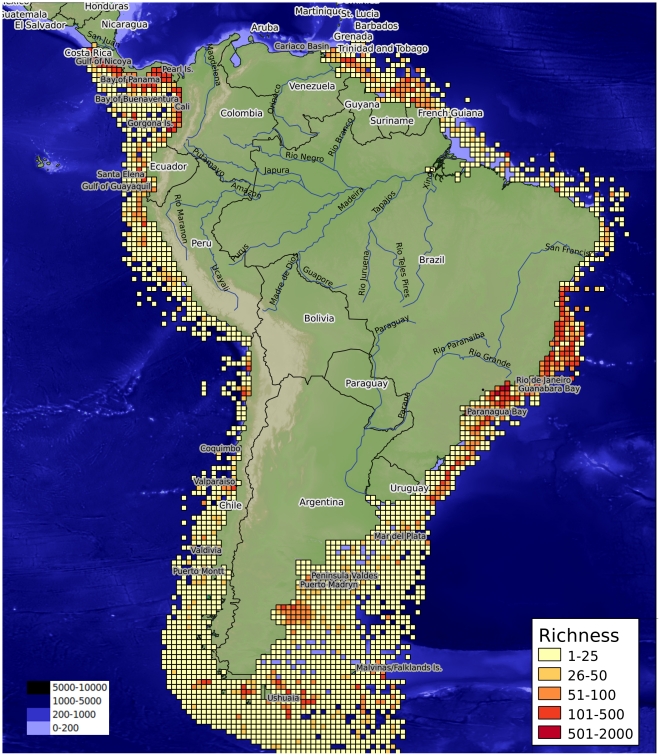
Map showing the distribution of marine biodiversity around the South American continent using data from the OBIS database. Richness scale represents number of species. Bathymetry scale in meters.

The trends discussed here, however, both for fauna and macroalgae, may not truly reflect real patterns, as sampling has not been equal throughout the continent, and taxonomic capacity is very uneven from one country to another as is the case in the Caribbean [20]. These patterns are based on analysis of a thoroughly updated biodiversity review as was carried out in each of the South American subregions in this paper. But the patterns cannot be visualized correctly because we do not know all the localities for all the species compiled here. To visualize marine diversity distribution patterns in South America, we relied in the OBIS database, which has more than 50% of the species for four of the subregions (between 51% and 84%), and about 38% for the Humboldt Current system (Table 2, Figure 8). From this figure it is evident that all regions as reviewed in this paper have a higher number of species than the number reported in OBIS (all dots above the diagonal line), and that the biodiversity in some regions is well represented in the OBIS database (e.g. Patagonian shelf) while in others, this is not the case (e.g. Humboldt current). Strictly with OBIS data, the patterns of biodiversity along the latitudinal gradient of the Atlantic Ocean are the same as those we report with updated data, but that was not the case for the Pacific Ocean, where the tropical zones show more diversity than the temperate zones (Figure 9). This difference is probably because the Humboldt Current system is poorly represented in the OBIS database. Based on this observed inconsistency, we tested for this particular region, which has the largest latitudinal variation in the continent, whether the expected pattern of biodiversity would have been different from the observed pattern given a homogeneous sampling effort. To test for this, we used the rarefaction technique to estimate the number of species that would have been recorded in a given number of observations (e.g., Magurran, [422]). In this analysis, we used a conservative number of 10 observations, which corresponds to the standardized sample size used to estimate the richness per cell using the rarefaction technique. An *a posteriori* neighborhood operation was conducted to improve the detection of biogeographical patterns. Using this function, we recalculated the values of each grid cell using the mean, according to the values of the cells in a 3×3 neighborhood around that cell. Later, the expected geographic pattern in biodiversity was compared with the observed biogeographic pattern from this study, and the provinces previously described for the southeastern Pacific coast by Camus [88].

**Figure 10:**
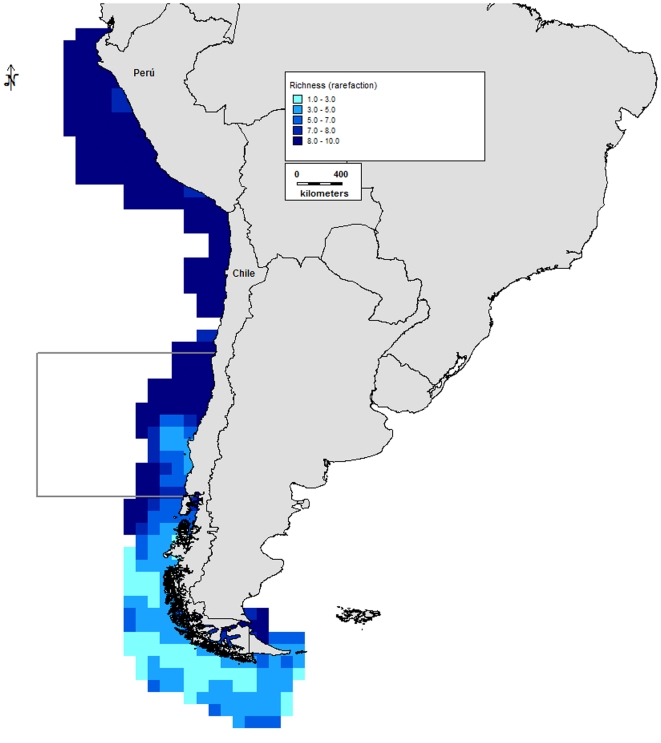
Expected species richness in the Humboldt Current subregion using the rarefaction technique to estimate the number of species that would have been observed given a standard number of 10 observations. Scale represents expected number of species.

The analysis of the distribution of patterns of richness along the Humboldt Current system observed in the OBIS database showed three zones of high richness (Figure 10) with the highest values found in the Strait of Magellan. This zone of maximum diversity is in accordance with previously described patterns of mollusk diversity on the southern Pacific coast [423], as well as with the observed pattern for marine invertebrates on the Chilean coast described by Lancellotti and Vásquez [424], [425] and polychaetes by Hernández et al. [89]. This zone of maximum diversity has historically experienced the combined effects of climatic processes, tectonic activity, and glaciers, provoking the formation of a large system of archipelagos, with an abundance of gulfs, fjords, and canals [88]. This zone has been associated with changes in local conditions (i.e., substrate types, tidal amplitude, temperature, and salinity) [426], which would generate a highly diversified mosaic of different biotopes [427], which would act as refuges during repeated glacial advances over the last 40 million years [428]. The sum of these factors would favor the local radiation of taxa, leading to the current area of high taxonomic diversity in the Strait of Magellan (52°–56°S) as reported in our study, and secondarily causing low faunistic affinity with taxa from the Antarctic Peninsula [429].

In the northern zone, the bands of lowest diversity (off southern Peru between 15°–19°S and northern Chile between 25°–29°S, Figure 5) are strongly influenced by the large-scale low-frequency spatial disturbances called El Niño/Southern Oscillation (ENSO). This phenomenon provokes a series of alterations in the structure of the current system and, consequently, the coastal biota of the region, with regional-scale influences up to 30°–36°S [430], [431]. Since the appearance of ENSO about 5,000 years ago [432], the southeastern Pacific biota has experienced a continued disturbing influence, and now ENSO is a critical component of regional dynamics, having played an important role in defining the current biogeography of the area [90]. According to Camus [430], the characteristics of ENSO probably subjected local populations to frequent bottlenecks and nonselective extinctions, which could generate high interpopulational variability and even provoke founder effects. These population-level processes, together with ENSO should have produced increases in local diversity; however, while our results do not support this hypothesis, they do support the ENSO hypothesis as a cause of extinctions and low diversity in the zone. The low diversity of benthic polychaetes observed in the northern zone can probably also be attributed to a low speciation rate, due to the low differentiation of niches (i.e., low diversity of microhabitats) observed in this zone with respect to the zone south of 41°S, which would function as a biological mechanism determining local-scale diversity [433]. Additionally, as was proposed by Moreno et al. [434], the northern latitude benthic richness of the HC potentially is controlled by the development of a shallow oxygen minimum zone during the Neogene [435]. This phenomenon, which is observed on the Peruvian and northern Chilean coasts, occurs at less than 50 m depth [436]–[438] and strongly influences the distribution and diversity of benthic marine species [439].

The rarefaction technique, used to evaluate the expected pattern of biodiversity, showed a consistent pattern of increase in the richness of marine species toward tropical latitudes (Figure 10). These results allow us to predict that a homogeneous sampling effort will improve the OBIS database and provide more accurate patterns of biodiversity. This expected pattern is a hypothetical scenario—constructed on a conservative number of 10 observations—that can only be evaluated if the OBIS database continues to grow, using new georeferenced data made available not only from new studies of marine biodiversity in the HC, but also by uploading in the OBIS system information that is already either in the literature or in local databases.

Research capacity is stronger in the southern countries of the continent, in Brazil, Argentina, and Chile, which also have a longer history in marine research. For example, contrary to what is generally stated abroad, the southwest Atlantic has had many oceanographic and biological studies for many years, but most past literature was mainly in Brazilian regional scientific journals in Portuguese. Many molecular tools have been used to study latitudinal gradients, identify cryptic and endemic species, and consider other questions related to biodiversity [440]–[444]. In the last seven years, a great effort has been made to incorporate data into open-access databases such as OBIS, especially from Brazil and Argentina through their OBIS nodes. However, there is still much information available locally that has to be incorporated into the system, as was demonstrated for the Humboldt Current system. On the other hand, it is true that even in the best-studied areas along the vast South American coastline, there is still much to be done and discovered, both in the continental shelf and especially in deep-sea environments.

#### Species discovery and analysis of endemism

**Figure 11:**
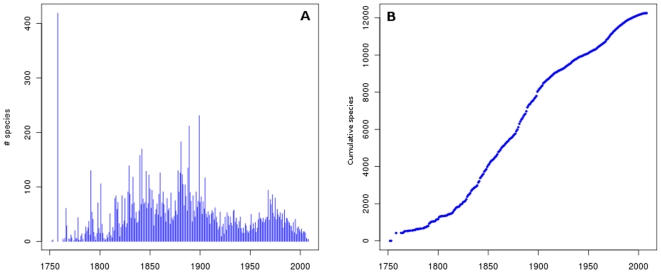
Species description in South America. A: Number of species described per year for all taxonomic groups. B: Species-description accumulation curves for marine species taking into account all taxonomic groups. Period: 1750–2000. Data from OBIS database (using only “valid names” which corrects for synonyms).

**Figure 12:**
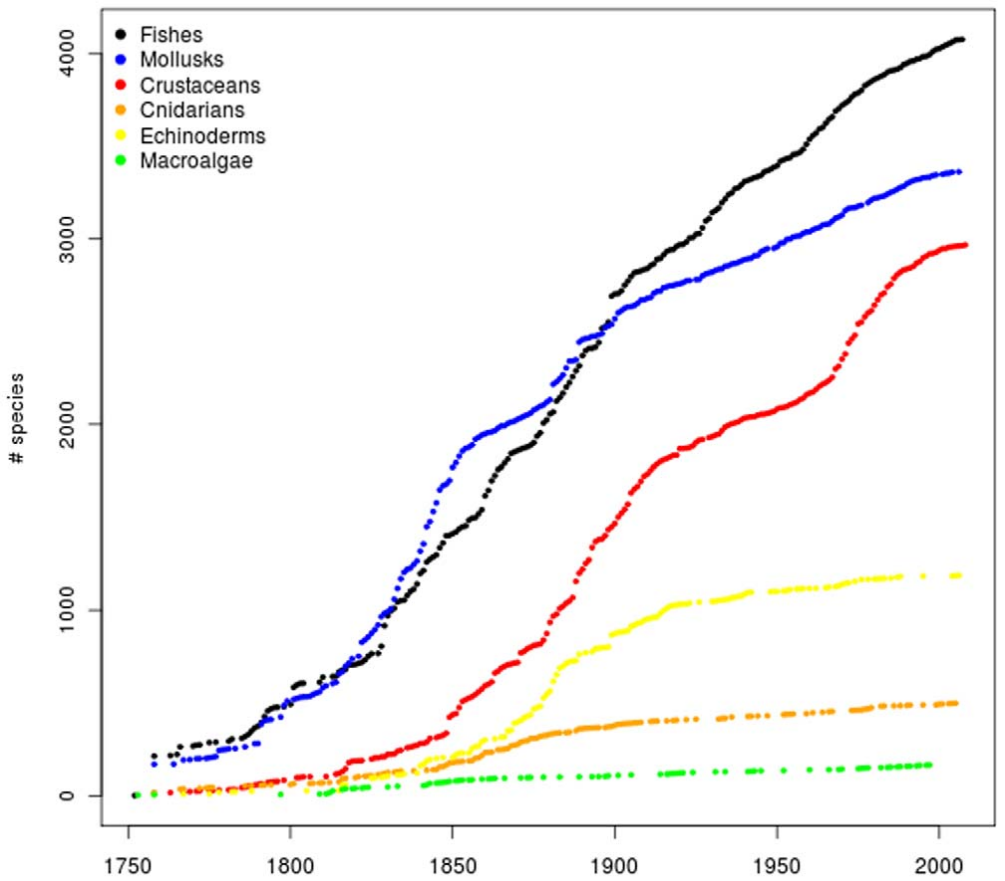
Species-description accumulation curves for South American marine species by taxonomic group (macroalgae, cnidarians, mollusks, crustaceans, echinoderms, and fishes). Data from OBIS database (using only “valid names” which corrects for synonyms).

Description of South American species began as early as the mid-1700s with several peaks of discovery around 1850, 1900, and 1970 (Figure 11a). Since then, new species have been added to the total every year exponentially (Figure 11b). A total of 13,656 species are reported in OBIS for the five subregions considered in this paper. As mentioned, this number could represent about half of the known species of South America. As stated in tables 1, 3, 4, 5, and 6, the best known groups in the region (those ranked mostly between 4 and 5 in the “state of knowledge” category) are fish, mollusks, crustaceans, echinoderms, cnidarians, and macroalgae. The rate of discovery for these best-known taxonomic groups has been variable, and the number of fish, mollusk, and crustacean species is continuously increasing. However, this is not true of cnidarians, echinoderms, and macroalgae, which seem to have reached a relatively stable number, with few new additions (Figure 12). This stability certainly indicates that these groups have been neglected in the region, probably the consequence of a combination of factors, including lack of taxonomic expertise, limited funding for research, lack of collecting effort, and limited access to sampling sites. However, these curves are based in OBIS data which has an iconsistent subset of data for the region, with some regions (e.g. Brazil) better represented than others (e.g. Humboldt Current), so a full species inventory is needed to confirm if these patterns are valid. On the other hand, given the richness of these three groups in the world context (Bouchet, [402] has reported a total of 9,795 cnidarians, 7,000 echinoderms, and 10,300 macroalgae), it seems unlikely that such low numbers represent the total regional biodiversity of these groups for such a vast area as South America. While it is true that new descriptions of some well-known groups such as vertebrates have decreased in the last decade, the application of new molecular methods at a broader global scale, together with the exploration of the less explored environments will undoubtedly help to improve and refine the knowledge on marine biodiversity. In addition, shifts in species distribution associated with climate change are expected to increase in frequency in the near future.

Two interesting questions can be asked about the 13,656 species that compose about half of the known biodiversity of South America. The first is, how many of them are exclusive to one subregion or are shared by two or more subregions, and in which proportion? This is a question of endemism within regions of South America. The second question is, how many of these species are exclusive to South America and in which taxonomic groups? This is a question of South American endemism within a global context. To answer the first question, we sorted the number of species in the OBIS database that are present in one, two, three, four, and five subregions, and how they were distributed (Table 8). A total of 10,311 species are reported to exist exclusively in only one South American subregion, that is, 75.5% of the total species reported for the region. Among the subregions, this endemism within South America represents 71.2% of the species for the Tropical East Pacific (2,452 species), 43.4% for the Humboldt Current (1,691 species), 48.2% for the Tropical West Atlantic (896 species), 71.6% for Brazil (3,921 species), and 42.6% for the Patagonian Shelf (1,351 species). On the other hand, the number of species shared by two or more subregions decreased as the number of subregions involved increased; with 28 species shared by all five subregions (comprising mainly protists, a few cnidarians, and the killer whale, *Orcinus orca*).

**Table 8:** Number of species reported exclusively for the five subregions of South America from the OBIS database.

SUBREGION	1	2	3	4	5
Tropical East Pacific	2452	674	218	74	28
Humboldt Current	1691	1540	453	182	28
Tropical West Atlantic	896	642	372	157	28
Brazilian Shelves	3921	995	358	173	28
Patagonian Shelf	1351	1167	459	166	28
**TOTAL**	**10311**	**2509**	**620**	**188**	**28**

To answer the second question, we filtered from the global database the species that are only found around South America, that is, the species that have not been reported elsewhere in the world. The total number of species that are “endemic” to South America within the global context according to the data in OBIS is 3,065 species, which represents 22.4% of the total reported for the region. These species represent several phyla, of which the most abundant were the mollusks (42%), followed by the arthropods (mainly crustaceans: 23%), and the chordates (fish and other vertebrates: 12%). Polychaetes, cnidarians, sponges, echinoderms, and nematodes accounted altogether for 19% of these “endemic” species. Although this is a good estimate of endemism for the region, the numbers could change as new data are incorporated into the OBIS database. For instance, it is possible that a species considered as “endemic” to South America could have been observed outside the region but that these records have not been published in OBIS. Moreover, with new exploration, species considered to be endemic to South America could appear elsewhere, and would no longer be considered endemic. The total number of endemic species as reviewed in this paper was 886 (67 for the Tropical East Pacific, 197 for the Humboldt Current system, 4 for the Tropical West Atlantic, 446 for Brazil, and 172 for the Patagonian Shelf). These low numbers in relation to what is reported in OBIS as exclusive of South America indicate that regional knowledge about which species are endemic is generally poor, especially for tropical areas, both Pacific and Atlantic. Other regions of extremely high endemism are New Zealand and Antarctica with about 48% of endemic species [445], [446], followed by Australia and South Africa with about 28% of endemic species [447], [448] all of which are located in the Southern Hemisphere as is most of South America. Griffiths et al. [448] reported high levels of species endemism for South African waters (around 4,233 species), a number that is subject to change as some species are being reported in other countries. Among these endemic species, the bryozoans and the mollusks showed high levels of endemism (64% and 56%, respectively), while echinoderms and sponges had much lower levels of endemism (3.6% and 8.8%, respectively). Assuming our estimate of endemism is valid, then South America could be considered as a region of high endemism for mollusks, as has been reported for some localities in Brazil [449]. In New Zealand [445], there are 6,741 endemic species, of which nearly 3,000 are mollusks. In this sense, both New Zealand and South Africa have good knowledge of their species richness and endemism, and South America has yet to attain it. For instance, it has been discussed that seamounts in Brazil seem to be highly endemic (see Bouchet & Leal, [450] for reports on the gastropod fauna of Brazilian seamounts and their reproductive modes, as well as Vaske Jr et al., [235] on deep-water scorpion fish). This raises interesting questions related to reproductive and developpmental strategies, endemism, and faunistic relationships between the Brazilian continental margin and other parts of the Atlantic: Would Brazilian seamounts function as stepping stones in the Atlantic Ocean? How much more endemism do they hold, and what is the relationship between species found on seamounts and those found on the continental margin? Would seamounts act as a gene source or sink? Increasing our knowledge of seamounts would allow us a better understanding of how they function, and provide better baselines for management and conservation, especially if seamounts are repositories of unique biodiversity.

As mentioned earlier, the heterogeneity and vast extent of the South American coast and the diversity of habitats and oceanographic conditions there have important implications for biodiversity. We have discussed the state of knowledge of marine biodiversity, observed latitudinal trends, the potential endemism of the region, and the limits of our knowledge. South America is certainly in a good position to improve its expertise and is likely to advance in some regions, such as Brazil, sooner than in others. National and regional initiatives in new exploration, especially to unknown areas and ecosystems, as well as collaboration between the different countries is fundamental to achieving the goal of completing inventories of species diversity and distribution that will allow accurate interpretation of the biogeography of the continent, latitudinal trends, and differences between its two oceanic coasts. Spalding et al. [52] proposed a bioregionalization of the coastal and shelf areas of the world based in ecoregions. These ecoregions extend beyond national borders and even beyond continents. It would had been interesting to make the same analysis we have done here but comparing among ecoregions instead of the regions used in this paper. However, this is not possible with the present state of knowledge, because most of the data compiled here relate to a specific country rather than to geographic coordinates, as can be found in OBIS. Thus, an extra effort to compile all species records in the literature, validate the taxonomy of these records, and make them available through open-source databases such as OBIS is of outmost importance and must be encouraged and supported by local governments through biodiversity policies. In this paper, we have attempted such a compilation, and in doing so, we have become even more aware of the magnitude of the work still to be done to move on to the next level of knowledge and understanding.

## Supporting Information

Table S1 Sources of information used to estimate total number of marine species for different taxa of the Tropical East Pacific region of South America.
(0.05 MB DOC)

Table S2 Sources of information used to estimate total number of marine species for different taxa of the Patagonian Shelf region of South America.
(0.06 MB DOC)

Table S3 Diversity, state of knowledge, and expertise of all taxonomic groups within the Tropical East Pacific region of South America. Sources of the reports: databases, scientific literature, books, field guides, technical reports. State of knowledge classified as: 5 = very well known (>80% described, identification guides <20 years old, and current taxonomic expertise); 4 = well known (>70% described, identification guides <50 years old, some taxonomic expertise); 3 = poorly known (<50% species described, identification guides old or incomplete, no present expertise within region); 2 = very poorly known (only few species recorded, no identification guides, no expertise); 1 = unknown (no species recorded, no identification guides, no expertise). Taxonomic experts were defined as people with expertise in the description and identification of particular groups of marine species (i.e., taxa).
(0.03 MB XLS)

Table S4 Diversity, state of knowledge, and expertise of all taxonomic groups within the Humboldt Current region of South America. Sources of the reports: databases, scientific literature, books, field guides, technical reports. State of knowledge classified as: 5 = very well known (>80% described, identification guides <20 years old, and current taxonomic expertise); 4 = well known (>70% described, identification guides <50 years old, some taxonomic expertise); 3 = poorly known (<50% species described, identification guides old or incomplete, no present expertise within region); 2 = very poorly known (only few species recorded, no identification guides, no expertise); 1 = unknown (no species recorded, no identification guides, no expertise). Taxonomic experts were defined as people with expertise in the description and identification of particular groups of marine species (i.e., taxa).
(0.05 MB XLS)

Table S5 Diversity, state of knowledge, and expertise of all taxonomic groups within the Patagonian Shelf region of South America. Sources of the reports: databases, scientific literature, books, field guides, technical reports. State of knowledge classified as: 5 = very well known (>80% described, identification guides <20 years old, and current taxonomic expertise); 4 = well known (>70% described, identification guides <50 years old, some taxonomic expertise); 3 = poorly known (<50% species described, identification guides old or incomplete, no present expertise within region); 2 = very poorly known (only few species recorded, no identification guides, no expertise); 1 = unknown (no species recorded, no identification guides, no expertise). Taxonomic experts were defined as people with expertise in the description and identification of particular groups of marine species (i.e., taxa).
(0.03 MB XLS)

Table S6 Diversity, state of knowledge, and expertise of all taxonomic groups within the Brazilian region of South America. Sources of the reports: databases, scientific literature, books, field guides, technical reports. State of knowledge classified as: 5 = very well known (>80% described, identification guides <20 years old, and current taxonomic expertise); 4 = well known (>70% described, identification guides <50 years old, some taxonomic expertise); 3 = poorly known (<50% species described, identification guides old or incomplete, no present expertise within region); 2 = very poorly known (only few species recorded, no identification guides, no expertise); 1 = unknown (no species recorded, no identification guides, no expertise). Taxonomic experts were defined as people with expertise in the description and identification of particular groups of marine species (i.e., taxa).
(0.04 MB XLS)

Table S7 Summary of literature sources on marine biodiversity for the non-coastal Brazilian deep-sea marine realms: (1) slope, (2) seamounts and oceanic islands, and (3) abyssal plains.
(0.09 MB DOC)

Table S8 Major Brazilian cruises that have taken samples in the deep sea, including seamounts and abyssal plains.
(0.06 MB DOC)

Table S9 Diversity, state of knowledge, and expertise of all taxonomic groups within the Tropical West Atlantic region of South America. Sources of the reports: databases, scientific literature, books, field guides, technical reports. State of knowledge classified as: 5 = very well known (>80% described, identification guides <20 years old, and current taxonomic expertise); 4 = well known (>70% described, identification guides <50 years old, some taxonomic expertise); 3 = poorly known (<50% species described, identification guides old or incomplete, no present expertise within region); 2 = very poorly known (only few species recorded, no identification guides, no expertise); 1 = unknown (no species recorded, no identification guides, no expertise). Taxonomic experts were defined as people with expertise in the description and identification of particular groups of marine species (i.e., taxa).
(0.03 MB XLS)
