# Supplementary material for: Marine Biodiversity in the Atlantic and Pacific Coasts of South America: Knowledge and Gaps
Source: PLoS One. 2011 Jan 31;6(1):e14631. doi: 10.1371/journal.pone.0014631 (PMC3031619; doi:10.1371/journal.pone.0014631)
Supplement: Table S1 — Sources of information used to estimate total number of marine species for different taxa of the Tropical East Pacific region of South America. (0.05 MB DOC) [file pone.0014631.s001.doc]

Table S1. Sources of data used to estimate total number of marine species for Porifera, Cnidaria, Annelida, Mollusca, Crustacea, Echinodermata, fish and zooplankton.

|  | **Category** | **Sources** |
| --- | --- | --- |
| **Taxonomic group** | Porifera | [1] |
|  | Cnidaria | [2-9] |
|  | Annelida | [10-12] |
|  | Mollusca | [13-22] |
|  | Arthropoda (mainly Crustaceans) | [15,23-33] |
|  | Echinodermata | [15,34] |
|  | Chordata (Fish, Birds and Ascidians) | [35-43] |
| **Functional group** | Plancton | [19,20,44-51] |
|  | General | [52-60] |

**Literature sources**

1. Zea S, Henkel TP, Pawlik JR (2009) The sponge guide: a picture guide to Caribbean sponges. Available: www.spongeguide.org. Accessed 2010 May 19.

2. Zapata FA, Vargas-Angel B (2003) Corals and coral reefs of the Pacific coast of Colombia. In: Cortés J. Latin American coral reefs. Amsterdam: Elsevier. pp. 419-447.

3. Segura-Puertas L (1984) Morfología sistemática y zoogeografía de las medusas (Cnidaria: Hydrozoa y Sciphozoa) del Pacífico Tropical Orienta. An Inst Cienc del Mar y Limnol Univ Nal Autón México, Publ Esp 8: 1-320.

4. Reyes J (2000) Lista de los corales (Cnidaria: Anthozoa: Scleractinia) de Colombia. Biota Colombiana 1: 164-176.

5. Maté JL (2003) Corals and coral reefs of the Pacific coast of Panama. In: Cortés J. Latin American Coral Reefs. Amsterdam: Elsevier. pp. 387-418.

6. Larson RJ (1990) Scyphomedusae and Cubomedusae from the eastern Pacific. Bull Mar Sci 47: 546-556.

7. Cortés J, Jiménez C (2003) Corals and coral reefs of the Pacific of Costa Rica: history, research and status. In: Cortés J. Latin American Coral Reefs. Amsterdam: Elsevier. pp. 361-386.

8. Bigelow HB (1911) Reports on the scientific results of the expedition to the eastern tropical Pacific, 1904–1905. The Siphonophorae. Bull Mus Comp Zool 38: 171-402.

9. Bigelow HB (1909) Reports on the scientific results of the expedition to the eastern tropical Pacific, 1904–1905. The Medusae. Bull Mus Comp Zool 37: 1-243.

10. Villamar F (1983) Poliquetos bentónicos del Golfo de Guayaquil. Acta Oceanogr Pac, INOCAR 2: 659-733.

11. Salazar-Vallejo SI, Londoño-Mesa MH (2004) Lista de especies y bibliografía de poliquetos (Polychaeta) del Pacífico Oriental Tropical. An Inst Biol Univ Nac Autón México, Ser Zool 75: 9-97.

12. Chamberlin R (1919) Reports on the scientific results of the expedition to the eastern tropical Pacific, 1904–1905. The Annelida, Polychaeta. Mem Mus Comp Zool 48: 1-514.

13. Wood JB, Day CL (2006) Cephbase. Available: http://www.cephbase.utmb.edu/. Accessed 2010 May 19.

14. Mora E (1990) Catálogo de bivalvos marinos del Ecuador. Bol Cient Tec, Instituto Nacional de Pesca, Guayaquil, Ecuador 10: 136.

15. Mair J, Mora E, Cruz M (2002) Manual de campo de los invertebrados bentónicos marinos: Moluscos, Crustáceos y Equinodermos de la zona litoral ecuatoriana. Guayaquil, Ecuador: Univ. de Guayaquil y Heriot Watt University. 105 p.

16. Keen A (1971) Sea Shell of tropical West America Marine mollusks from Baja California to Peru. 2 ed. Standford, California: Stanford University Press. 1064 p.

17. Hertlein L, Strong AM (1955) Marine mollusks collected during the "Askoy" Expedition to Panama, Colombia, and Ecuador in 1941. Bull Am Mus Nat Hist 107: 159-318.

18. Cruz M (1987) Moluscos Bivalvos de la Plataforma Continental de Manabí. Ecuador. Acta Oceanogr Pac 4: 75-101.

19. Cruz M (1983) Pterópodos y Heterópodos del Golfo de Guayaquil. Acta Oceanogr Pac 2: 569-587.

20. Cruz M (1983) Presencia de Pterópodos Tecosomados en el Golfo de Guayaquil. Acta Oceanogr Pac 2: 179-186.

21. Cruz M (1983) Bivalvos del Golfo de Guayaquil. Acta Oceanogr Pac 2: 735-819.

22. Cantera JR, Rubio EA, Borrero FJ, Contreras R, Zapata F, et al. (1979) Taxonomía y distribución de los moluscos litorales en la Isla de Gorgona. In: Prahl HV, Guhl F, Grögl M. Gorgona. Bogotá, Colombia: Universidad De Los Andes. Facultad De Artes Y Ciencias. Futura Grupo Editorial. pp. 141-167.

23. Woltereck R (1909) Reports on the scientific results of the "Albatross" expedition to the eastern tropical Pacific, 1904-1905. XVIII. Amphipoda. Bull Mus Comp Zool 52: pp.

24. Wicksten M, Hendrickx M (2003) An updated checklist of benthic marine and brackish water shrimps (Decapoda: Penaeoidea, Stenopodidea, Caridea) from the Eastern Tropical Pacific. In: Hendrickx M. Contributions to the Study of East Pacific Crustaceans. México: Instituto de Ciencias del Mar y Limnología, UNAM. pp. 49-76.

25. Murillo-Bohórquez C, Alvarez-León R (2004) Nuevos registros y consideraciones biogeográficas de los estomatópodos (Hoplocarida: Stomatopoda) del Pacífico colombiano y comparación con las especies del Caribe colombiano. In: Hendrickx ME. Contributions to the Study of East Pacific Crustaceans. México: Instituto de Ciencias del Mar y Limnología, UNAM. pp. 1-15.

26. Lemaitre R, Álvarez-León R (1992) Crustáceos Decápodos del Pacífico colombiano: Lista de Especies y consideraciones Zoogeográficas. An Inst Invest Mar Punta Betín 21: 33-76.

27. Lemaitre R, Ramos GE (1992) A collection of Thalassinidea (Crustacea : Decapoda) from the Pacific coast of Colombia, with description of a new species and a checklist of eastern Pacific species. Proc Biol Soc Wash 105: 343-358.

28. Lazaruz-Agudelo J, Cantera-Kintz JR (2007) Crustáceos (Crustacea: Sessilia, Stomatopoda, Isopoda, Amphipoda, Decapoda) de Bahía Málaga, Valle del Cauca (Pacífico colombiano). Biota Colombiana 8: 221-239.

29. Haig J (1957) The porcellanid crabs of the "Askoy" Expedition to the Panama Bight. Am Mus Novit 1865: 1-17.

30. Garth JS (1948) The Brachyura of the Askoy Expedition with remarks on carcinological collecting in the Panama Bight. Bull Amer Mus Nat Hist 92: 1-66.

31. Finnegan S (1931) Report on the Brachyura collected in Central America, the Gorgona and Galapagos Islands, by Dr. Crossland on the "St. George" Expedition to the Pacific, 1924-1925. Linn Soc J Zool 37: 607-673.

32. Espinosa-Pérez M, Hendrickx M (2001) Checklist of isopods (Crustacea: Peracarida: Isopoda) from the Eastern Tropical Pacific. Belg J Zool 131: 43-55.

33. Cole LJ (1909) Reports on the scientific results of the "Albatross" expedition to the eastern tropical Pacific, 1904-1905. XIX. Pycnogonida. Bull Mus Comp Zool 52: pp.

34. Clark HL (1917) Reports on the scientific results of the expedition to the tropical Pacific, 1899-1900. XVlll. Reports on the scientific results of the expedition to the eastern Tropical Pacific, 1904-1905. XXX. Ophiuroidea. Bull Mus Comp Zool 61: pp.

35. Fischer W, Krup F, Schneider W, Sommer C, Carpenter KE, et al. (1995) Guia FAO para la Identificacion de Especies de para los fines de la Pesca. Pacifico Centro-Oriental. Volumen II. Vertebrados - Parte 1. 647-1200

36. Froese R, Pauly D (2010) FishBase. World Wide Web electronic publication. Available: www.fishbase.org. Accessed 2010 May 19.

37. Little M, Herrera M (1991) Checklist and Catalogue of fishes recorded in the by- catch of the Ecuadorian shrimp flett. Guayaquil, Ecuador: Overseas Development Administration, Instituto Nacional de Pesca (INP).

38. Massay SH (1983) Revisión de la lista de peces marinos del Ecuador. Bol Cient Tec, Instituto Nacional de Pesca, Guayaquil, Ecuador 6: 1-113.

39. Millar RH (1998) Ascidians collected during the South-east Pacific Biological Oceanographic Program (SEPBOP). J Nat Hist 22: 225-240.

40. Nichols JT, Murphy RC (1944) A collection of fishes from the Panama Bight, Pacific Ocean. Bull Amer Mus Nat Hist 83: 221-260.

41. Robertson DR, Allen GR (2002) Shorefishes of the tropical eastern Pacific: An information system. CD-ROM. Balboa, Panamá: Smithsonian Tropical Research Institute.

42. Robertson DR, Allen GR (2008) Shorefishes of the tropical eastern Pacific online information system. Version 1.0. Smithsonian Tropical Research Institute, Balboa, Panamá. Available: www.neotropicalfishes.org/sftep. Accessed 2010 May 19.

43. Salaman P, Donegan T, Caro D (2008) Listado de las aves de Colombia 2008. Conservación Colombiana 5: 1-85.

44. Boltovskoy D, Jankilevich SS (1985) Radiolarian distribution in east equatorial Pacific plankton. Oceanol Acta 8: 101-123.

45. Gualancañay E (1975) Foraminíferos bentónicos actuales de Ecuador. Provincia de Esmeraldas. Publicación INOCAR. CM-BIO-5.

46. Gualancañay E (1983) Foraminíferos bentónicos del Golfo de Guayaquil. Acta Oceanogr Pac 2: 589-657.

47. Guiry MD, Guiry GM (2010) AlgaeBase. World-wide electronic publication, National University of Ireland, Galway. Available: http://www.algaebase.org. Accessed 2010 May 19.

48. Jiménez R (1983) Diatomeas y Silicoflagelados del fitoplancton del Golfo de Guayaquil. Acta Oceanogr Pac 2: 193-281.

49. Kofoid CA, Campbell B (1939) Reports on the scientific results of the expedition to the eastern tropical Pacific, 1904–1905. The Ciliata: the Tintinnoinea. Bull Mus Comp Zool 84: 1-473.

50. Luzuriaga De Cruz M (1976) Foraminíferos planctónicos vivos en aguas superficiales ecuatorianas durante El Niño de 1972. Inst Ocean Armada, CM Bio 09: 1-30.

51. Zambrano I (1983) Tintínidos del Golfo de Guayaquil. Acta Oceanogr Pac, INOCAR 2: 443-507.

52. Appeltans W, Bouchet P, Boxshall GA, Fauchald K, Gordon DP, et al. (2010) World Register of Marine Species. Available: http://www.marinespecies.org. Accessed 2010 May 19.

53. Cantera J, Arnauld P, Neira R (1992) La macrofauna de playas arenosas en las bahías de Buenaventura y Málaga (Pacífico colombiano): Estructura espacial y dinámica temporal. Mem Semin Nac Cienc Tecnol Mar Bogotá: 225-241.

54. UBC (2009) SeaLifeBase. Available: http://www.sealifebase.org/home/pages/index.htm. Accessed 2010 May 19.

55. Costello MJ, Stocks K, Zhang Y, Grassle JF, Fautin DG (2007) About the Ocean Biogeographic Information System. Available: www.iobis.org. Accessed 2010 May 19.

56. FAO (1982) Catálogo de especies marinas de interés económico o potencial para América Latina. Parte II: Pacífico Centro y Sur oriental. Roma: Programa de las Naciones Unidas para el desarrollo. Organización de las Naciones Unidas. 503 p.

57. INVEMAR (2006) SIBM [en línea]: Sistema de Información sobre Biodiversidad Marina. [Santa Marta]: Instituto de investigaciones Marinas y Costeras “José Benito Vives de Andréis”. Available: http://siam.invemar.org.co/siam/sibm/index.htm. Accessed 2010 May 19.

58. ITIS (2009) Integrated Taxonomic Information System. Available: http://www.itis.gov/. Accessed 2010 May 19.

59. Wehrtmann I, Cortés J (2009) Marine Biodiversity of Costa Rica, Central America. Monographiae Biologicae, Vol. 86. Berlin: Springer. 538 p.+CDROM

60. West RC (1957) The Pacific lowlands of Colombia: a negroid area of the american tropics. Baton Rouge: Louisiana State University Press. 278 p.
