# Supplementary material for: Marine Biodiversity in the Atlantic and Pacific Coasts of South America: Knowledge and Gaps
Source: PLoS One. 2011 Jan 31;6(1):e14631. doi: 10.1371/journal.pone.0014631 (PMC3031619; doi:10.1371/journal.pone.0014631)
Supplement: Table S2 — Sources of information used to estimate total number of marine species for different taxa of the Patagonian Shelf region of South America. (0.06 MB DOC) [file pone.0014631.s002.doc]

Table S2. Sources of information used to estimate total number of marine species for different taxa of the Patagonian Shelf region of South America.

|  | **Institution** | **Address** | **WEB site** |
| --- | --- | --- | --- |
| **Research Institutions** |  |  |  |
| CADIC | Centro Austral de Investigaciones Científicas | Avda. Malvinas Argentinas s/n. - C.C.92 (V9410BFD) Ushuaia | [www.tierradelfuego.org.ar/cadic](http://www.tierradelfuego.org.ar/cadic) |
| CENPAT | Centro Nacional Patagónico | Boulevard Brown s/n (U9120ACV) Puerto Madryn, Chubut | [www.cenpat.edu.ar](http://www.cenpat.edu.ar/) |
| IBMyP | Instituto de Biología Marina y Pesquera Almirante Storni |  |  |
| IAA | Instituto Antártico Argentino | Cerrito 1248, (1010) Buenos Aires, Argentina | [www.dna.gov.ar](http://www.dna.gov.ar/) |
| INIDEP | Instituto Nacional de Investigación y Desarrollo Pesquero | Paseo Victoria Ocampo Nº 1, (7600) Mar del Plata | [www.inidep.edu.ar](http://www.inidep.edu.ar/) |
| MACN | Museo Argentino de Ciencias Naturales  Bernardino Rivadavia | Avenida Angel Gallardo 470, (C1405DJR) Buenos Aires | [www.macn.secyt.gov.ar](http://www.macn.secyt.gov.ar/) |
| UBA | Universidad de Buenos Aires | Ciudad Universitaria, (C1428EHA) Buenos Aires | [www.uba.edu.ar](http://www.uba.edu.ar/) |
| UNMdP | Universidad Nacional de Mar del Plata | Funes 3350, (7600) Mar del Plata | [www.mdp.edu.ar](http://www.mdp.edu.ar/) |
| UNPSJB | Universidad Nacional de la Patagonia San Juan Bosco (Sede Comodoro Rivadavia) | Km 4, (9000) Comodoro Rivadavia, Argentina | [www.unp.edu.ar](http://www.unp.edu.ar/) |
| UNPA | Centro de investigaciones UNPA | Puerto Deseado, Santa Cruz |  |
| DINARA | Dirección Nacional de Recursos Acuáticos | Constituyente 1497, C.P. 11200, Montevideo, Uruguay | http://www.dinara.gub.uy |
| FCIEN | Facultad de Ciencias, Universidad de la República | Igua 4225, CP 11400, Montevideo, Uruguay | <http://www.fcien.edu.uy/> |
| MNHN | Museo Nacional de Historia Natural | C.C. 399, C.P. 11000, Montevideo, Uruguay | <http://www.mec.gub.uy/munhina/> |
|  |  |  |  |
| **Conservation NGOs** |  |  |  |
| CETHUS | Fundación Cethus | Juan de Garay 2861, 3 (1636) Olivos, Buenos Aires | www.cethus.tripod.com |
| Ecocentro | Ecocentro Puerto Madryn | Julio Verne 3784 (U9120OJA), Puerto Madryn, Chubut. | www.ecocentro.org.ar |
| FVS | Fundación Vida Silvestre (Programa Marino) | Córdoba 2920 4 B.(B7602CAD) Mar del Plata, Buenos Aires | [www.vidasilvestre.org.ar](http://www.efpu.com.ar/) |
| FPN | Fundación Patagonia Natural | Marcos A. Zar 760. (U9120ACV) Puerto Madryn | [www.patagonianatural.org](http://www.undp.org/rblac/wssd)) |
|  |  |  |  |
| ICB | Instituto de Conservación de las Ballenas | García Merou 833 (1640) Martinez, Buenos Aires | www.icb.org.ar |
| AA | Aves Argentina | Matheu 1246/8 – Ciudad Autónoma de Buenos Aires | [www.­avesargentinas.­org.­ar](http://www.google.com/url?sa=D&q=http://www.avesargentinas.org.ar/&usg=AFQjCNGmumYAdQuEpLk680vkKc5ncAsVAQ) |
