# Supplementary material for: Marine Biodiversity in the Atlantic and Pacific Coasts of South America: Knowledge and Gaps
Source: PLoS One. 2011 Jan 31;6(1):e14631. doi: 10.1371/journal.pone.0014631 (PMC3031619; doi:10.1371/journal.pone.0014631)
Supplement: Table S7 — Summary of literature sources on marine biodiversity for the non-coastal Brazilian deep-sea marine realms: (1) slope, (2) seamounts and oceanic islands, and (3) abyssal plains. (0.09 MB DOC) [file pone.0014631.s007.doc]

Table S7. Summary of literature sources on marine biodiversity for the non-coastal Brazilian marine realms: (1) slope, (2) seamounts and oceanic islands, and (3) abyssal plains.

| **AREAS** | **SUBJECT / REFERENCE** |
| --- | --- |
| 1. **SLOPE** | General information on continental margins [1-4]. |
|  | Composition and distribution of macrobenthic invertebrates from the upper slope of Rio Grande do Sul down to 500m [5]. |
|  | Macrobenthic associations on the upper slope down to 600m in the State of São Paulo [6]. |
|  | Fauna of the slope between 100 to 1500m at the northern sector of Vitoria-Trindade, south of the Abrolhos Plateau, near the estuary of the Rio Doce River, and near Cabo Frio in the ACW upwelling zone [7]. |
|  | Deep-sea shrimp (Crustacea: Penaeidea and Caridea) from the North region [8]. |
|  | Deep sea micro-mollusks obtained through the “Challenger Expedition” (1873-1876) [9]. |
|  | List of 21 sponge species found in the Northeast slope from (116 to 3138m) mostly sampled through the Challenger, Albatross and REVIZEE (*) expeditions [10]. |
|  | Reporting of nearly 600 macro- and 300 mega-invertebrate taxa in an approximately 30.000 m2 sampled area at Campos Basin slope [11]. |
|  | Studies on the benthic structure of the Campos Basin (20.5-24°S and 40-41°W, and geophysical description of the basin [12-14]. |
|  | Taxa composition of the Campos basin between 1050-1950 m: 570 taxa of marine macro-invertebrates represented by 227 gastropods mainly Turridae (48) and Skeneidae (33), 79 bivalves the richest family being Cuspidariidae (15), 1 cephalopod, 1 aplacophoran and 14 scaphopods (none with soft tissue); 144 crustacean taxa (64 isopod, 42 amphipod, 13 cumacean, 29 tanaidacean, 3 maxilopod, and 3 decapod morphotypes); 91 polychaete taxa, being Spionidae the most representative family [15]. |
|  | Benthic megafauna composition and abundance (trawled in a 1.2 km2 area of the Campos Basin): total of 9.132 megafaunal organisms from 280 taxa representing 72 crustaceans (39 decapods), 41 cnidarians (19 octocorals), 52 mollusks (31 gastropods), 39 echinoderms (18 ophiuroids), 17 sponges (10 Desmopongiae and 7 Hexactinellida), and 18 polychaetes [16]. |
|  | First species reports: nematodes [17,18], decapods [19], echinoderms [20], squat lobster [21], galatheids [22], fish [23]. |
| 1. **SEAMOUNTS AND OCEANIC ISLANDS** | Relationship between the presence of seamounts and oceanic islands and local upwelling leading to higher regional productivity [24-26]. |
|  | Importance of seamounts for fisheries and as diversity hotspots in all levels of the trophic web [25,27-30]. |
|  | Productivity of the waters near the Northern, Fernando de Noronha, and Vitoria-Trindade Ridges [24]. |
|  | Reports on zooplankton, shallow benthic, and nektonic fauna (shallow and deep) around the Northern and Fernando de Noronha Ridges [31,32]. |
|  | Density, biomass, and diversity of mesozooplankton species in northeastern Brazilian waters [32-34]. |
|  | Productivity and economical and ecological importance [35,36]. |
|  | Fish, polychaetes, molluscs and crustaceans from the seamount Atol das Rocas, within the Fernando de Noronha ridge [37-40]. |
|  | Importance of coral reefs as fisheries resources in the Northeast [41] and general knowledge on oceanic islands reef fishes [42-50]. Fish endemism in these reef areas [45,51]. |
|  | Fernando da Noronha ridge: general community composition [24,52], fish from Trindade Island [50,53], fish endemism [47,54], fish distribution and abundance, reproduction and development, feeding habits, growth [55-60], spatial and temporal abundance of tunas, sharks, billfishes and other fish species caught by Brazilian longliners [61], fish vulnerability to exploitation [62,63]. |
|  | Macroalgae in Atol da Rocas and Fernando da Noronha Archipielago [64], sponges and associated invertebrates from Atol das Rocas, Fernando de Noronha, and St Peter and St Paul Archipielago [65-70], mollusks from these areas [71,72], oceanic cephalopods [73]. |
|  | The Victoria-Trindade Ridge: results of the Franco-Brazilian Deep Sea Expedition Marion Dufresne (MD55) to the seamounts (summit, slope and adjacent abyssal plain) [4,74,75], general community composition [52], macroalgae [64,76], sponges [77], cnidarians [78-80], polychaetes [37], mollusks [81], bivalves [82], gastropods [83], crustaceans [75,84-89], echinoderms (mostly ophioroids) [90-94], fish (Muraenidae and Gobiidae were the dominant families) [4]. |
|  | Gastropods and information on their reproductive strategies from several seamounts (Victoria, Davis, Trindade Columbia, and Martin Vaz) [95]. |
|  | Fish diversity and fisheries in several seamounts [96-99]. |
| 1. **ABYSSAL PLAIN** | Results of the sampling by the MD55 cruise at depths up to 5100 m [4,7]. |
|  | Sediment type in sampled abyssal plains [92]. |

(*) REVIZEE: Living Resources in the Exclusive Economic Zone of Brazil program

**References**

1. Miyaji C (2001) Gastrópodes prosobrânquios da plataforma continental externa e talude superior da costa sudeste brasileira. São Paulo: Tese de Doutorado. Universidade de São Paulo, Instituto Oceanográfico. 128 p.

2. Levin LA, Gooday AJ (2003) The deep Atlantic Ocean. In: Tyler P. Ecosystems of the deep ocean. Ecosystems of the World Series n28. Amsterdam: Elsevier. pp. 118-178.

3. Lana PC, Camargo MG, Brogim RA, Isaac VJ (1996) O Bentos da Costa Brasileira. Avaliação Crítica e Levantamento bibliográfico (1858-1996). Rio de Janeiro: Programa Revizee. MMA/CIRM/FEMAR. 431 p.

4. Guille A, Ramos JM (1987) Les rapports des campagnes à la mer MD55/Brésil à bord du “Marion Dufresne” – 6 mai-2 juin 1987. Terres Australes et Antarctiques Françaises 87: 1-198.

5. Capítoli RR, Bonilha LE (1991) Comunidades bentônicas. In: Vooren CM. Projeto Talude. FIPEC Relatório final. Rio Grande: FURG. pp. 79-92.

6. Sumida PY, Pires-Vanin AM (1997) Benthic associations of the shelfbreak and upper slope off Ubatuba-SP, South-eastern Brazil. Estuarine, Coastal and Shelf Science 44: 779-784.

7. Bouchet P (1987) Presentation de L’Etude Du Systeme Benthique. In: Guille A, Ramos J. Les rapports des campagnes à la mer MD55/Brésil à bord du “Marion Dufresne” – 6 mai-2 juin 1987. Terres Australes et Antarctiques Françaises. pp. 87(3): 119-126.

8. Ramos-Porto M, Silva KC, Viana GF, Cintra IH (2000) Camarões de profundidade coletados no Norte do Brasil (Crustacea: Penaeidea e Caridea). Trab oceanogr Univ Fed Pernambuco 28: 71-85.

9. Barros JC, Santos FN, Santos MC, Cabral E, Acioli FD (2001) Redescoberta de moluscos obtidos durante a “Challenger Expedition” (1873-1876): micromoluscos de águas profundas. Boletim Técnico-Científico do CEPENE, Tamandaré 9: 9-24.

10. Hajdu E, Lopes DA (2007) Checklist of Brazilian deep-sea sponges. In: Custódio MR, Lôbo-Hajdu G, Hajdu E, Muricy G. Porifera research - biodiversity, innovation and sustainability. São Paulo: CNPq. pp. 353-359.

11. Falcão AP, Curbelo Fernandez MP, Lavrado HP, Ferreira VP, Campos LS, et al. (2010) Antecedentes, histórico e descritivo do projeto de caracterização ambiental de águas profundas da Bacia de Campos/Petrobras. Projeto de Caracterização Ambiental de Águas Profundas da Bacia de Campos/ PETROBRAS. CENPES/PETROBRAS. p. in press.

12. Lavrado HP, Brasil AC (2010) Biodiversidade da região oceânica profunda da Bacia de Campos: Megafauna e Ictiofauna demersal. Rio de Janeiro: SAG Serv. 376 p.

13. Lavrado HP, Brasil AC (2010) Biodiversidade da região oceânica profunda da Bacia de Campos: Macrofauna. Rio de Janeiro: SAG Serv. 232 p.

14. Arantes RC, Castro CB, Pires DO, Seoane JC (2009) Depth and water mass zonation and species associations of cold-water octocoral and stony coral communities in the southwestern Atlantic. Mar Ecol Prog Ser 397: 71-79.

15. Lavrado HP, Brasil AC, Curbelo Fernandez MP, Campos LS (2010) Aspectos gerais da macrofauna bentônica da Bacia de Campos. In: Lavrado HP, Brasil AC. Biodiversidade da região oceânica profunda da Bacia de Campos: Macrofauna. Rio de Janeiro: SAG Serv. pp. 19-27.

16. Lavrado HP, Brasil AC, Curbelo Fernandez MP, Campos LS (2010) Aspectos gerais da macrofauna bentônica da Bacia de Campos. In: Lavrado HP, Brasil AC. Biodiversidade da região oceânica profunda da Bacia de Campos: Megafauna e Ictiofauna demersal. Rio de Janeiro: SAG Serv. pp. 21-29.

17. Botelho AP, Silva MC, Sobral LD, Fonsêca-Genevois VG (2009) Two new species of *Sabatieria* Rouville (Nematoda: Comesomatidae) with conical cylindrical tails, from Campos Basin, Rio de Janeiro, Brazil. Zootaxa 2096: 82-98.

18. Botelho AP, Silva MC, Esteves AM, Fonsêca-Genevois V (2007) Four new species of *Sabatieria* Rouville, 1903 (Nematoda, Comesomatidae) from the continental slope of Atlantic Southeast. Zootaxa 1402: 39-57.

19. Manning RB, Tavares MS, Albuquerque EF (1989) *Chaceon Ramosae*, a new deep-water Crab from Brazil (Crustacea: Decapoda: Geryonidae). Proc Biol Soc Wash 102: 646-650.

20. Campos LS, Moura RB, Alcântara PF, Vasconcelos RF, Curbelo-Fernandez MP, et al. (2010) On the two new records of Family Brisingidae (Echinodermata: Asteroidea) from the Brazilian continental margin. In: Harris LG, Bottger SA, Walker CW, Lesser MP. Echinoderms: Durham. New Hampshire: CRC Press / Taylor & Francis Group. pp. 139-146.

21. Tavares M, Melo-Filho GA, Melo GA (2008) The deep-sea squat lobster *Munidopsis transtridens* Pequegnat and Pequegnat, 1971 (Decapoda: Anomura: Galatheidae) from the Southwestern Atlantic. Nauplius 16: 95-99.

22. Abreu Junior CR (2006) Taxonomia e distribuição da família Galatheidae (Crustacea: Decapoda: Anomura) coletados pelo programa REVIZEE – Score Central entre as Latitudes 11ºs - 22ºs. Rio de Janeiro: Dissertação de Mestrado, Programa de Pós-Graduação em Ciências Biológicas (Zoologia) do Museu Nacional da Universidade Federal do Rio de Janeiro. 99 p.

23. Caíres RA, Figueiredo JL, Bernardes RA (2008) Registros novos e adicionais de teleósteos marinhos na costa brasileira. Papéis Avulsos Zool, S Paulo 48: 213-225.

24. Rossi-Wongtschowski CL, Madureira LS (2006) O ambiente oceanográfico da plataforma continental e do talude na região Sudeste-Sul do Brasil. São Paulo: Editora da Universidade de São Paulo. 472 p.

25. Travassos P, Hazin FH, Zagaglia JR, Adivíncula R, Schober J (1999) Thermohaline structure around seamounts and islands off northeast Brazil. Arch Fish Mar Res 47: 211-222.

26. Valentin JL (2001) The Cabo Frio Upwelling System, Brazil. In: Seeliger U, Kjerfve B. Coastal Marine Ecosystems of Latin America. Berlin: Springer. pp. 97-105.

27. Hazin FH, Fischer AF, Broadhurst MK (2001) Aspects of reproductive biology of the scalloped hammerhead shark, *Sphyrna lewini*, off northeastern Brazil. Environmental Biology of Fishes 61: 159-159.

28. Hazin FH, Hazin HG, Travassos P (2007) CPUE and catch trends of shark species caught by Brazilian longliners in the Southwestern Atlantic Ocean. Col Vol Sci Pap ICCAT 60: 636-647.

29. Hazin FH, Travassos PE (2006) Aspectos estratégicos para o desenvolvimento da pesca oceânica no Brasil. Parcerias Estratégicas 11: 289-309.

30. Ferreira BP, Hazin FH (2003) Recursos vivos marinhos do Nordeste do Brasil. In: Leça EE, Leitão SN, Ferreira Da Costa M. Livro texto de Oceanografia, comemorativo dos 50 anos do Departamento de Oceanografia da UFPE. Recife-PE: Editora da UFPE, v. 1.

31. Carvalho FC, Oliveira PG, Hazin FH, Piercy A, Burgess GH, et al. (2008) Population structure, size and habitat utilization of the Southern Stingray, Dasyatis americana, Hildebrand & Schroeder, 1928, at the Atol das Rocas Biological Reserve, Brazil. Braz J Oceanogr. in press.

32. Neumann-Leitão S, Gusmão LM, Silva T, Nascimento-Vieira DA, Silva AP (1999) Mesozooplankton biomass and diversity in coastal and oceanic waters off North-Eastern Brazil. Arch Fish Mar Res 47: 153-165.

33. Ekau W, Knoppers B (1999) An introduction to the pelagic system of the North-East and East Brazilian shelf. Arch Fish Mar Res 47: 113-132.

34. Cavalcanti EA, Larrazábal ME (2004) Macrozooplâncton da zona econômica exclusiva do Nordeste do Brasil (segunda expedição oceanográfica– REVIZEE/NE II) com ênfase em Copepoda (Crustacea). Rev Bras Zool 21: 467-475.

35. Prates AP, Blanc D (2007) Áreas aquáticas protegidas como instrumento de gestão pesqueira. Série Áreas Protegidas do Brasil, 4. Brasília: MMA/SBF. 272 p.

36. Rogers AD (1994) The biology of seamounts. Adv Mar Biol 30: 305-350.

37. Paiva PC, Young PS, Echeverria CA (2007) The Rocas Atoll, Brazil: a preliminary survey of the Crustacea and Polychaeta fauna. Arq Mus Nac 65: 241-250.

38. Barroso R, Paiva PC (2007) Amphinomidae (Annelida: Polychaeta) from Rocas Atoll, northeastern Brazil. Arq Mus Nac 65: 3567-362.

39. Costa-Paiva EM, Paiva PC (2007) *Sabellidae latreille*, 1825 (Annelida, Polychaeta) from Rocas Atoll, Brazil, with the description of a new species. Arq Mus Nac 65: 363-368.

40. Kikuchi RK (1999) Atol das Rocas, Atlântico sul equatorial ocidental, Brasil. In: Schobbenhaus C, Campos DA, Queiroz ET, Winge M, Berbert-Born M. Sítios Geológicos e Paleontológicos do Brazil / Geological and Paleontological Sites of Brazil. Available: http://www.unb.br/ig/sigep/sitio033/sitio033.htm. Accessed 2009 Jul 20.

41. Ferreira BP, Maida M (2007) Characteristics and perspectives for fishery management in the coral coast marine protected area. In: Prates AP, Blanc D. Áreas aquáticas protegidas como instrumento de gestão pesqueira. Série Áreas Protegidas do Brasil, 4. Brasília: MMA/SBF. pp. 39-50.

42. Frédou FL, Frédou T, Travassos P, Lins J, Arfelli C, et al. (2007) Distribution, catch and length composition of the albacore tuna (*Thunnus alalunga*) caught by the tuna longline fishery in the South Atlantic Ocean. Col. Vol. Sci. Pap. ICCAT 60: 518-526.

43. Joyeux JC, Floeter SR, Ferreira CE, Gasparini JL (2001) Biogeography of tropical reef fishes: the South Atlantic puzzle. J Biogeogr 28: 831-841.

44. Floeter SR, Vázquez DP, Grutter AS (2007) The macroecology of marine cleaning mutualisms. J Anim Ecol 76: 105-111.

45. Floeter SR, Krohling W, Gasparini JL, Ferreira CE, Zalmon I (2007) Reef fish community structure on coastal islands of the southeastern Brazil: the influence of exposure and benthic cover. Environ Biol Fish 78: 147-160.

46. Floeter SR, Guimarães RZ, Rocha LA, Ferreira CE, Rangel CA, et al. (2001) Geographic variation in reef-fish assemblages along the Brazilian coast. Global Ecol Biogeogr 10: 423-431.

47. Floeter SR, Gasparini JL (2000) The southwestern Atlantic reef fish fauna: composition and zoogeographic patterns. J Fish Biol 56: 1099-1115.

48. Floeter SR, Ferreira CE, Dominici-Arosemena A, Zalmon I (2004) Latitudinal gradients in Atlantic reef fish communities: trophic structure and spatial use patterns. J Fish Biol 64: 1680-1699.

49. Floeter SR, Behrens MD, Ferreira CE, Paddack MJ, Horn MH (2005) Geographical gradients of marine herbivorous fishes: patterns and processes. Mar Biol 147: 1435-1447.

50. Rocha LA (2003) Patterns of distribution and processes of speciation in Brazilian reef fishes. J Biogeogr 30: 1161-1171.

51. Moura RL (2002) Brazilian reefs as priority areas for biodiversity conservation in the Atlantic Ocean. Proc 9th Int Coral Reef Symp. Bali, Indonesia 2: 917-920.

52. Lavrado HP (2006) Caracterização do ambiente e da comunidade bentônica. In: Lavrado HP, Ignácio BL. Biodiversidade bentônica da região central da Zona Econômica Exclusiva brasileira. Série Livros n.18. Rio de Janeiro: Museu Nacional - UFRJ. pp. 19-64.

53. Rocha LA, Rosa IL (2001) No Title. J Fish Biol 58: 985-998.

54. Sampaio CL, Carvalho-Filho A, Feitoza BM, Ferreira CE, Floeter SR, et al. (2006) Peixes recifais endêmicos e ameaçados das ilhas oceânicas brasileiras e do complexo recifal dos Abrolhos. In: Alves RJ, Castro JW. Ilhas Oceânicas Brasileiras – da Pesquisa ao Manejo. Brasília DF: Ministério do Meio Ambiente, Secretaria de Biodiversidade e Florestas. pp. 217-234.

55. Freitas RH, Rosa RS, Gruber SH, Wetherbee BM (2006) Early growth and juvenile population structure of lemon sharks *Negaprion brevirostris* in Atol das Rocas Biological Reserve, off north-east Brazil. J Fish Biol 68: 1319-1332.

56. Garla RC, Garcia-Jr J, Veras LB, Lopes NP (2009) Fernando de Noronha as an insular nursery area for lemon sharks, *Negaprion brevirostris*, and nurse sharks, Ginglymostoma cirratum, in the equatorial western Atlantic Ocean. Mar Biodiver Rec 2: e109. Available: http://journals.cambridge.org/action/displayAbstract?fromPage=online&aid=5608724. Accessed 2010 Jan 20.

57. Hazin FH, Boeckman CE, Leal EC, Lessa RT, Kihara K, et al. (1994) Distribution and relative abundance of the blue shark, *Prionace glauca*, in the southwestern equatorial Atlantic Ocean. Fish Bull 92: 474-480.

58. Hazin FH, Couto AA, Kihara K, Otsuka K, Ishino M (1990) Distribution and abundance of pelagic sharks in the southwestern equatorial Atlantic. J Tokyo Univ Fish 77: 51-64.

59. Hazin FH, Kihara K, Otsuka K, Boeckman CE, Leal EC (1994) Reproduction of the blue shark, *Prionace glauca*, in the southwestern equatorial Atlantic Ocean. Fish Sci (Tokyo) 60: 487-491.

60. Hazin FH, Lessa RT, Chammas M (1994) First observation on stomach contents of blue shark, *Prionace glauca*, from southwestern equatorial Atlantic. Rev Bras Biol 54: 195-198.

61. Hazin FH, Zagaglia JR, Broadhurst MK, Travassos PE, Bezerra TR (1998) Review of a small-scale pelagic longline fishery off Norhteastern Brazil. Mar Fish Rev 60: 1-8.

62. Morato T, Cheung WW, Pitcher TJ (2006) Vulnerability of seamount fish to fishing: fuzzy analysis of life-history attributes. J Fish Biol 68: 209-221.

63. Johnston PA, Santillo D (2004) Conservation of seamount ecosystems: application of a marine protected areas concept. Arch Fish Mar Res 51: 305-319.

64. Villaça R, Pedrini AG, Pereira SM, Figueiredo MA (2006) Flora marinha bentônica das Ilhas Oceânicas brasileiras. In: Alves RJ, Castro JW. Ilhas Oceânicas Brasileiras – da Pesquisa ao Manejo. Brasília-DF: Brasília, Ministério do Meio Ambiente/ Sociedade Brasileira de Ficologia. pp. 106-146.

65. Moraes F, Ventura M, Klautau M, Hajdu E, Muricy G (2006) Biodiversidade de esponjas das Ilhas Oceânicas Brasileiras. In: Alves RJ, Castro JW. Ilhas Oceânicas Brasileiras – da Pesquisa ao Manejo. Brasília-DF: Ministério do Meio Ambiente, Secretaria de Biodiversidade e Florestas. pp. 149-177.

66. Moraes FC, Muricy G (2003) Taxonomy of Plakortis and Plakinastrella (Demospongiae: Plakinidae) from oceanic islands off north-eastern Brazil, with description of three new species. J Mar Biol Assoc UK 83: 385-397.

67. Moraes FC, Vilanova E, Muricy G (2003) Distribuição das esponjas (Porifera) da Reserva Biológica do Atol das Rocas. Arq Mus Nac 61: 13-22.

68. Mothes B, Bastian MC (1993) Esponjas do Arquipélago de Fernando de Noronha, Brasil (Porifera, Demospongiae). Iheringia, Sér. Zool., Porto Alegre 75: 15-31.

69. Esteves E, Moraes FC, Muricy G, Amaral F (2002) Duas novas ocorrências da Ordem Hadromerida (Porifera, Demospongiae) no Arquipélago de São Padro e São Paulo, Brasil. Bol Mus Nac, NS, Zool, Rio de Janeiro 488: 1-12.

70. Eston VR, Migotto AE, de Oliveira Filho EC, de Almeida Rodriguez S, Freitas JC (1986) Vertical distribution of benthic marine organismson rocky coasts of Fernando de Noronha Archipelago (Brazil). Bol Inst Ocenogr 34: 37-53.

71. Leite TS, Haimovici M (2006) Biodiversidade e Habitat dos Polvos de Águas Rasas das Ilhas Oceânicas do Nordeste Brasileiro. In: Alves RJ, Castro JW. Oceânicas Brasileiras – da Pesquisa ao Manejo. Brasília-DF: Ministério do Meio Ambiente, Secretaria de Biodiversidade e Florestas,. pp. 201-214.

72. Gomes RS, Costa PM, Monteiro JC, Coelho AC, Salgado NC (2006) Moluscos das Ilhas Oceânicas Brasileiras. In: Alves RJ, Castro JW. Ilhas Oceânicas Brasileiras – da Pesquisa ao Manejo. Brasília-DF. pp. 180-198.

73. Vaske Jr. T, Lessa RP, Nóbrega MF, Montealegre-Quijano S, Santana FM, et al. (2005) A checklist of fishes from Saint Peter and Saint Paul Archipelago, Brazil. J Appl Ichthyol 21: 75-79.

74. Tabachnick KR, Menshenina LL, Lopes DA, Hajdu E (2009) Two new Hyalonema species (Hyalonematidae: Amphidiscosida) from eastern and south-eastern Brazil, and further Hexactinellida (Porifera) collected from seamounts off south-eastern Brazil by the RV ‘Marion Dufresne’ MD55 expedition. J Mar Biol Assoc UK 89: 1243-1250.

75. Tavares M (1999) The cruise of the Marion Dufresne off Brazilian coast: account of the scientific results and list of stations. Zoosystema 21: 597-605.

76. Yoneshigue-Valentin Y, Gestinari LM, Fernandes DR (2006) Macroalgas da Plataforma Continental de Salvador (estado da Bahia) ao Cabo de São Tomé (norte do estado do Rio de Janeiro), Brasil. In: Lavrado HP, Ignacio BL. Biodiversidade bentônica da região central da Zona Econômica Exclusiva brasileira. Rio de Janeiro: Museu Nacional - UFRJ. pp. 67-105.

77. Muricy G, Santos CP, Batista D, Lopes DA, Pagnoncelli D, et al. (2006) Filo Porifera. In: Lavrado HP, Ignácio BL. Biodiversidade bentônica da região central da Zona Econômica Exclusiva brasileira. Rio de Janeiro: Museu Nacional - UFRJ. pp. 109-145.

78. Zibrowius H (1987) Lês Coraux Stylasteridae et Scleractinia. In: Guille A, Ramos JM. Les Repports des campagnes à la mer TAAF MD55/Brésil à bord du Marion Dufresne 6 mai-2 juin 1987. Technical report 87-03. La Riche: Instaprint- Terres Australes et Antarctiques Françaises. pp. 132-136.

79. Castro CB, Thiago CM, Medeiros MS (2003) First record of the family Coralliidae (Cnidaria: Anthozoa: Octocorallia) from the western South Atlantic, with a description of Corallium medea Bayer, 1964. Zootaxa 323: 1-8.

80. Castro CB, Pires DO, Medeiros MS, Loiola LL, Arantes RC, et al. (2006) Cnidaria: Corais. In: Lavrado HP, Ignácio BL. Biodiversidade bêntica da costa central brasileira. Rio de Janeiro: Museu Nacional - UFRJ. pp. 147-192.

81. Leal JH (1991) Marine Prosobranch Gastropods from Oceanic Islands off Brazil: species composition and biogeography. Oegstgeet: Universal Book Services. 419

82. Gueron MC (1987) Les Mollusques Bivalves. In: Guille A, Ramos JM. Les Repports des campagnes à la mer TAAF MD55/Brésil à bord du Marion Dufresne 6 mai-2 juin 1987. Technical report 87-03. La Riche: Instaprint- Terres Australes et Antarctiques Françaises. pp. 140-141.

83. Absalão RS, Caetano CH, Fortes RR (2006) Filo Mollusca. In: Lavrado HP, Ignácio BL. Biodiversidade bentônica da região central da Zona Econômica Exclusiva brasileira. Rio de Janeiro: Museu Nacional - UFRJ. pp. 211-260.

84. Young PS (1999) The Cirripedia (Crustacea) collected by the RV Marion Dufresne along the Vitória-Trindade seamounts (Brazil). Zoosystema 21: 607-624.

85. Watling L, Gerken S (1999) Two new cumacean (Crustacea) species from the deep South Atlantic. Zoosystema 21: 661-669.

86. Wakabara Y, Serejo CS (1999) Amathillopsidae and Epimeriidae (Crustacea, Amphipoda) from bathyal depths off the Brazilian coast. Zoosystema 21: 625-645.

87. Serejo C, Young PS, Cardoso IA, Tavares CR, Abreu Jr CR (2006) Filo Arthropoda. Subfilo Crustacea. In: Lavrado HP, Ignácio BL. Biodiversidade bentônica da região central da Zona Econômica Exclusiva brasileira. Rio de Janeiro: Museu Nacional - UFRJ. pp. 299-337.

88. Larsen K (1999) A new species of the deap sea genus *Carpoapseudes* Lang from the southwestern Atlantic (Crustacea, Tanaidacea). Zoosystema 21: 647-659.

89. Albuquerque MN, Tavares M (1987) Les Crustaces. In: Guille A, Ramos JM. Les Repports des campagnes à la mer TAAF MD55/Brésil à bord du Marion Dufresne 6 mai-2 juin 1987. Technical report 87-03. La Riche: Instaprint- Terres Australes et Antarctiques Françaises. pp. 144-145.

90. Ventura CR, Lima RP, Nobre CC, Veríssimo I, Zama PC (2006) Filo Echinodermata. In: Lavrado HP, Ignácio BL. Biodiversidade bentônica da região central da Zona Econômica Exclusiva brasileira. Rio de Janeiro: Museu Nacional - UFRJ. pp. 339-389.

91. Guille A, Albuquerque MN (1990) Stratégie de dispersion et insularité: Les Ophiures littorales de la chaîne des seamounts Vitória-Trindade (Brésil), Résultats preliminaires. In: De Ridder C, Dubois C, Lahaye MC, Jangoux M. Echinoderm Research – Proceedings of the Second European Conference on Echinoderms, Brussels, 18-21, September 1989. Rotterdam: A Balkema Publishers. pp. 125-129.

92. Guille A, Albuquerque MN (1987) Les Echinodermes du Plateau Continental et des Seamounts. In: Guille A, Ramos JM. Les Repports des campagnes à la mer TAAF MD55/Brésil à bord du Marion Dufresne 6 mai-2 juin 1987. Technical report 87-03. La Riche: Instaprint- Terres Australes et Antarctiques Françaises. pp. 146-149.

93. Albuquerque MN, Guille A (1991) Ophiuroidea (Echinodermata) ao largo do Brasil: Banco dos Abrolhos, Cadeia Submarina Vitória-Trindade e Plataforma Continental Adjacente. Bol Mus Nac, NS Zool 353: 30.

94. Albuquerque MN, Campos LS, Guille A (2001) Two new species of Amphiuridae (Echinodermata: Ophiuroidea) from the southeast Brazilian Coast. Zoosystema 23: 591-604.

95. Bouchet P, Leal J (1987) Les Gasteropodes des Seamounts. In: Guille A, Ramos JM. Les Repports des campagnes à la mer TAAF MD55/Brésil à bord du Marion Dufresne 6 mai-2 juin 1987. Technical report 87-03. La Riche: Instaprint- Terres Australes et Antarctiques Françaises. p. 137.

96. Vaske Jr. T, Lima KL, Ribeiro AC, Lessa RP (2008) Record of the St. Helena deepwater scorpionfish, *Pontinus nigropunctatus* (Günther) (Scorpaeniformes: Scorpaenidae), in the Saint Peter and Saint Paul Archipelago, Brazil. PanamJAS 3: 46-48.

97. Olavo G, Costa PA, Martins AS (2005) Prospecção de grandes peixes pelágicos da região central da ZEE brasileira entre o Rio Real-BA e o Cabo de São Tomé-RJ. In: Costa PA, Martins AS, Olavo G. Pesca e potenciais de exploração de recursos vivos na região central da Zona Econômica Exclusiva brasileira. Rio de Janeiro: Museu Nacional, Série Livros n. 13. pp. 167-202.

98. Martins AS, Olavo G, Costa PA (2005) A pesca de linha de alto mar realizada por frotas sediadas no Espírito Santo, Brasil. In: Costa PA, Martins AS, Olavo G. Pesca e potenciais de exploração de recursos vivos na Região Central da Zona Econômica Exclusiva Brasileira. Rio de Janeiro: Série Documentos Revizee/Score Central, Museu Nacional. pp. 35-55.

99. Costa PA, Martins AS, Olavo G (2005) Pesca e potenciais de exploração de recursos vivos na Região Central da Zona Econômica Exclusiva Brasileira. 1 ed. Rio de Janeiro: Série Documentos Revizee/Score Central, Museu Nacional. 247 p.
