# Supplementary material for: Marine Biodiversity in the Atlantic and Pacific Coasts of South America: Knowledge and Gaps
Source: PLoS One. 2011 Jan 31;6(1):e14631. doi: 10.1371/journal.pone.0014631 (PMC3031619; doi:10.1371/journal.pone.0014631)
Supplement: Table S8 — Major Brazilian cruises that have taken samples in the deep sea, including seamounts and abyssal plains. (0.06 MB DOC) [file pone.0014631.s008.doc]

Table S8. Major Brazilian cruises that have taken samples in the deep sea, including seamounts and abyssal plains.

| **Vessels** | **Year** | **Depth range (m)** | **Study area** | **Sampling** | **References** |
| --- | --- | --- | --- | --- | --- |
| Marion Dufresne | 1987 | 60 - 5500 | Cabo Frio to Abrolhos continental slope | Pelagic, plankton, sediment, benthos | [1-2] |
| Prof. W. Besnard | 1987 | Down to 600 | SE – off São Paulo | Trawling, dredging, BC | [3-4] |
| Atlântico Sul | early 1990’s | Down to 1000 | South – off Rio Grande do Sul | Dredging, trawling | [5-9] |
| Victor Hensen | 1995 | 1300 | NE and SE Brazilian offshore | Pelagic and benthic systems | [10-13] |
| Prof. W. Besnard | 2001 | 600-700 | Santos Basin | Geology, hydrology, phytoplankton, zooplankton, ichtyoplankton, benthos; BC, trawling | [14] |
| Atlantico Sul, Prof. W. Besnard, Astro Garoupa, Antares, Thalassa | 1996 - 2002 | Shallow down to 2000 | Whole of Brazilian coast, deeper stations mainly at the SE offshore, but also deep-sea fishing in the North and Northeast regions | Pelagic and benthic systems; BC, dredging, trawling, long-line and other sampling gear | [15-29] |
| Astro Garoupa, Prof. W. Besnard | 2001 - 2003 | 700 - 2000 | Campos Basin | Pelagic and benthic systems, BC, trawling | [28-35] |
| ? | 2001 -2003 | 900 | Campos Basin | Geological; Chemical; Biological (benthic - meiofauna and macrofauna) | [36-39] |
| Stolt Offshore, and several others depending on contracts | From 2005 to present | 700 - 1100 | Campos Basin | SCV 3000 and other ROVs, depending on contracts | [30-31, 39-41] |
| R/V Gyre, R/V Miss Emma McCall and LukeThomas | From 2008 to present | Continental shelf down to 3000 | Campos Basin | Pelagic and benthic systems, BC, trawling | Ongoing project |

[1] Guille, A. & Ramos, J.M. 1987. Les rapports des campagnes à la mer MD55/Brésil à bord du “Marion Dufresne” – 6 mai-2 juin 1987. Terres Australes et Antarctiques Françaises, 87(3): 1-198.

[2] Tavares, M. 1999. The cruise of the Marion Dufresne off Brazilian coast: account of the scientific results and list of stations. Zoosystema 21 (4): 597-605.

[3] Pires-Vanin, A. M. S. 1993. A Macrofauna Bentica da Plataforma Continental ao Largo de Ubatuba, Sao Paulo, Brasil. Publicação Especial Do Instituto Oceanográfico, v. 10, p. 137-158.

[4] Sumida, P. Y. G. & Pires-Vanin, A. M. S. 1997. Benthic associations of the shelfbreak and upper slope off Ubatuba-SP, southeastern Brazil. Estuarine, Coastal and Shelf Science, Londres, v. 44, p. 779-784.

[5] Melo, G.A.S., 1999. Manual de identificação dos Crustacea Decapoda do litoral brasileiro: Anomura, Thalassinidea, Palinuridea, Astacidea. São Paulo, Plêiade/ FAPESP, 551 p.

[6] Capítoli, R.R. & Bemvenuti, C. 2006. Associações de Macro-Invertebrados Bentônicos de Fundos Inconsolidados da Plataforma Continental e Talude Superior no Extremo Sul do Brasil. Atlântica, Rio Grande, 28(1): 47-59.

[7] Kitahara, M.V. 2006. Novas ocorrências de corais azooxantelados (Anthozoa, Scleractinia) na plataforma e talude continental do sul do Brasil (25-34o S). Biotemas, 19 (3): 55-63.

[8] Mothes, B. & Silva, C.M.M. 2002. *Stellata uetzleri* sp Nov., a new ancorinid from the Southwestern Atlantic (Porifera: Astrophorida). Scientia Marina, 66(1): 69-75.

[9] Mothes, B.; Hajdu, E.; Lerner, C.B. & van Soest, R.W.M. 2004. New species of *Ulosa* and *Biemna* (porifera, Demospongiae, Poecilosclerida) from the N-Ne Brazilian continental shelf. Bollettino dei Musei e degli Istituti Biologici dell'Università di Genova, Gênova, Itália, 68: 477-482.

[10] Ekau, W. & Matsuura, Y. 1996. Diversity and Distribution of Ichthyoplankton in the Continental Shelf Waters of East Brazil. In: Ekau, W & Knoppers, B. (eds). Sedimentation Process and produtivity in the Continental Shelf Waters off East and Northeast Brazil. Joint Oceanographic Projects. JOPS II, Cruise Report and First Results. Center for Tropical Marine Ecology, Bremen, pp. 135-147.

[11] Gomes, A.S.; Abreu, C.M.R.C.; Absher, T.M. & Figueiredo, A.G. 1999. Abiotic features and the abundance of macrozoobenthos of continental margin sediments of East Brazil. Archive of Fishery and Marine Research, 47 (1-3): 321-334.

[12] Ekau, W. & Knoppers, B. 2003. A review and redefinition of the large marine ecosystems of Brazil. In: K. Sherman and G. Hempel (eds.). Large Marine Ecosystems of the World --Trends in Exploitation, Protection and Research . Elsevier Science. Amsterdam. ISBN: 0444510273.

[13] Benkendorfer, G. & Soares-Gomes, A. 2009. Biogeography and biodiversity of gastropod molluscs from the eastern Brazilian continental shelf and slope. Lat. Am. J. Aquat. Res., 37(2): 143-159.

[14] Rossi-Wongtschowski, C.L.D.B. & Madureira, L.S.P. (Eds.) 2006. O Ambiente Oceanográfico da Plataforma Continental e do Talude na Região Sudeste-Sul do Brasil. São Paulo, Editora da Universidade de São Paulo, 472 p.

[15] Figueiredo, J. L., Santos, A. P., Yamaguti, N., Bernardes, R. A. & Rossiwongtschowski, C. L. D. B. 2002. Peixes da zona econômica exclusiva da região sudeste-sul do Brasil: Levantamento com rede de meia água. São Paulo: Edusp: 248p.

[16] Amaral, A.C.Z.; Lana, P.C.; Fernandes, F.C. & Coimbra, J.C. 2004. Caracterização do ambiente e da macrofauna bentônica. Parte I. In: Amaral, A.C.Z. & Rossi-Wongtschowski, C.L.D.B. (Eds.). 2004. Biodiversidade bentônica das regiões sudeste e sul do Brasil – Plataforma externa e Talude superior. Série Documentos Revizee: Score-Sul. Série documentos Revizee: Score Sul.. 1 ed. São Paulo, SP: Instituto Oceanográfico - USP, v. 1., pp 11-46.

[17] Amaral, A.C.Z. & Jablonski, S. 2005. Conservation of Marine and Coastal Biodiversity in Brazil. Conservation Biology, 19(3): 625-631.

[18] Amaral, A.C.Z.; Rizzo, A.E. & Arruda, E.P. 2005. Manual de Identificação dos Invertebrados Marinhos da Região Sudeste-Sul do Brasil. Vol. 1, 1ª ed., Editora da Universidade de São Paulo, São Paulo, 287 p.

[19] Bernardes, R. A. ; Rossi-Wongtschowski, C. L. B. ; Wahrlich, R. ; Vieira, R. C. ; Santos, A. P. & Rodrigues, A. R. 2005a. Prospecção pesqueira de recursos demersais com armadilhas e pargueiras na Zona Econômica Exclusiva da Região Sudeste-Sul do Brasil. 1. ed. São Paulo: Instituto Oceanográfico, v. 1. 112 p.

[20] Bernardes, R. A.; Figueiredo, J. L.; Rodrigues, A. R.; Fischer, L. G.; Vooren, C. M.; Haimovici, M. & Rossi-Wongtschowski, C. L. B. 2005b. Peixes da Zona Econômica da Região Sudeste-Sul do Brasil: levantamento com armadilhas, pargueiras e rede de arrasto de fundo. 1. ed. São Paulo: EDUSP, v. 1. 295 p.

[21] Costa, P.A.S.; Martins, A.S. & Olavo, G. 2005a. Pesca e potenciais de exploração de recursos vivos na Região Central da Zona Econômica Exclusiva Brasileira. Série Documentos Revizee/Score Central, Museu Nacional, Rio de Janeiro, 247p.

[22] Costa, P.A.S.; Olavo, G. & Martins, A.S. 2005b. Áreas de pesca e rendimentos da frota de linheiros na região central da costa brasileira entre Salvador-BA e o Cabo de São Tomé-RJ. In: Costa, P.A.S.; Martins, A.S. & Olavo, G. Pesca e potenciais de exploração de recursos vivos na Região Central da Zona Econômica Exclusiva Brasileira. Série Documentos Revizee/Score Central, Museu Nacional, Rio de Janeiro, pp. 57-70.

[23] Bonecker, S. L. C. (Ed.) 2006. Atlas de zooplâncton da região central da Zona Econômica Exclusiva Brasileira. Museu Nacional, Rio de Janeiro. 234 p.

[24] Bonecker, A. C. T.; Castro, M. S. (eds.) 2006. Atlas de larvas de peixes da região central da Zona Econômica Exclusiva Brasileira. Rio de Janeiro: Museu Nacional, 2006. (Série Livros; 19) 216 p. ISBN 85-7427-013-X

[25] Lavrado, H.P.; Ignacio, B.L. 2006. Biodiversidade bentônica da região central da Zona Econômica Exclusiva Brasileira. Vol. 1, 1ª ed., Museu Nacional - UFRJ, Rio de Janeiro, 389 p.

[26] Costa, P.A.S.; Olavo, G. & Martins, A.S. (Eds) 2007. Biodiversidade da fauna marinha profunda na costa Brasileira. Rio de Janeiro: Museu Nacional, Série Livros n. 24, 184p.

[27] Lavrado, H.P.; Viana, M.S. 2007. Atlas de invertebrados marinhos da região central da Zona Econômica Exclusiva Brasileira - parte 1. Vol. 1, 1ª ed., Museu Nacional da UFRJ, Rio de Janeiro, 258 p.

[28] Arantes, R. C. M.; Castro, C. B.; Pires, D. O. & Seoane, J. C. S. 2009. Depth and water mass zonation and species associations of cold-water octocoral and stony coral communities in the southwestern Atlantic. Marine Ecology Progress Series, 397: 71–79.

[29] Pires, D.O.; Castro, C.B. & Silva, J.C. 2009. Reproductive biology of the deep-sea pennatulacean *Anthoptilum murrayi* (Cnidaria, Octocorallia). Marine Ecology Progress Series, 397: 103–112.

[30] Campos, L.S.; Moura, R.B.; Alcântara, P.F.; Vasconcelos, R.F.; Curbelo-Fernandez, M.P.; Veríssimo, I.; Cavalcanti, G.H., 2010a. On the two new records of Family Brisingidae (Echinodermata: Asteroidea) from the Brazilian continental margin. In: Harris, L.G.; Bottger, S.A.; Walker, C.W.; Lesser, M.P. (Eds) Echinoderms: Durham. CRC Press / Taylor & Francis Group, pp. 139-146.

[31] Campos, L.S.; Moura, R.B.; Souza, I.; Fernandez, M.P.C.; Cavalcanti, G. H. & Brasil, A.C.S. 2010b. ROV imaging of deep-sea echinoderms from the Brazilian continental margin, Southwest Atlantic. In: L.G. Harris; S.A. Bottger; C.W. Walker & M.P. Lesser (Eds.) Echinoderms: Durham, New Hampshire: CRC Press / Balkema Taylor & Francis Group, pp. 147-152.

[32] Campos, L. S.; Barboza, C.A.M.; Alcântara, P.F.; Moura, R.B.; Frensel, R. & Wanderley, P. (In Press). Filo Echinodermata. In: Projeto de Caracterização Ambiental de Águas Profundas da Bacia de Campos/ PETROBRAS, livro 2 - Atlas, ENPES/PETROBRAS.

[33] Falcão, A.P.C.; Curbelo Fernandez, M.P.; Lavrado, H.P.; Ferreira, V.P.R.; Campos, L. S.; França, M.R. & Morosko, E. (in press). Antecedentes, Histórico e Descritivo do Projeto de Caracterização Ambiental de Águas Profundas da Bacia de Campos/Petrobras. In: Projeto de Caracterização Ambiental de Águas Profundas da Bacia de Campos/ PETROBRAS, livro, CENPES / PETROBRAS.

[34] Lavrado, H.P.; Brasil, A.C.S.; Curbelo Fernandez, M.P. & Campos, L.S. (2010a). Aspectos gerais da fauna demersal da Bacia de Campos. In: Lavrado, H.P. & Brasil, A.C.S. (Orgs). Biodiversidade da região oceânica profunda da Bacia de Campos: Megafauna e Ictiofauna demersal. Rio de Janeiro: SAG Serv., pp 21-29.

[35] Lavrado, H.P.; Brasil, A.C.S.; Curbelo Fernandez, M.P. & Campos, L.S. (2010b). Aspectos gerais da macrofauna bentônica da Bacia de Campos. In: Lavrado, H.P. & Brasil, A.C.S. (Orgs). Biodiversidade da região oceânica profunda da Bacia de Campos: Macrofauna. Rio de Janeiro: SAG Serv., pp. 19-27.

[36] Toldo Jr, E.E. & Zouain, R.N.A. 2009. Environmental monitoring of offshore drilling for petroleum exploration (MAPEM): A brief overview. Deep-Sea Research, Part II, 56(1-2): 1-3.

[37] Santos, M.F.L.; Lana, P.C.; Silva, J.; Fachel, J.G. & Pulgati, F.H. 2009. Effects of non-aqueous fluids cuttings discharge from exploratory drilling activities on the deep-sea macrobenthic communities. Deep Sea Research Part II: Topical Studies in Oceanography, MAPEM, 56 (1-2): 32-40.

[38] Netto, S.A.; Gallucci, F. & Fonseca, G. 2009. Deep-sea meiofauna response to synthetic-based drilling mud discharge off SE Brazil Deep Sea Research Part II: Topical Studies in Oceanography, MAPEM, 56 (1-2):41-49.

[39] Moura, R.B.; Campos, L. S.; Fernandez, M.P.C.; Cavalcanti, G. H. & Falcão, A.P.C. 2010. Synallactidae (Echinodermata, Holothuroidea) from Campos Basin, Southwest Atlantic. In: Echinoderms: Durham, L.G. Harris; S.A. Bottger; C.W. Walker & M.P. Lesser (Eds), New Hampshire, USA: CRC Press / Balkema Taylor & Francis Group, pp. 245-250.

[40] Tavares, M.; Melo-Filho, G.A.S. & Melo, G.A.S. 2008. The deep-sea squat lobster *Munidopsis transtridens* Pequegnat and Pequegnat, 1971 (Decapoda: Anomura: Galatheidae) from the Southwestern Atlantic. Nauplius, 16(2): 95-99.

[41] Tabachnick, K.R.; Menshenina, L.L.; Lopes, D.A. & Hajdu, E. 2009. Two new Hyalonema species (Hyalonematidae: Amphidiscosida) from eastern and south-eastern Brazil, and further Hexactinellida (Porifera) collected from seamounts off south-eastern Brazil by the RV ‘Marion Dufresne’ MD55 expedition. Journal of the Marine Biological Association of the United Kingdom, 89:1243-1250.
